# NORHA, a novel follicular atresia-related lncRNA, promotes porcine granulosa cell apoptosis via the miR-183-96-182 cluster and FoxO1 axis

**DOI:** 10.1186/s40104-021-00626-7

**Published:** 2021-10-07

**Authors:** Wang Yao, Zengxiang Pan, Xing Du, Jinbi Zhang, Honglin Liu, Qifa Li

**Affiliations:** College of Animal Science and Technology, Nanjing Agricultural University, Nanjing, 210095 China

**Keywords:** Follicular atresia, Granulosa cell apoptosis, ncRNA NORHA, Oxidative stress

## Abstract

**Background:**

Follicular atresia has been shown to be strongly associated with a low follicle utilization rate and female infertility, which are regulated by many factors such as microRNAs (miRNAs), which constitute a class of noncoding RNAs (ncRNAs). However, little is known about long noncoding RNAs (lncRNAs), which constitute another ncRNA family that regulate follicular atresia.

**Results:**

A total of 77 differentially expressed lncRNAs, including 67 upregulated and 10 downregulated lncRNAs, were identified in early atretic follicles compared to healthy follicles by RNA-Sequencing. We characterized a noncoding RNA that was highly expressed in atretic follicles (NORHA). As an intergenic lncRNA, NORHA was one of the upregulated lncRNAs identified in the atretic follicles. To determine NORHA function, RT-PCR, flow cytometry and western blotting were performed, and the results showed that NORHA was involved in follicular atresia by influencing GC apoptosis with or without oxidative stress. To determine the mechanism of action, bioinformatics analysis, luciferase reporter assay and RNA immunoprecipitation assay were performed, and the results showed that NORHA acted as a ‘sponge’, that directly bound to the miR-183-96-182 cluster, and thus prevented its targeted inhibition of FoxO1, a major sensor and effector of oxidative stress.

**Conclusions:**

We provide a comprehensive perspective of lncRNA regulation of follicular atresia, and demonstrate that NORHA, a novel lncRNA related to follicular atresia, induces GC apoptosis by influencing the activities of the miR-183-96-182 cluster and FoxO1 axis.

**Supplementary Information:**

The online version contains supplementary material available at 10.1186/s40104-021-00626-7.

## Introduction

Long noncoding RNAs (lncRNAs) constitute a subclass of RNA polymerase II transcripts with a length of not less than 200 nucleotides (nt) and weak or noncoding potential [1, 2]. Compared with protein-coding genes and other noncoding RNAs (ncRNAs), the lack of sequence conservation among species is a prominent feature of lncRNAs [3]. This lack of conservation has confounded efforts to predict the sequences, functions, and mechanisms of action of lncRNAs across species [4]. For scientists, species-specific lncRNAs are mysterious treasures, therefore, high-throughput technology is used to discover species-specific lncRNAs, which is a focus of current research, and many of lncRNAs in various species have been identified [5]. Only a few lncRNAs have been functionally characterized, however, and as of 2018, biological functions of only 156 lncRNAs had been identified [3, 6]. Their known functions are very extensive, and they have been reported to be essential regulators in various cell biological processes, such as cell survival [7], differentiation [8], cell cycle [9], cell apoptosis [10], pluripotency [11], and susceptibility to infection [12].

Emerging data have demonstrated that in the ovary lncRNAs are abundant in oocytes and, various somatic cell types, including granulosa cells (GCs), cumulus cells (CCs), and ovarian surface epithelial stem cells, and follicular fluid [13, 14]. Numerous lncRNAs have been identified in somatic cells and oocytes at all stages of follicular development through high-throughput technology. For example, 20,563 lncRNAs were identified in human CCs [15], and 4,926 differentially expressed lncRNAs (DELs) were identified in goat ovaries between the luteal and follicular phases [16]. However, only a few lncRNAs have been well-characterized in terms of function and mechanism in humans and rodents [17, 18]. For instance, nuclear enriched abundant transcript 1 (NEAT1) is a lncRNA involved in many major physiological events [19, 20]. In mammalian ovaries, NEAT1 is highly expressed in human metaphase II (MII) oocytes and mouse corpus luteum, and luteal tissue formation was seriously diminished in nearly one-half of a Neat1 knockout mice [13, 17]. Furthermore, ovarian lncRNAs have been shown to be related to livestock fecundity [21] and human infertility [22].

Follicular maturation leads to ovulation and atresia leads to follicular degeneration [23, 24]. The latter is a limiting factor of female reproduction [25, 26] and since lncRNA profiling of healthy and atretic follicles has not been reported, the current study is focused on the identification of lncRNAs related to follicular development. Therefore, we first performed RNA-Seq to investigate lncRNA expression profiles during follicular atresia of pig ovaries. Furthermore, we investigated the role of NORHA, a lncRNA that is highly expressed in early atretic follicles (EAF). In addition, we also revealed the mechanism of action of NORHA mediated by competition with FoxO1 for the miR-183-96-182 cluster binding sites.

## Materials and methods

### Experimental design

RNA-Seq was performed with healthy follicles (HF) and EAF to detect lncRNA profiles during atresia, and qRT-PCR was used to verify the RNA-Seq results. We obtained the full-length NORHA by RACE assay, and bioinformatics analysis, subcellular localization assays and qRT-PCR in follicles were performed to clarify the biological characteristics of NORHA. Flow cytometry and western blotting were performed to confirm the function of NORHA in GCs. Bioinformatics analysis, dual-luciferase reporter assay and RIP assay were used to confirm the interaction of NORHA and the miR-183-96-182 cluster, and FoxO1 was found to be a target of the miR-183-96-182 cluster in porcine GCs through dual-luciferase reporter assay, qRT-PCR and western blot analysis. Cotransfection assays were performed to show that NORHA induced FoxO1-mediated GC apoptosis by competing for miR-183-96-182 cluster binding sites, and a model of oxidative stress-induced GC apoptosis was established by cell treatment with H_2_O_2_ (Fig. S1).

### Animals

Healthy and mature Duroc-Yorkshire-Landrace sows (~ 180 d and ~ 110 kg) were obtained from Shunzhu (Nanjing, China) for ovary isolation and antral follicles collection. Besides, nine tissues including heart, liver, spleen, lung, kidney, stomach, intestine, muscle and ovary were also collected for tissue expression profile analysis. All animal experiments involved in this study were performed followed ARRIVE guidelines and were approved by the Ethical Committee of Nanjing Agricultural University.

### Follicles collection and classification

For detection of lncRNA profiles during porcine follicular atresia, we first isolated and classified ovarian follicles. Briefly, antral follicles with a diameter of 3–5 mm were isolated from ovaries, and then classified into HF and EAF groups based on the ratio of progesterone (P4)/17β-estradiol (E2) levels in follicular fluid, GC density (GC number per ml of follicular fluid), and the morphological features of the follicles as previously described [27]. The concentrations of P4 and E2 in follicular fluid were detected by radioimmunoassay (RIA) with an iodine [^125^I] P4 or E2 radioimmunoassay kit (North Institute, Beijing, China). The density of GCs was determined using a hemocytometer (Qiujing, Shanghai, China). Translucent follicles with extensive vascularization, GC density < 4,000 cells/mL and a P4/E2 ratio < 1 were considered as HF, while opaque follicles with poor vascularization, 4,000 cells/mL ≤ GC density < 10,000 cells/mL and 1 ≤ the P4/E2 ratio < 5 were classified as EAF.

### RNA isolation and sequencing

TRIzol reagent (Invitrogen, Shanghai, China) was used to isolate total RNA from follicles. The quality and integrity of purified RNA were evaluated by using an Agilent 2100 Bioanalyzer (Agilent, California, USA). The integrity score was no less than 7.0, and a 28S/18S ratio of ribosomal RNA greater than 0.7 was set as the acceptable standard. After removing the rRNA by using the Epicentre Ribo-Zero rRNA removal kit (Illumina, San Diego, USA), the RNA was fragmented, end repaired, adapter ligated, PCR amplified and purified according to the instructions of a NEBNext Ultra RNA Library Prep Kit for Illumina (NEB, Beijing, China). Libraries were paired-end sequenced by using an Illumina HiSeq 3000 PE150 platform (Illumina) at RiboBio Co. (RiboBio, Guangzhou, China). An Agilent 2200 TapeStation (Agilent) was used for quality control of RNA sequencing. After removing the joint contaminated sequences, and sequences with missing or low-quality bases, clean filtered reads were obtained.

### Sequencing data analysis

Genome mapping of clean reads was performed by using TopHat v2.0.9 software (https://ccb.jhu.edu/software/tophat/index.shtml). The mapped reads were assembled by using the Cufflinks tool (http://cole-trapnell-lab.github.io/cufflinks/). The Cuffcompare program (http://cole-trapnell-lab.github.io/cufflinks/cuffcompare/) was used to compare candidate sequences with known lncRNAs, and differential expression analysis was performed by DESeq2 in the R package (https://www.r-project.org/). LncRNA identification and DEL screening were performed by the following criteria: (i) transcript length ≥ 200 bp; (ii) expression value of FPKM > 0; (iii) prediction as a noncoding RNA in the pig reference genome database (*S. scrofa* 11.1); and (iv) *P*-value ≤ 0.05 and |logarithm of the fold-change between two groups| ≥ 1.

### Functional enrichment analysis

To understand the potential functions of the DELs, the *cis*-regulatory mRNAs, which were selected within a range of 200 kb upstream or downstream of the DEL locus, were annotated and classified by Gene Ontology (GO) and Kyoto Encyclopedia of Genes and Genome (KEGG) pathway analysis with the DAVID Bioinformatics Resources v6.7 (https://david-d.ncifcrf.gov/). The significantly enriched GO terms or KEGG pathways (*P* < 0.05) were chosen to build bar charts and scatter charts with R software.

### Quantitative real-time RT-PCR

To validate the RNA-Seq data, and investigate the tissue expression pattern of the lncRNA NORHA and the expression levels of genes including caspase 3, FoxO1, miR-183, miR-96, miR-182, U6 and GAPDH, total RNA from tissues (heart, liver, spleen, lung, kidney, stomach, intestine, muscle and ovary), follicles or GCs was extracted as described, and 500 ng of total RNA was collected to synthesize first-strand cDNA of protein-coding genes using PrimeScript RT Master Mix (TaKaRa, Dalian, China). In addition, reverse transcription of miRNAs was performed with TransScript miRNA First-Strand cDNA Synthesis SuperMix (TransGen, Beijing, China). RT-PCR was performed with AceQ RT-PCRSYBR Green Master Mix (Vazyme, Nanjing, China) on a QuantStudio 7 Flex Real-Time fluorescent quantitative PCR system (Applied Biosystems, MA, USA). The expression levels of GAPDH and U6 were used as the internal controls for the protein-coding genes and miRNAs, respectively. The primer sequences used for RT-PCR are shown in Table S6.

### Rapid amplification of cDNA ends

To obtain the full-length lncRNA NORHA, 5′- and 3′-rapid amplification of cDNA ends (RACE) assays were performed. In brief, first-strand cDNA was synthesized by using a SMARTer RACE cDNA amplification kit (TaKaRa, Dalian, China). Subsequently, PCR was performed to amplify the cDNA product from the 5′ end or 3′ end of NORHA with the gene-specific primers shown in Table S6. The amplification products were identified using 1.5% agarose electrophoresis, further purified and inserted into a pMD18-T vector (Qingke, Nanjing, China), and then sequenced by Sangong (Shanghai, China).

### Bioinformatics analysis

The chromosomal locations of transcripts, BLAST, annotated transcripts and sequence mapping were visualized on the basis of the genome data obtained from the NCBI database (https://www.ncbi.nlm.nih.gov/gene). Two online tools, CPC (http://cpc.cbi.pku.edu.cn/) and CAPT (http://lilab.research.bcm.edu/cpat/index. php), were used to evaluate the coding potential of the transcripts. An interaction network diagram of NORHA and miRNAs was constructed with Cytoscape_v3.5.1 software (https://cytoscape.org/), and the miRNAs downregulated in atretic follicles (an expression pattern opposite that of NORHA) according to our small-RNA-Seq data (data not shown) were considered to have a potential regulatory relationship with NORHA. Subsequently, RNAhybrid (https://bibiserv.cebitec.uni-bielefeld.de/ rnahybrid/), an online tool, was used to predict the binding sites of the identified miRNAs within the NORHA transcript and calculate the minimum free energy (MFE) of the interaction between NORHA and atresia-related miRNAs.

### Cell culture and treatment

Porcine GCs used for qRT-PCR, western blot, flow cytometry, RIP and ChIP assays were isolated from fresh follicles with a 3–5 mm diameter by 10 mL syringes. After washing with phosphate-buffered saline (PBS) twice and centrifugation at 1,000 r/min for 5 min, the GCs were seeded into cell plates filled with DMEM/F-12 (Gibco, CA, USA) supplemented with 15% fetal bovine serum (Gibco), 100 U/mL penicillin and 100 μg/mL streptomycin (Gibco). The cells were then cultured in a humid atmosphere at 37 °C with 5% CO_2_. HEK293T cells for dual-luciferase assay were cultured in Dulbecco’s modified Eagle medium (DMEM, HyClone, UT, USA) supplemented with 10% (v/v) fetal bovine serum (Gibco), streptomycin (100 U/mL) and penicillin (100 μg/mL) (Gibco). Lipofectamine 3000 reagent (Invitrogen, Shanghai, China) was used for oligonucleotide transfection. After the cell density reached 70%–80%, Liposomes 3000, plasmids, mimics, inhibitors or siRNAs were mixed in 200 μL of Opti-MEM (Gibco), and this transfection reagent complex was evenly dropped into cells cultured *in vitro*. For H_2_O_2_ treatment, the medium was replaced with serum-free medium for 8–12 h, and then H_2_O_2_ (30% [w/w] in H_2_O, Sigma, Shanghai, China) at different concentrations was added to the medium and incubated for 90 min.

### Plasmid construction

To confirm the function of NORHA, we constructed the overexpression plasmid pcDNA3.1-NORHA, and full-length NORHA was amplified from the cDNA of porcine GCs by using a specific primer (Table S6) with *Hind*III and *Xho*I enzyme adaptors. After digestion and purification, the PCR product was then inserted into a pcDNA3.1 vector (GenePharma, Shanghai, China). In addition, a pcDNA3.1-FoxO1 vector was synthesized by YiDao (Nanjing, China). To identify the MREs (miRNA response elements) of the miR-183-96-182 cluster in NORHA or the FoxO1 3′-UTR, fragments of the FoxO1 3′-UTR and NORHA containing miRNA-binding sites were amplified and cloned into a pmirGLO dual-luciferase vector (Promega, Madison, WI, US). In addition, the MREs within the FoxO1 3′-UTR or NORHA were mutated using a Mut Express II Fast mutagenesis kit (Vazyme, Nanjing, China). All recombinant plasmids were verified by Sanger sequencing (Shanghai, China). The primers used for plasmid construction are listed in Table S6.

### Oligonucleotides

siRNAs specific: 1/ to pig NORHA and FoxO1, and 2/ to mimics and inhibitors of miR-183, miR-96 and miR-182 (Tab. S7) were generated by GenePharma (Shanghai, China).

### Apoptosis analysis

After transfection with plasmids, siRNAs, mimics or inhibitors, or treatment with H_2_O_2_, porcine GCs were digested in 0.25% trypsin at 37 °C without ethylenediaminetetraacetic acid (EDTA) for 2 min and then collected by centrifuging at 1,000 r/min for 5 min. After washing with cold PBS twice, the GCs were stained in 500 μL of Annexin V binding buffer with 5 μL of Annexin FITC V and 5 μL of PI solution (Vazyme, Nanjing, China) for 10 min. The apoptosis rate of the GCs was determined by a BD FACScan flow cytometer (Becton Dickinson, Franklin, NJ, USA), and data analysis was performed with FlowJo v10 software (Stanford University, CA, USA).

### Western blotting

After washing in cold PBS twice, GCs were treated with RIPA lysis buffer (Beyotime, 243 Shanghai, China) containing 1.5% proteasome inhibitor (PMSF, Solarbio, Beijing, China) for 20 min to isolate total protein. The concentration of each protein sample was quantified by using a BCA Kit (Biosharp, Beijing, China). A total of 15 μg of protein from each sample was loaded on 15% polyacrylamide gel and separated by sodium dodecyl sulfate-polyacrylamide gel electrophoresis (GenScript, Nanjing, China). After separation by electrophoresis, the proteins were transferred to polyvinylidene difluoride (PVDF) membranes (Millipore, Billerica, MA, USA). The membranes were blocked in 5% nonfat milk for 1 h and then incubated with primary antibodies for 8 h at 4 °C. The bound primary antibody was visualized with secondary antibody using an ECL detection reagent (Advansta, CA, USA) with a LAS2000 imaging system (GE Healthcare, Chicago, IL, USA). The β-tubulin protein level was used as the internal control. The primary antibodies used in the study were anti-Caspase 3 (CASP3) (diluted at 1:1,000; Proteintech, Wuhan, China), anti-FoxO1 (diluted at 1:1,000; CST, BMA, USA), and anti-β-tubulin (diluted at 1:2,000; Proteintech, Wuhan, China).

### Dual-luciferase reporter assay

Dual-luciferase reporter assay was performed in HEK293T cells. To investigate whether the miR-183-96-182 cluster binds to NORHA or the FoxO1 3′-UTR, a dual-luciferase reporter assay was performed. After cotransfection with luciferase plasmid and mimics, inhibitor, siRNA or overexpression vector for 24 h, cells were lysed with passive lysis buffer and centrifuged at 12,000 r/min for 5 min. Luciferase activity levels were detected with Luciferase Assay Buffer II and a Stop & GLO Substrate with Modulus assay system (Turner Biosystems, San Francisco, CA, USA) as previously described [28]. The relative luciferase activity of each sample was calculated as the ratio of Renilla/firefly luciferase intensity level.

### RNA immunoprecipitation (RIP) assay

To investigate the interaction of NORHA and miRNAs, RIP experiments were performed using an EZ Magna RIP kit (Millipore, Billerica, MA, USA). Briefly, porcine GCs were completely pelleted and lysed in RIP lysis buffer containing protease inhibitor cocktail and RNase inhibitor. Homogenates were resuspended in a single cell suspension and stored at − 80 °C. Magnetic beads were washed several times with RIP wash buffer and then labeled with antibodies for 30 min. The extract was incubated with magnetic beads linked to anti-AGO2 (CST, BMA, USA) or IgG antibodies (Santa Cruz, CA, USA) overnight at 4 °C with head-to-head rotation. Finally, qRT-PCR was performed to detect the expression of NORHA using the specific primers shown in Table S6.

### ROS detection

To investigate whether H_2_O_2_ induces oxidative stress in GCs, ROS detection was performed using a reactive oxygen species assay kit (KeyGen, Shanghai, China). Briefly, GCs were incubated with DCFH-DA (diluted at 1:1,000 in serum-free medium) at 37 °C for 20 min. Cells without DCFH-DA treatment were used as negative controls. After washing twice with serum-free medium, the fluorescence of the DCF in the cells was detected at an excitation wavelength of 488 nm with a BD FACScan flow cytometer (Becton Dickinson, Franklin, NJ, USA).

### Statistical analysis

Statistical analysis was performed by using GraphPad Prism v5.0 software (San Diego, CA, USA). The significance among different groups was analyzed by Student’s *t*-test (between two groups) and one-way analysis of variance test (three or more groups). A *P* value < 0.05 indicates a significant difference. Correlation was determined using the Pearson test model. A *P* value < 0.05 indicates an association, and the r value represents the level of correlation.

## Results

### Genome-wide identification of follicular atresia-related lncRNAs

**Fig. 1:**
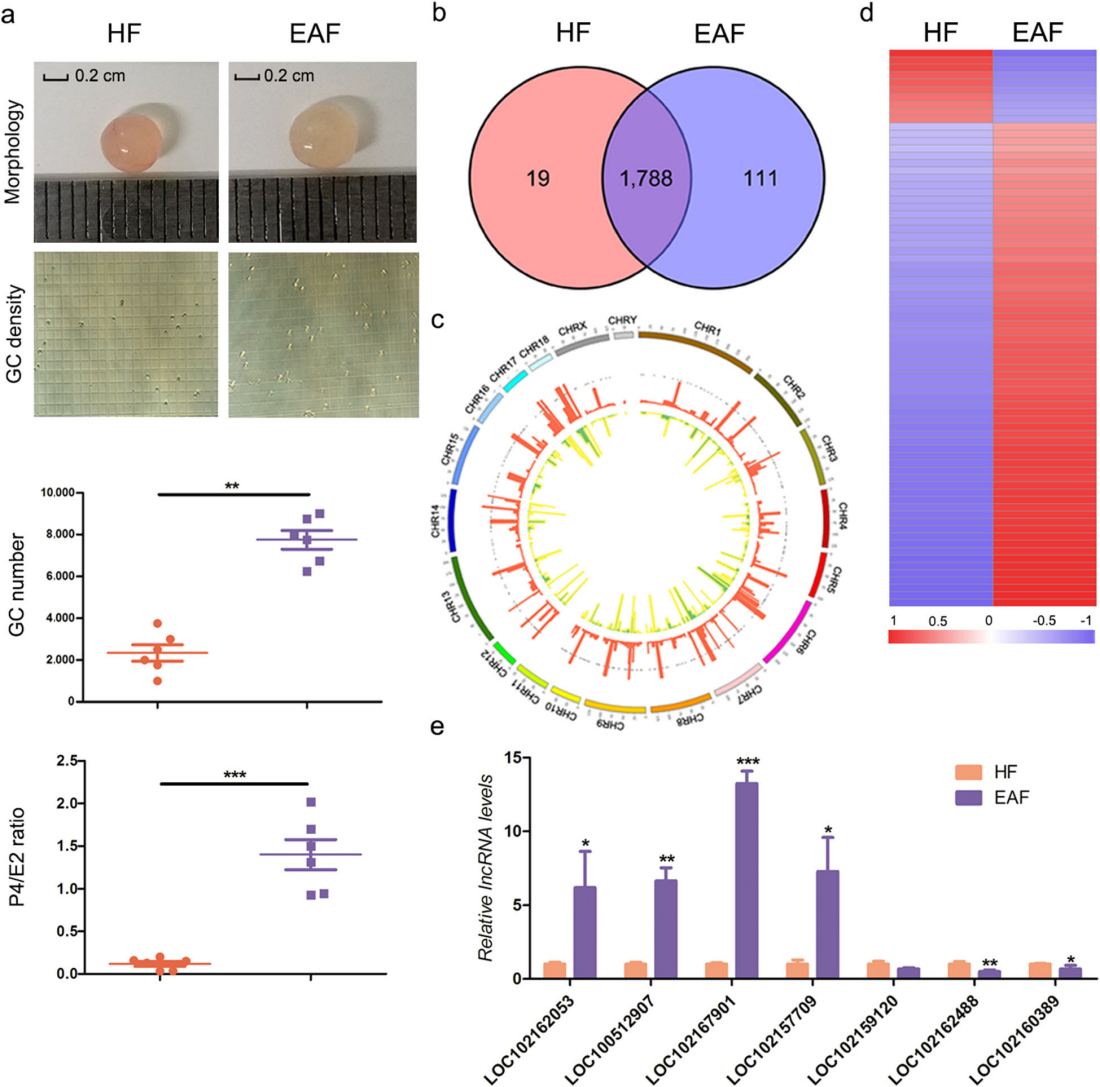
lncRNA profiles in ovarian follicles. **a** Porcine ovarian follicles (3–5 mm diameter) were classified into healthy follicles (HF) and early atresia follicles (EAF). **b** Venn diagram of the overlapping lncRNAs in HF and EAF. **c** Chromosome distribution and expression schema of lncRNAs. The outermost layer represents chromosomes, and the gray dots represent the chromosomes corresponding to each lncRNAs. The red and yellow histograms represent lncRNA expression in HF and EAF, respectively. **d** Heat map showing differentially expressed lncRNAs (DELs) between the HF and EAF groups. **e** Validation of the DELs. The expression levels of the DELs in HF and EAF were detected by qRT-PCR. The data are presented as the mean ± SEM of at last three independent experiments. * *P* < 0.05. ** *P* < 0.01. *** *P* < 0.001

A total of 10,066 transcripts and 1,918 lncRNAs were identified in follicles, 1,807 and 1,899 lncRNAs were detected in the HF and EAF groups, and 1,788 lncRNAs were found in both the HF and EAF groups (Fig. 1a, b, Table S1). The chromosomal distribution of follicular lncRNAs was not uniform, and the number of transcribed lncRNAs on chromosome 1 was the highest, while the number of those on chromosome 10 was the lowest (Fig. 1c). Notably, 77 DELs, including 67 upregulated and 10 downregulated lncRNAs, were identified in the EAF group, compared to the HF group (Fig. 1d, Table S2). Furthermore, qRT-PCR confirmed the accuracy of the RNA-Seq data (Fig. 1e). A total of 387 *cis*-target mRNAs of the DELs were identified (Table S3), and the GO analysis revealed that 17 significant GO terms were enriched, for example, angiogenesis, sequence-specific DNA binding, regulation of transcription from the RNA polymerase II promoter, and nucleus (Fig. S2, Table S4). In addition, KEGG pathway analysis revealed that multiple significant pathways were enriched, such as the MAPK signaling pathway, oxytocin signaling pathway, melanogenesis, and insulin secretion (Fig. S3, Table S5).

### NORHA is a novel cytoplasmic lncRNA involved in follicular atresia

**Fig. 2:**
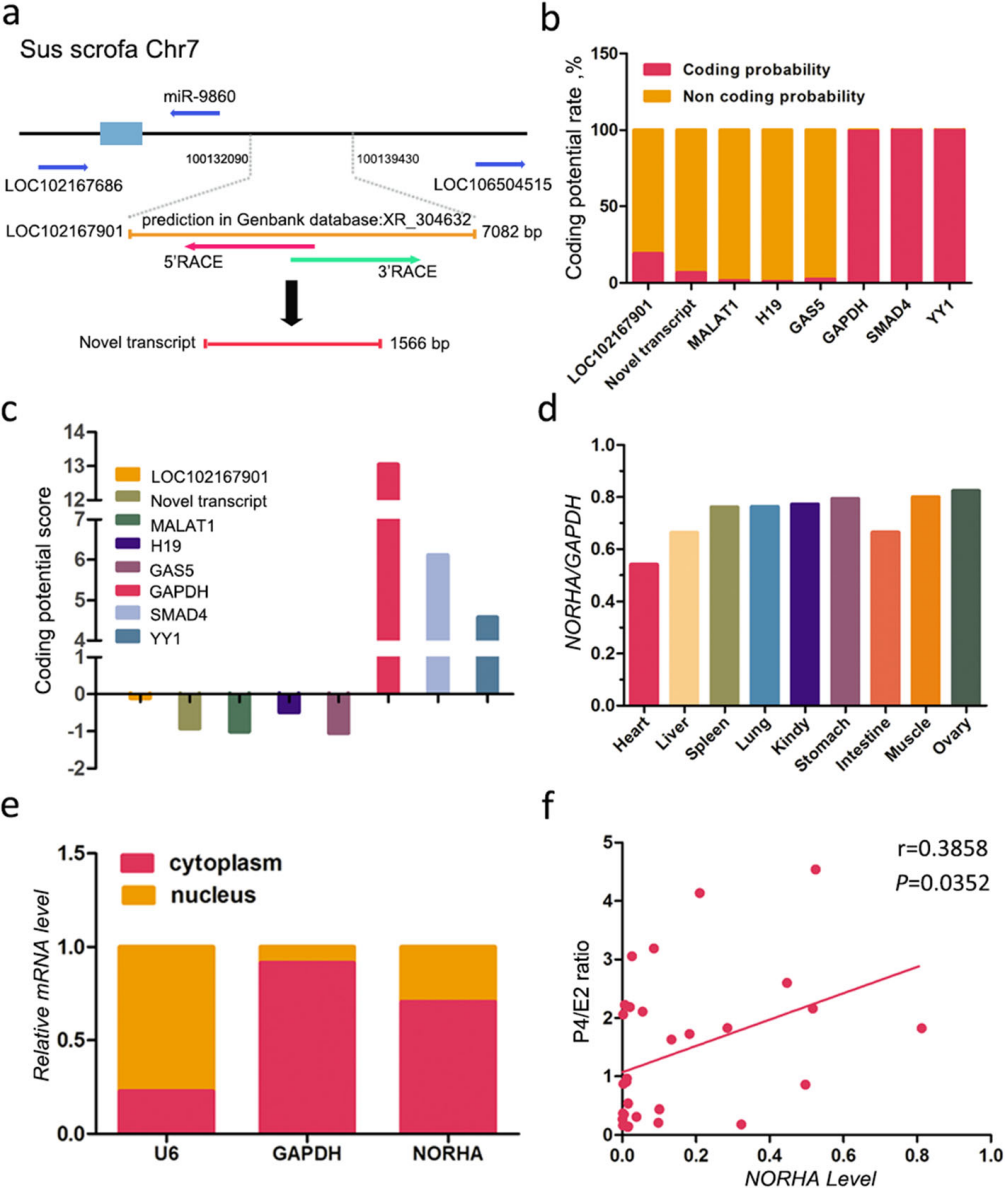
NORHA is a novel cytoplasmic lncRNA involved in follicular atresia in pigs. **a** Flow diagram showing the location and predicted length (XR_304632, 7,082 bp, yellow line) of LOC102167901 in the GenBank database. According to reference sequences, we designed a special primer to isolate full-length LOC102167901 (a novel lncRNA, 1,566 bp, shown by the red line) through a RACE assay (Fig. S4**a**). **b, c** The coding potential of novel transcripts was predicted by the CPAT (b) and CPC (c) methods. LOC102167901 is the sequence obtained from the NCBI database. MALAT1, H19 and GAS5 are well-known lncRNAs, and GAPDH, SMAD4 and YY1 are protein-coding genes. **d** Tissue expression profile of NORHA. **e** Subcellular distribution of NORHA in GCs. The expression levels of NORHA, GAPDH and U6 (reference genes) in the nucleus (NORHA and U6) and cytoplasm (NORHA and GAPDH) were quantified by qRT-PCR. **f** The correlation of follicular NORHA levels and the P4/E2 ratio in follicular fluid

LOC102167901 was the most significantly elevated among the seven verified DELs and was chosen for further investigation. We isolated the full-length RNA sequence of the porcine LOC102167901 (a novel transcript) of 1,566 bp (Figs. 2a and S4a, b), which differed from the original sequences of LOC102167901 documented in the GenBank database (XR_304632, 7,082 bp, predicted). The protein-coding ability scores of the LOC102167901-derived novel transcript were 0.067 (CPAT method) and − 0.92330 (CPC method). These scores were close to those of other known lncRNAs (e.g., MALAT1 and H19) (Fig. 2b, c), indicating that the novel transcript is devoid of protein-coding potential and that it is a true lncRNA. The novel lncRNA was named: a noncoding RNA that was highly expressed in atretic follicles - NORHA.

NORHA is a sense lncRNA located in a region from 100,135,521 nt to 100,137,345 nt on pig chromosome 7 (Fig. 2a). The tissue expression pattern revealed that the highest expression of NORHA was found in the ovary and the lowest expression in the heart (Figs. 2d and S5). The subcellular location showed that NORHA was enriched in the cytoplasm of porcine GCs (Fig. 2e). In ovarian follicles, NORHA expression levels were positively correlated with the P4/E2 ratio (r = 0.3858, *P* < 0.05), a biomarker of follicular atresia (Fig. 2f).

### NORHA induced GC apoptosis

**Fig. 3:**
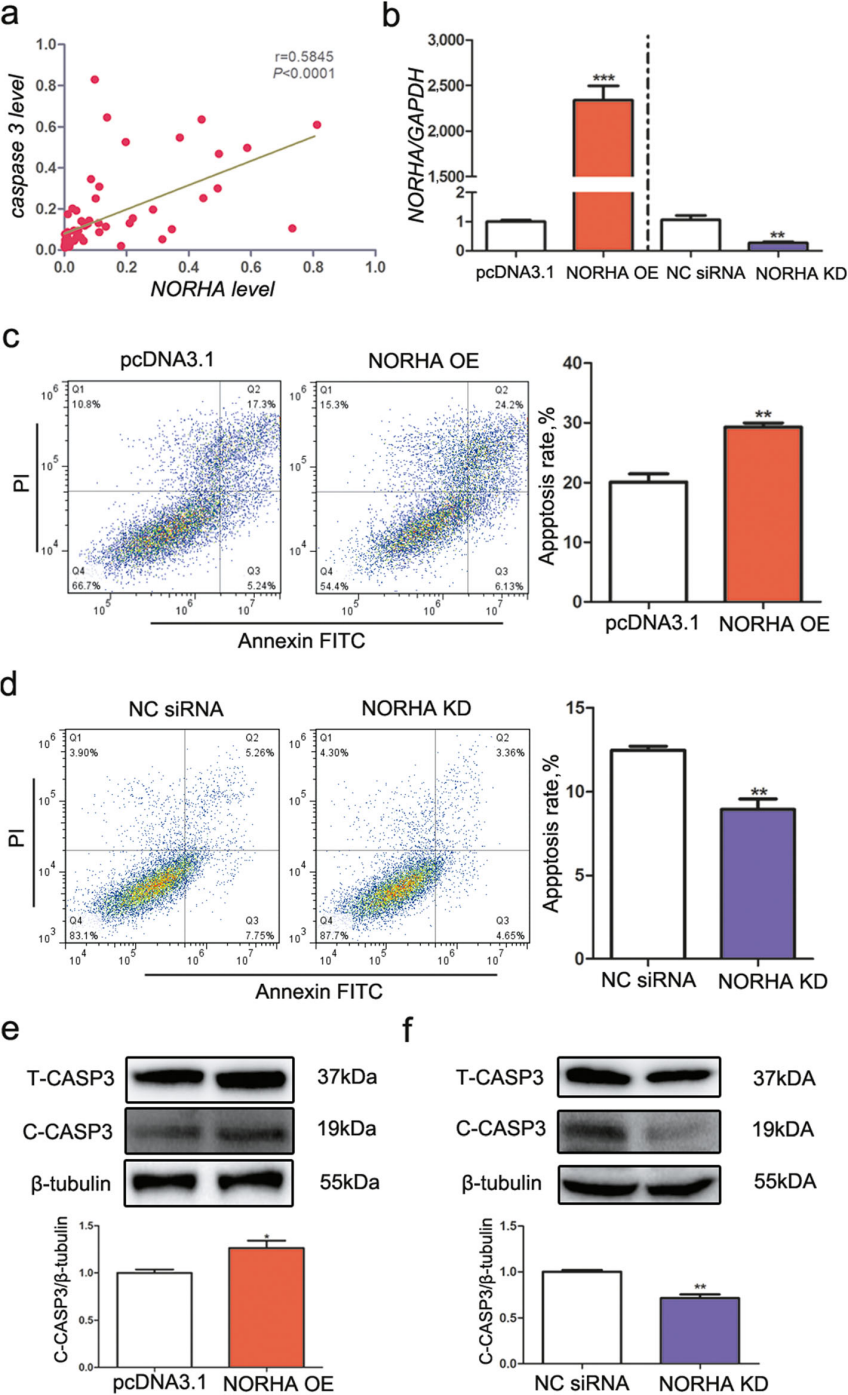
NORHA induces cell apoptosis of porcine GCs. **a** The mRNA expression levels of NORHA and caspase 3 are positively correlated in follicles. **b** Overexpression and knockdown of NORHA in GCs by transfection with pcDNA3.1-NORHA and NORHA-siRNA, respectively. NORHA levels were detected by RT-PCR and normalized to those of GAPDH. **c, d** NORHA controls GC apoptosis. GCs were treated with pcDNA3.1-NORHA (c) and NORHA-siRNA (d), and cell apoptosis was detected by flow cytometry. **e, f** NORHA controls Caspase 3 expression in GCs. GCs were treated with pcDNA3.1-NORHA (e) and NORHA-siRNA (f), and the protein levels of total Caspase 3 (T-CASP3) and cleaved Caspase 3 (C-CASP3) were measured by western blotting. The data are presented as the mean ± SEM of at least three independent experiments. * *P* < 0.05. ** *P* < 0.01. *** *P* < 0.001

Follicular NORHA levels were positively correlated with the mRNA levels of caspase 3, a proapoptotic marker of porcine GC apoptosis (r = 0.5845, *P* < 0.01) (Fig. 3a). Furthermore, overexpression of NORHA increased the apoptosis rate of GCs (Fig. 3b, c), whereas silencing of NORHA decreased the apoptosis rate of GCs (Fig. 3b, d). In addition, cleaved Caspase 3 (C-CASP3) protein levels were increased in GCs after NORHA was overexpressed (Fig. 3e) but decreased in GCs after NORHA was knocked down (Fig. 3f).

### NORHA was a molecular sponge of the miR-183-96-182 cluster

**Fig. 4:**
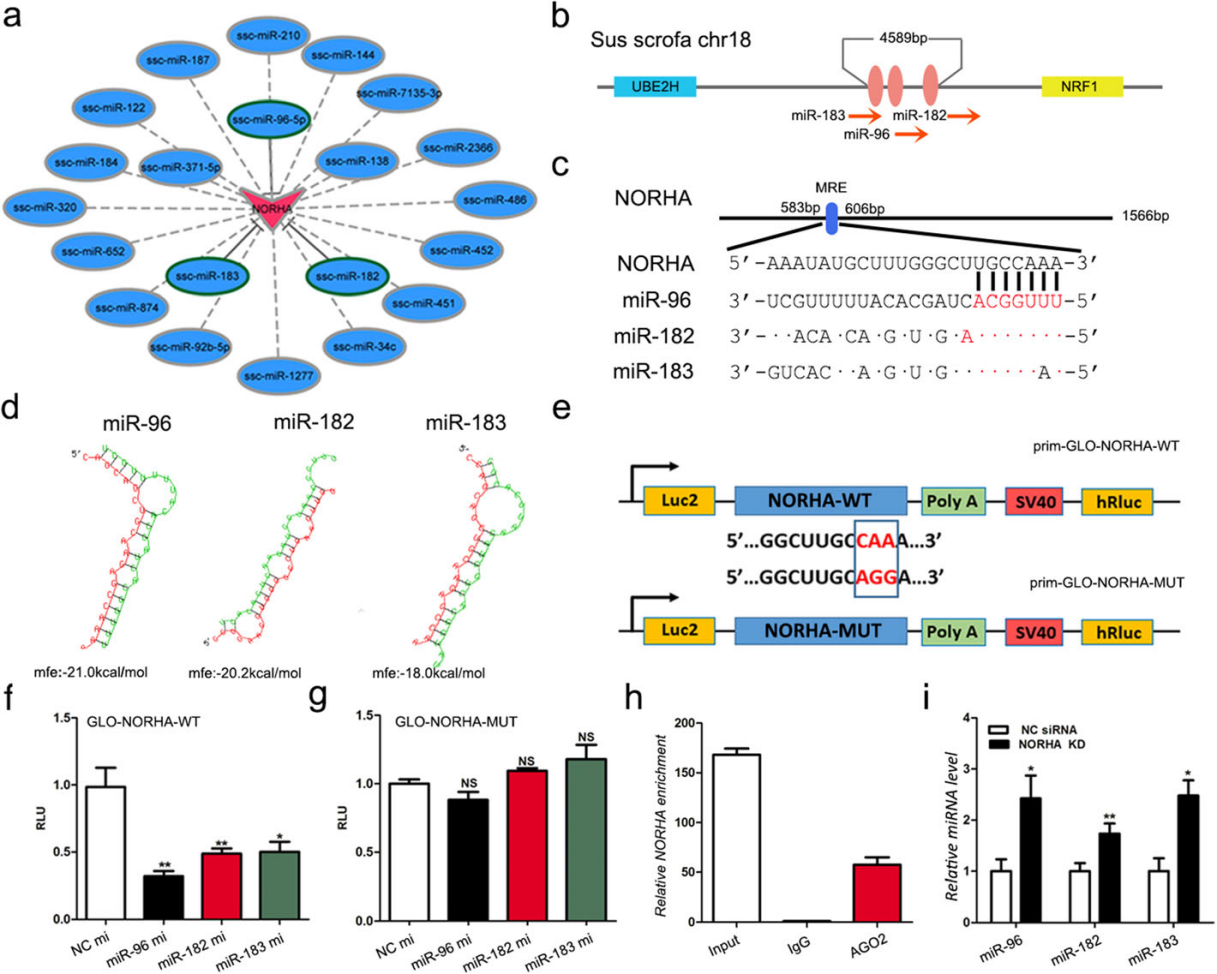
NORHA is a molecular sponge of the miR-183-96-182 cluster. **a** An interaction network between NORHA and miRNAs that were downregulated in EAF. The data of miRNA-Seq are not shown. The red triangle represents NORHA, which was upregulated in EAF, and blue ellipses represent the miRNAs that were downregulated in EAF. **b** Chromosomal localization of miR-183, miR-96, and miR-182 show that they form a miRNA cluster in a common genome region. **c** Schematic showing the interactions of miR-183, miR-96, and miR-182 seed sequences with the NORHA RNA sequence. **d** Minimum free energy (MFE) of miR-183, miR-96, and miR-182 binding to NORHA was predicted by RNAhybrid. **e** Schematic showing the reporter constructs of NORHA. The NORHA sequence containing wild-type and mutant miRNA-binding sites was amplified and inserted into a prim-GLO plasmid to construct a luciferase reporter vector. **f, g** Wild-type (f) or mutant (g) reporter vectors were cotransfected with miR-183 mimics (mi), miR-96 mimics (mi), and miR-182 mimics (mi) into HEK293T cells, and luciferase activity was measured. **h** RIP assay was performed with an anti- AGO2-specific antibody. The levels of NORHA enriched on the AGO2 protein were detected by qRT-PCR. IgG antibody was used as a negative control. **i** The levels of miR-183, miR-96 and miR-182 in GCs with NORHA knocked down. The data are presented as the mean ± SEM of at last three independent experiments. * *P* < 0.05. ** *P* < 0.01. ns, not significant

The interaction network between cytoplasmic NORHA and miRNAs (Fig. 4a) showed that NORHA is a potential competing endogenous RNA (ceRNA) of 21 miRNAs that are downregulated in the EAF group (the details on the results of the miRNA profiles of the HF and EAF groups are not shown). Interestingly, the miR-183-96-182 cluster contains transcripts from a common genomic region (nt 18,982,506 – nt 18,982,590) on pig chromosome 18 (Figs. 4b and S6) and were predicted through RNAhybrid to bind to NORHA (Fig. 4c, d). Next, we generated a dual-luciferase reporter vector harboring the response element of the miR-183-96-182 cluster (Fig. 4e). Luciferase assays revealed that miR-183, miR-96, or miR-182 significantly decreased the luciferase activity of the reporter vector in HEK293T cells (Fig. 4f). However, the three miRNAs had no effect on the luciferase activity of the reporter construct with a mutated binding site (Fig. 4g). The RIP assay showed that NORHA was enriched with AGO2, a member of the RISC (RNA-induced silencing complex) family (Fig. 4h). Furthermore, the miR-183-96-182 cluster was upregulated in NORHA-silenced GCs (Fig. 4i).

### The miR-183-96-182 cluster inhibited GC apoptosis

**Fig. 5:**
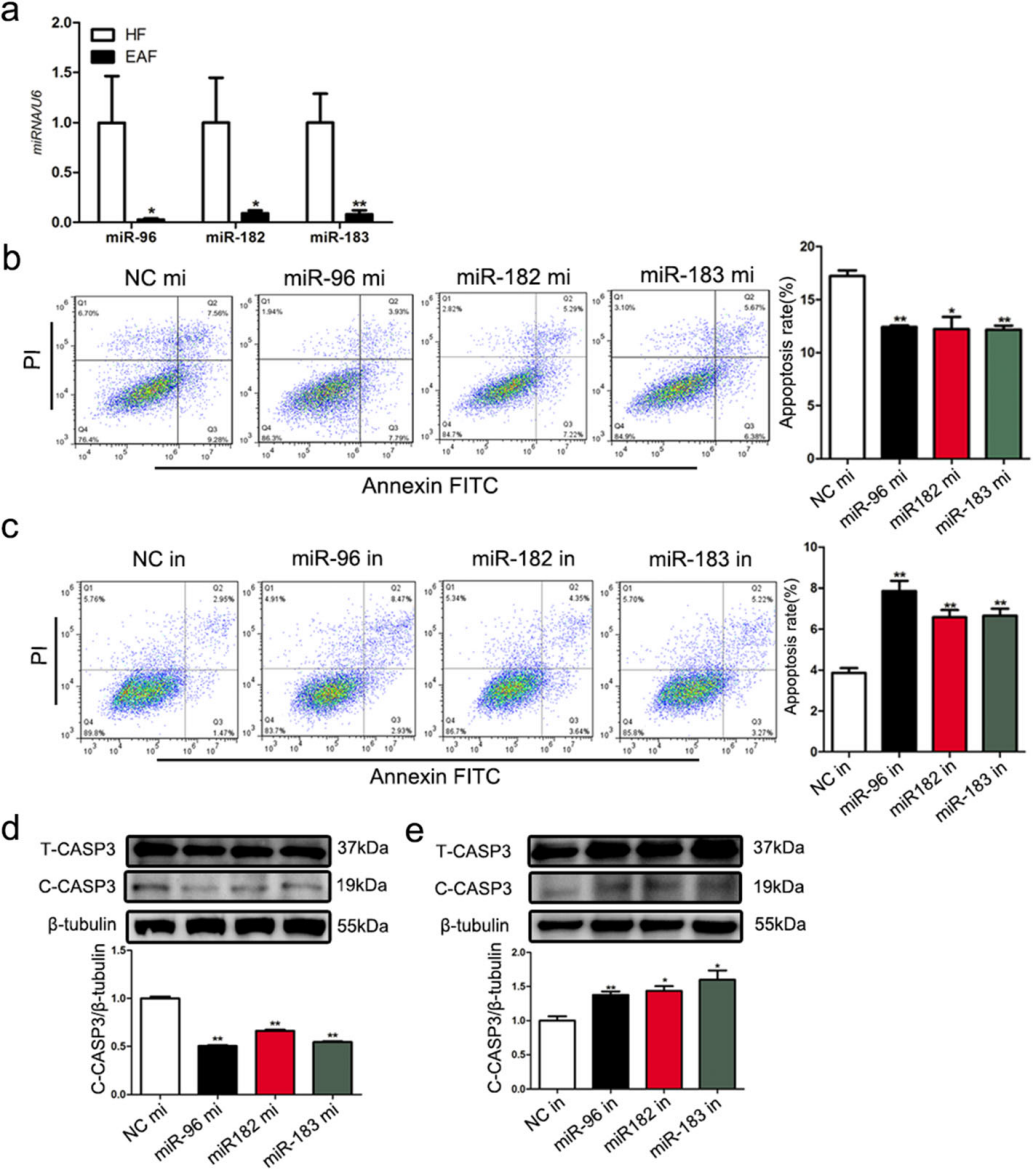
The miR-183-96-182 cluster inhibits GC apoptosis. **a** The expression of the miR-183-96-182 cluster in HF and EAF was detected by RNA-Seq. **b, c** The miR-183-96-182 cluster controls GC apoptosis. GCs were transfected with mimics (mi) (c) or inhibitors (in) (d) of miR-183, miR-96 and miR-182, and the apoptosis rate was determined by flow cytometry. **d, e** The miR-183-96-182 cluster controls Caspase 3 expression in GCs. GCs were transfected with mimics (mi) (e) or inhibitors (in) (f) of miR-183, miR-96 and miR-182, and the protein levels of T-CASP3 and C-CASP3 were detected by western blot. The data are presented as the mean ± SEM. of at last three independent experiments. * *P* < 0.05. ** *P* < 0.01. *** *P* < 0.001

We showed that the levels of three members of the miR-183-96-182 cluster were decreased in the porcine EAF group compared to the HF group (Fig. 5a). Furthermore, overexpression of the miR-183-96-182 cluster obviously decreased the percentage of apoptotic cells (Fig. 5b) and markedly suppressed the levels of C-CASP3 (Fig. 5d). In contrast, the percentage of apoptotic cells (Fig. 5c) and C-CASP3 levels (Fig. 5e) were significantly increased in the miR-183-96-182 cluster-silenced GCs.

### FoxO1 was a common target of the miR-183-96-182 cluster

**Fig. 6:**
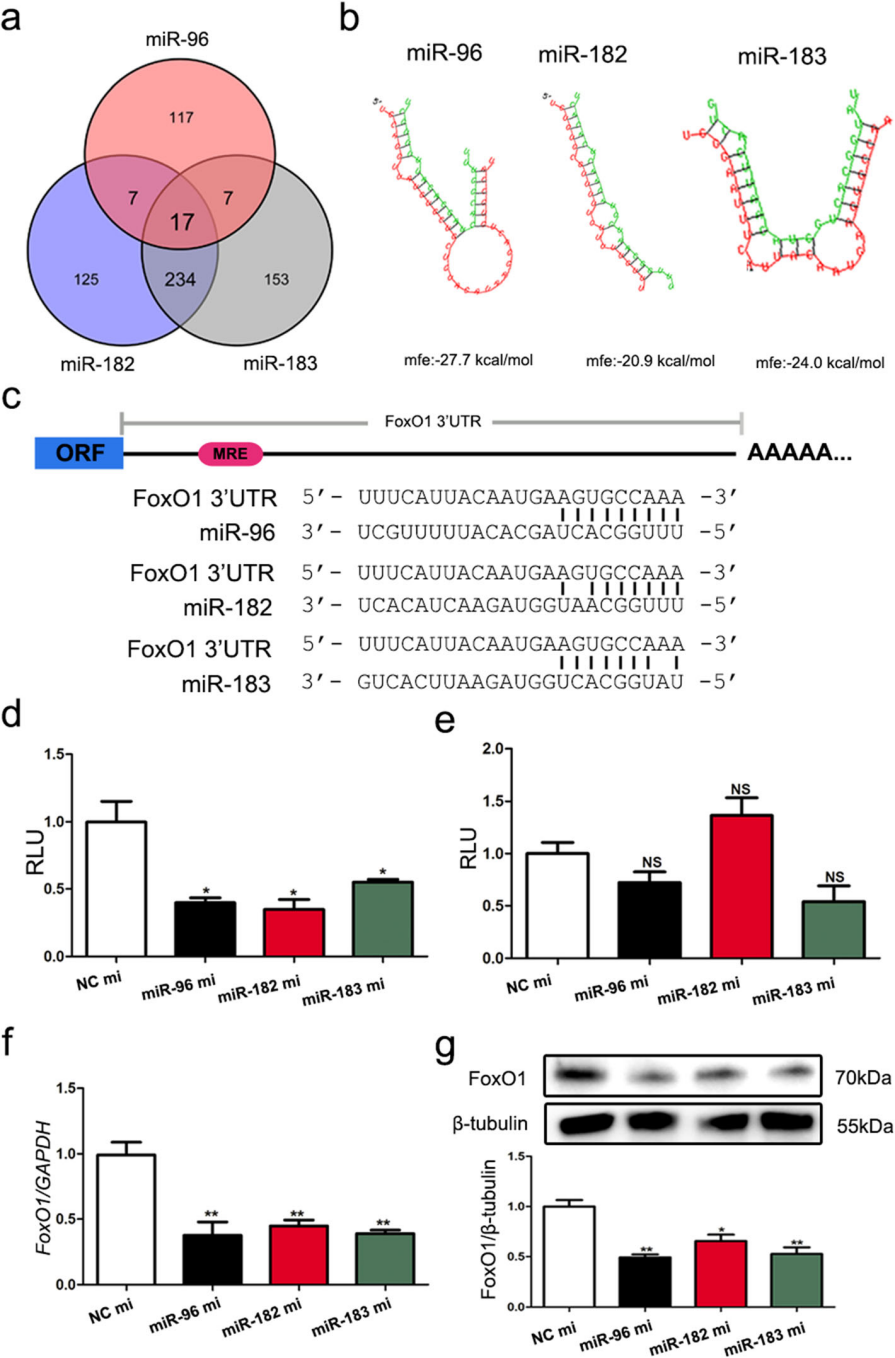
FoxO1 is a common target of the miR-183-96-182 cluster in GCs. **a** The common putative targets of the miR-183-96-182 cluster. The putative targets of miR-183, miR-96, and miR-182 were predicted by five programs: TargetScan, Pictar2, PITA, RNA22, and RNAhybrid. **b** The MFE of the miR-183-96-182 cluster binding to the 3′-UTR of the porcine FoxO1 gene was predicted by RNAhybrid. **c** The binding sites of the miR-183-96-182 cluster in the 3′-UTR of the porcine FoxO1 gene. **d, e** Luciferase assay. Luciferase activity was measured in HEK293T cells cotransfected with mimics (mi) of miR-183, miR-96, or miR-182 and reporter vectors of FoxO1 3′-UTR harboring the wild-type (d) or mutated (e) miRNA-binding site. **f, g** GCs were transfected with mimics (mi) of miR-182, miR-96, or miR-183, and the mRNA (f) and protein (g) levels of FoxO1 were detected by qRT-PCR and western blotting, respectively. The data are presented as the mean ± SEM. of at last three independent experiments. * *P* < 0.05. ** *P* < 0.01. NS, not significant

A total of 148, 383, and 411 potential targets were predicted for miR-183, miR-96, and miR-182, respectively (Fig. S7a), and seventeen genes were commonly targeted by the miR-183-96-182 cluster (Fig. 6a). Of these genes, FoxO1, a core member of the forkhead box O (FoxO) family and regulator of GC apoptosis in mammals, was selected as a candidate target of the miR-183-96-182 cluster for further study. Furthermore, we predicted that FoxO1 had not only a high capacity for interaction with the miR-183-96-182 cluster (Fig. 6b) but also contained an MRE (miRNA response element) of the miR-183-96-182 cluster at 2,261 nt – 2,267 nt (GenBank ID: NM_214014) (Figs. 6c and S7b).

Next, we constructed a pmirGLO dual-luciferase reporter vector of the porcine FoxO1 3′-UTR containing the MRE motif (Fig. S8a). The luciferase activity of the porcine FoxO1 3′-UTR reporter vector was significantly attenuated in HEK293T cells treated with miR-183, miR-96 or miR-182 mimics (Fig. 6d), but the luciferase activity of the MRE-mutated reporter vector was not altered (Figs. 6e and S8b). In addition, the miR-183-96-182 cluster significantly inhibited FoxO1 expression in GCs at both the transcriptional and translational levels (Fig. 6f, g).

### NORHA induced FoxO1-mediated GC apoptosis by competing for the miR-183-96-182 cluster

**Fig. 7:**
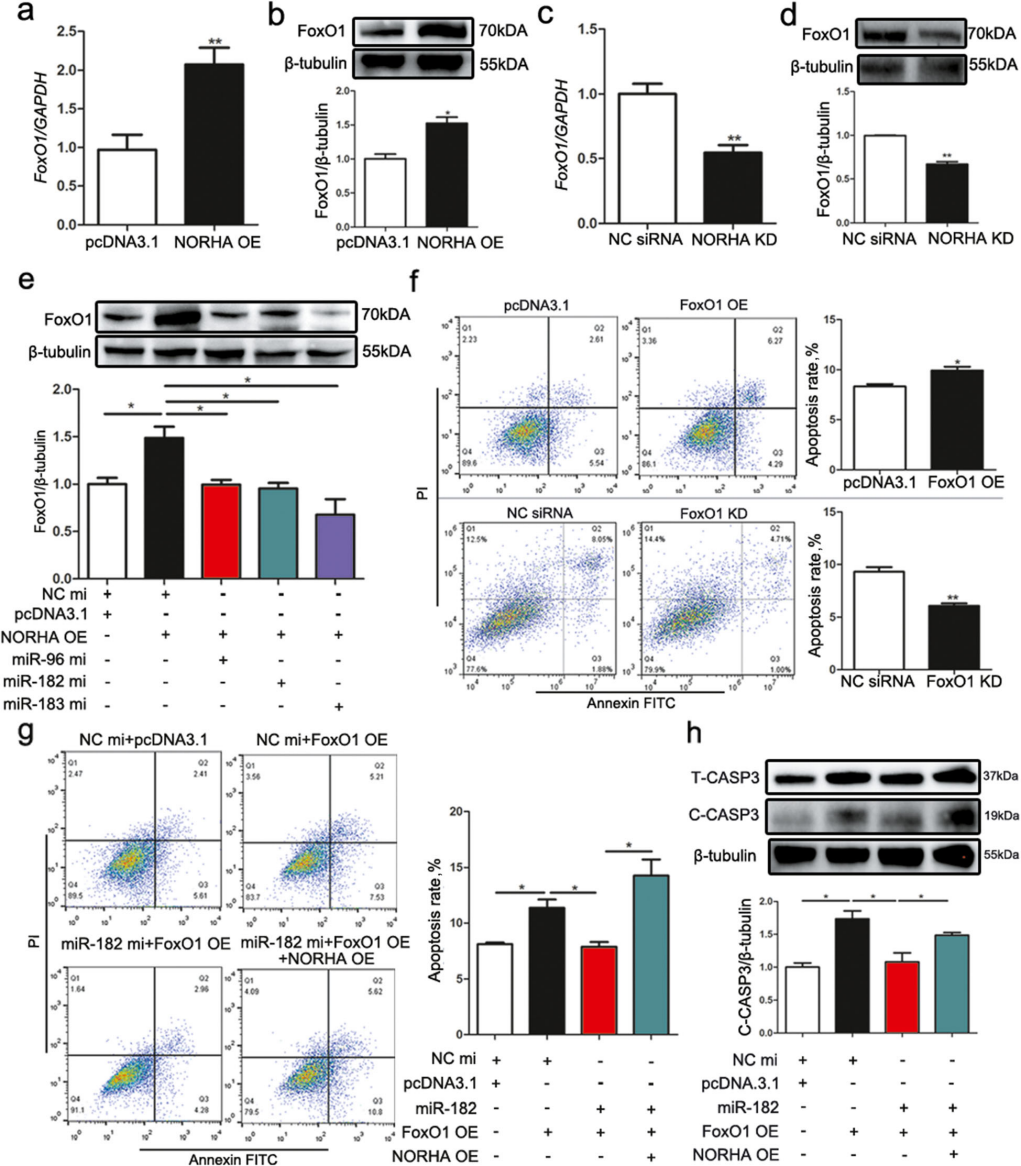
NORHA induces FoxO1-mediated GC apoptosis by competing for the miR-183-96-182 cluster. **a-d** NORHA induces FoxO1 expression. The mRNA (a) and protein (b) levels of FoxO1 were detected in porcine GCs transfected with pcDNA3.1-NORHA. The mRNA (c) and protein (d) levels of FoxO1 were detected in GCs transfected with NORHA-siRNA. **e** NORHA induces FoxO1 expression via the miR-183-96-182 cluster. GCs were cotransfected with mimics (mi) of miR-183, miR-96, or miR-182 and pcDNA3.1-NORHA, and the protein levels of FoxO1 were measured by western blot. **f** FoxO1 contributes to GC apoptosis. pcDNA3.1-FoxO1 (upper part) or FoxO1-siRNA (lower part) was transfected into GCs, and the apoptosis rate was measured by flow cytometry. **g, h** NORHA induces GC apoptosis via the miR-182/FoxO1 axis. GCs were cotransfected with pcDNA3.1-FoxO1, miR-182 mimics (mi) and pcDNA3.1-NORHA, and the apoptosis rate was determined (g), and protein levels of T-CASP3 and C-CASP3 (h) were detected. The data are presented as the mean ± SEM of at last three independent experiments. * *P* < 0.05. ** *P* < 0.01

Overexpression of NORHA expression by the plasmid pcDNA3.1-NORHA significantly induced FoxO1 expression in GCs (Fig. 7a, b). In contrast, knocking down NORHA significantly reduced FoxO1 expression in GCs (Fig. 7c, d). Furthermore, a cotransfection assay showed that the miR-183-96-182 cluster attenuated NORHA-induced FoxO1 expression in GCs (Fig. 7e).

Next, overexpression of FoxO1 induced GC apoptosis, whereas knockdown of FoxO1 reduced GC apoptosis, indicating that FoxO1 promoted porcine GC apoptosis (Fig. 7f). Furthermore, we showed that FoxO1-induced GC apoptosis was reversed by miRNA-182, and this process was also inhibited by NORHA (Fig. 7g, h).

### NORHA and oxidative stress synergistically induced GC apoptosis

**Fig. 8:**
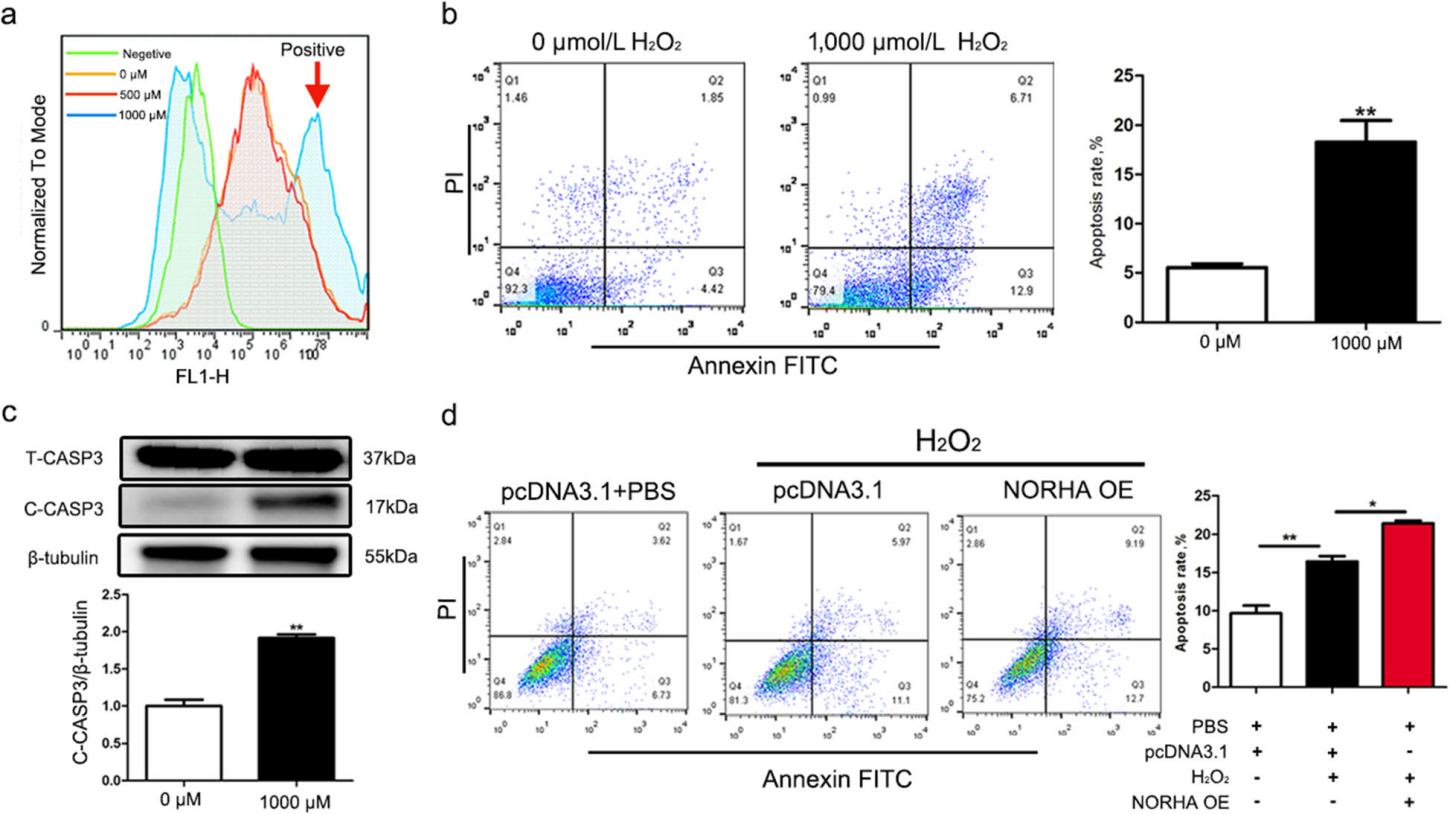
NORHA and oxidative stress synergistically induce GC apoptosis. **a** H_2_O_2_ induces oxidative stress. Porcine GCs were treated with H_2_O_2_ (0, 500, and 1,000 μmol/L) for 90 min, and reactive oxygen species (ROS) levels were detected by flow cytometry. **b, c** Oxidative stress induced by H_2_O_2_ induces GC apoptosis. The apoptosis rate (b) and protein levels of T-CASP3 and C-CASP3 (c) were measured in GCs treated with 1,000 μmol/L H_2_O_2_. **d** NORHA and oxidative stress synergistically induced GC apoptosis. GCs were treated with pcDNA3.1-NORHA with or without H_2_O_2_, and the apoptosis rate was calculated. The data are presented as the mean ± SEM of at last three independent experiments. * *P* < 0.05. ** *P* < 0.01

In porcine GCs, H_2_O_2_ induced oxidative stress, which led to a significant increase in ROS levels (Fig. 8a). In addition, the cell apoptosis rate and C-CASP3 levels were upregulated in GCs with continued H_2_O_2_ stimulation (Fig. 8b, c). Furthermore, overexpression of NORHA induced the apoptosis rate of the GCs exposed to H_2_O_2_ (Fig. 8d), revealing that NORHA and oxidative stress can synergistically induce GC apoptosis.

## Discussion

A large number of primordial follicles exist in the ovarian follicle pool in mammals: approximately 420,000 in pigs and 400,000 in humans. However, fewer than 1% of follicles mature and ovulate, while most follicles are atretic and degenerate [29]. Therefore, follicular atresia not only restricts the effective utilization of the primordial follicle pool but also limits the potential reproductive ability of domestic animals. Many factors related to follicular atresia have been identified including follicle-stimulating hormone (FSH), TGF-β and death receptor-mediated signaling pathways as well as liver receptor homolog-1 (LRH-1) and X-linked inhibitor of apoptosis (XIAP) [28, 30–34]. In recent years, high-throughput technology has gradually become an important way to fully understand the molecular events during follicular atresia, and the expression profile of miRNAs [27] and mRNAs [35] in follicular atresia has been revealed. Here, we constructed a lncRNA profile during follicular atresia, and multiple DELs were identified. Regulation of adjacent or host genes via *cis* regulation is a main functional model of lncRNA action [36, 37]. Notably, among the *cis*-target mRNAs of these DELs, multiple genes, such as CYP11A1 [38], BABAM2 [39], fibroblast growth factor 18 (FGF18) [40], and neurogenic locus notch homolog protein 2 (NOTCH2) [41], have been implicated in GC functions (e.g., steroid hormone synthesis), follicular atresia, ovarian functions, and female fertility. Our findings are the first to identify lncRNAs in follicular atresia and provide an understanding of the lncRNAs that are involved in follicular atresia. The ovarian follicle is known to be a complex multicellular structure that contains many cell types, such as GCs, theca cells and oocytes. Emerging data have demonstrated that the function of these cells and the levels of transcripts, including mRNAs and ncRNAs, change during follicular atresia [24, 42–46]. The deficiency of this study was that it was based only on the current understanding of the dynamics of DELs during follicular atresia at the level of the whole follicle but not the dynamics of lncRNAs in a single cell type, which may need to be tested by single-cell sequencing in the future.

We demonstrated that NORHA was highly involved in porcine follicular atresia by enhancing GC apoptosis. In GCs, lncRNAs have been reported to be associated with various cellular functions, such as cell proliferation, cell cycle progression [18, 22] and secretion of steroid hormones including E2, P4, and testosterone (T) [47, 48]. However, few studies have investigated the regulation of GC apoptosis by lncRNAs. A recent study showed that lncRNA steroid receptor RNA activator (SRA), an important player in transcriptional regulation, is thought to interact with a DNA-binding protein by binding to specific DNA sequences [49], which induces the release of E2 and P4 and reduces the apoptosis rate of mouse GCs [48]. Prader–Willi region nonprotein coding RNA 2 (PWRN2), a CC-expressed lncRNA, is thought to be associated with oocyte nuclear maturation by sponging miR-92b-3p in the human ovary [50]. In addition, some lncRNAs were reported to be involved in various cellular processes in ovarian cancer cells [51]. MLK7-AS1, for instance, a lncRNA that is specifically upregulated in ovarian cancer tissues, controls multiple cellular processes (e.g., stifles cell invasion, proliferation, and wound healing and promotes cell apoptosis), modulates the epithelial-mesenchymal transition (EMT) process by influencing the miR-375/Yes-associated protein 1 (YAP1) axis [51]. Taken together, our findings are the first to identify and characterize lncRNAs associated with follicular atresia and provide evidence that NORHA can serve as a potential diagnostic biomarker and rescue target for follicular atresia.

As an important regulatory RNA, lncRNAs exert their biological functions mainly by regulating target expression at various levels, from transcription to protein localization and stability [6]. The subcellular localization (cytoplasm and/or nucleus) of lncRNAs is the principal determinant of their molecular function and mode of action [52]. The most common mechanism of action of both cytoplasmic and nuclear lncRNAs is, as a ceRNA, regulating target expression via a lncRNA-miRNA-target axis [9, 52, 53]. For instance, lncRNA-protein phosphatase 1 nuclear-targeting subunit (PNUTS), a noncoding isoform of the protein-coding gene PNUTS, is a ceRNA of miR-205 that influences EMT-related cell migration and invasion by controlling the miR-205/ZEB/E-cadherin axis [54]. More recently, temozolomide-associated lncRNA (TALC), a highly expressed lncRNA in temozolomide-resistant glioblastoma, has been demonstrated to function as a ceRNA to competitively bind miR-20b-3p to enhance c-Met expression [53]. TALC then activates the Stat3/p300 complex to increase the transcriptional activity of the O^6^-methylguanine-DNA methyltransferase by modulating the acetylation of H3, including H3K9, H3K27, and H3K36 [53]. Here, we demonstrated that NORHA functioned as a ceRNA for the miR-183-96-182 cluster, relieving its inhibition of the target FoxO1 and promoting cell apoptosis in porcine GCs.

The miR-183-96-182 cluster is a polycistronic miRNA cluster that is located within a 5-kb genomic region on chromosome 7 in humans, chromosome 6 in mice, and chromosome 18 in pigs. Importantly, this family is not only highly conserved among different species but also has seed sequences that are similar among different members of the same species, implying that the members of this cluster may share the same targets and biological functions [55, 56]. Consistent with this hypothesis, recent reports have shown that the common targets of the miR-183-96-182 cluster are HDAC9 which encode a histone, which encodes a histone deacetylase that influences memory formation [57]; Cacna2d2, which encodes the auxiliary voltage-gated calcium channel subunit α2δs to scale mechanical pain sensitivity [55]; and DAP12 and Nox2, which control macrophage functions in response to *P. aeruginosa* infection [58]. In addition, increasing the miR-183-96-182 cluster in luteal tissues relative to follicular tissues can enhance cell survival and P4 release by luteal cells in both humans and cattle by targeting FoxO1, respectively [59]. Herein, we demonstrated that the miR-183-96-182 cluster inhibited the common target FoxO1 and mediated NORHA regulation of porcine GC apoptosis. In the ovary, the miR-183-96-182 cluster was mainly expressed in both follicular GCs and the corpus luteum, playing a vital role in cell proliferation and the cell cycle of bovine GCs [60]. Together, our results indicate that the miR-183-96-182 cluster influences follicular atresia by repressing GC apoptosis .

Determination of the mechanism of the miR-183-96-182 cluster repression of GC apoptosis led us to identify FoxO1 as a functional target of the miR-183-96-182 cluster, showing that FoxO1 mediates the antiapoptotic function of the miR-183-96-182 cluster in GCs. FoxO1, a member of the FOXO family, has been shown to be a direct target of dozens of miRNAs, including the miR-183-96-182 cluster in mammals [60, 63]. As a functional target of the miR-183-96-182 cluster, FoxO1 participates in the miR-183-96-182 cluster regulation of multiple functions in various cells and tissues, such as cell death in endometrial cancer [61], pathogenicity in Th17 cells [56], adipogenesis in C2C12 myoblasts [62], and sperm quality in mouse testes [63]. Notably, in ovarian GCs, FoxO1 also mediates the miR-183-96-182 cluster regulation of GC functions [60]. In bovine GCs, for instance, FoxO1 is inhibited by the miR-183-96-182 cluster and can reduce the proportion of cells in S phase [60]. In addition, FoxO1 is thought to be associated with other GC functions, such as autophagy [30], apoptosis [64], proliferation [60] and differentiation [65], and response to FSH [66].

## Conclusion

Collectively, our results show alterations in lncRNA expression in porcine HF and EAF and identified a novel lncRNA, NORHA, which was highly expressed in atretic follicles. NORHA induced follicular atresia and GC apoptosis via a miR-183-96-182 cluster/FoxO1 axis by competitively sponging the miR-183-96-182 cluster (Fig. S9). Our findings reveal new epigenetic mechanisms of follicular atresia and GC apoptosis, providing evidence that NORHA can serve as a potential diagnostic biomarker and rescue target for follicular atresia, as well as a novel candidate for the improvement of female fertility.

## Supplementary Information

**Additional file 1: Table S1.** LncRNAs identified in the porcine ovarian follicles. **Table S2.** DELs in HF vs EAF. **Table S3.** cis-target mRNAs of DELs. **Table S4.** GO terms for DELs. **Table S5.** KEGG pathway analysis for DELs. **Table S6.** Primers used in this study. **Table S7.** Oligonucleotides used in this study.

**Additional file 2: Figure S1.** Overview of experiment design. Flow diagram showing the experimental design and methods. The intermittent blue arrow represents the indirect regulation between NORHA and FoxO1. **Figure S2.** Gene ontology (GO) analysis. GO functional enrichment of the *cis*-target mRNAs for all DELs was performed by an online tool DAVID (https://david-d.ncifcrf.gov/). The number of the enriched genes in each GO term was depicted over bars. * *P* < 0.05. ** *P* < 0.01. **Figure S3.** Kyoto encyclopedia of genes and genome (KEGG) pathway analysis. The significant pathways were shown in the bubble chart generated by R software using the *cis*-target mRNAs of DELs. The size of each bubble indicates the number of genes in each pathway. The color of each bubble represents enrichment *P* value. **Figure S4.** The full-length of LOC102167901 was obtained by rapid amplification of cDNA ends (RACE). **a** Nested PCR amplified product obtained from 5′-RACE and 3′ RACE assay was found to have 1021 bp (lane 2) and 1000 bp (lane 1), respectively. DNA marker DL2000 is shown in lane M**. b** The full-length sequence of the novel transcript. **Figure S5.** The expression prolife pattern of the porcine NORHA. The expression of NORHA in the heart, liver, spleen, lung, kidney, stomach, intestine, muscle and ovary was detected. GAPDH acts as an internal control. M, DNA marker DL2000. **Figure S6.** Alignments of mature sequences of the miR-183-96-182 cluster in vertebrates. The seed region (nucleotides 2–8) was shown in the red box. Ssc, *S. scrofa*; hsa, *H. sapiens*; mmu, *M. musculus*; bta, *B. taurus*; xtr, *X. tropicalis*; bfl, *branchiostoma floridae*; ggo, *G. gorilla*; ptr, *P. troglodytes*; gga, *G. gallus*. **Figure S6.** Alignments of mature sequences of the miR-183-96-182 cluster in vertebrates. The seed region (nucleotides 2–8) was shown in the red box. Ssc, *S. scrofa*; hsa, *H. sapiens*; mmu, *M. musculus*; bta, *B. taurus*; xtr, *X. tropicalis*; bfl, *branchiostoma floridae*; ggo, *G. gorilla*; ptr, *P. troglodytes*; gga, *G. gallus*. **Figure S7.** FoxO1 is a common candidate target of the miR-183-96-182 cluster. **a** The Venn diagram showing the potential targets of miR-183, miR-96, and miR-182. The potential targets of miR-183, miR-96, and miR-182 were predicted by using five online tools Targetscan, Pictar2, PITA, RNA22, and RNAhybrid, respectively. **b** Sequence alignment of binding-site of the miR-183-96-182 cluster within the 3′-UTR of FoxO1 gene from humans, pigs, and mice. The red box region indicates the seed region. **Figure S8.** Construction of FoxO1 3′-UTR reporter vector. **a** Schematic illustration shows that the primGLO reporter vector of FoxO1 3′-UTR containing the binding site of the miR-183-96-182 cluster. **b** Reporter vector of FoxO1 3′-UTR containing wild type and mutant type binding site of the miR-183-96-182 cluster. **Figure S9.** The regulatory model of NORHA, miR-183-96-182 cluster and FoxO1 in healthy follicles and early atretic follicles. In early atretic follicles, levels of NORHA and oxidative stress are increased; up-regulated NORHA induces FoxO1 (an effector of oxidative stress) by acting as a sponge of the miR-183-96-182 cluster (the cluster inhibits FoxO1), and then NORHA, synergistically with FoxO1, induces apoptosis of granulosa cells.

## Data Availability

Not applicable.

## Abbreviations

lncRNA: Long noncoding RNA
NORHA: Non-coding RNA that was highly expressed in atretic follicles
CC: Cumulus cells
MREs: miRNA response elements
ceRNA: Competing endogenous RNA
FoxO1: Forkhead box O1
TGF-β: Transforming growth factor-β
SMAD4: Sma-and mad-related protein 4
NEAT1: Nuclear enriched abundant transcript 1
PCOS: Polycystic ovary syndrome
POI: Premature ovarian insufficiency
P4: Progesterone
E2: 17β-estradiol
HF: Healthy follicles
EAF: Early atretic follicles
GO: Gene ontology
KEGG: Kyoto encyclopedia of genes and genome
T-CASP3: Total Caspase 3
C-CASP3: Cleave Caspase 3
CPAT: Coding-potential assessment tool
CPC: Coding potential calculator
ROS: Reactive Oxygen Species
FSH: Follicle-stimulating hormone
LRH-1: Liver receptor homolog-1
XIAP: X-linked inhibitor of apoptosis
FSHR: Follicle stimulating hormone receptor
BMP: Bone morphogenetic protein
YAP1: Yes-associated protein 1
NOTCH2: Neurogenic locus notch homolog protein 2
PNUTS: Protein phosphatase 1 nuclear-targeting subunit
TALC: Temozolomide-associated lncRNA
TMZ: Temozolomide
STAT5: Signal transducer and activator of transcription 5
FOXP3: Forkhead box P3;
MFE: Minimum Free Energy
FACS: Fluorescence activated Cell Sorting
MRE: miRNA response element
