# Supplementary material for: NORHA, a novel follicular atresia-related lncRNA, promotes porcine granulosa cell apoptosis via the miR-183-96-182 cluster and FoxO1 axis
Source: J Anim Sci Biotechnol. 2021 Oct 7;12:103. doi: 10.1186/s40104-021-00626-7 (PMC8495971; doi:10.1186/s40104-021-00626-7)
Supplement: Supplementary file 1 — Additional file 1: Table S1. LncRNAs identified in the porcine ovarian follicles. Table S2. DELs in HF vs EAF. Table S3. cis-target mRNAs of DELs. Table S4. GO terms for DELs. Table S5. KEGG pathway analysis for DELs. Table S6. Primers used in this study. Table S7. Oligonucleotides used in this study. [file 40104_2021_626_MOESM1_ESM.docx]

**Table S1: LncRNAs identified in the porcine ovarian follicles**

| Transcript | Chr. | Strand | Start | Stop | Length | HF_count | EAF_count |
| --- | --- | --- | --- | --- | --- | --- | --- |
| XR_115968.2 | 13 | + | 41235879 | 41250359 | 1870 | 484 | 412 |
| XR_304346.1 | 13 | + | 24603720 | 24604965 | 541 | 2067 | 3794 |
| XR_301273.1 | 1 | - | 223940613 | 223969101 | 1153 | 59 | 54 |
| XR_301274.1 | 1 | - | 223940613 | 223969101 | 1110 | 59 | 52 |
| XR_301275.1 | 1 | - | 223940613 | 223969101 | 1188 | 61 | 55 |
| XR_301276.1 | 1 | - | 223940613 | 223969101 | 1033 | 57 | 54 |
| XR_309414.1 | 6 | + | 26585 | 27068 | 308 | 129 | 128 |
| XR_298275.1 | 7 | - | 144717807 | 144720290 | 1660 | 50 | 32 |
| XR_299489.1 | 7 | - | 40816694 | 40819444 | 629 | 10 | 12 |
| XR_303677.1 | 7 | + | 905639 | 906426 | 619 | 225 | 499 |
| XM_005653542.1 | 12 | + | 130993021 | 131000259 | 1542 | 2 | 5 |
| XR_301539.1 | 12 | - | 742598 | 747099 | 1405 | 1177 | 601 |
| XM_005666455.1 | 3 | + | 129704281 | 129747676 | 1208 | 52 | 49 |
| XR_304693.1 | 2 | + | 129704281 | 129747676 | 1533 | 53 | 78 |
| XR_302088.1 | 10 | - | 7907404 | 7910434 | 2960 | 82 | 94 |
| XR_305820.1 | 17 | + | 21604259 | 21611782 | 1277 | 104 | 234 |
| XR_305821.1 | 7 | + | 21604259 | 21611782 | 1228 | 115 | 206 |
| XR_308324.1 | 7 | - | 155386805 | 155418867 | 580 | 13 | 50 |
| XR_306603.1 | 7 | + | 54722634 | 54725540 | 322 | 190 | 128 |
| XR_307995.1 | X | - | 44734203 | 44739798 | 2169 | 95 | 146 |
| XR_302295.1 | 7 | - | 49674255 | 49685924 | 945 | 123 | 117 |
| XR_302970.1 | 7 | - | 76022772 | 76025166 | 1823 | 38 | 73 |
| XR_299098.1 | 7 | + | 558069 | 564872 | 287 | 0 | 2 |
| XR_308506.1 | 8 | + | 60057370 | 60077418 | 1915 | 87 | 100 |
| XR_304339.1 | 10 | - | 24386918 | 24406858 | 616 | 41 | 22 |
| XR_304340.1 | 2 | - | 24386918 | 24406858 | 545 | 40 | 15 |
| XR_304343.1 | 13 | - | 24421239 | 24423677 | 1429 | 562 | 466 |
| XM_005667979.1 | 13 | + | 22907140 | 22984164 | 1373 | 5 | 37 |
| XM_005655401.1 | 18 | + | 111081987 | 111110340 | 1760 | 125 | 133 |
| XR_304968.1 | 5 | - | 83252802 | 83259702 | 3121 | 88 | 91 |
| XR_305829.1 | 5 | + | 23706884 | 23713739 | 513 | 0 | 2 |
| XR_309268.1 | 15 | - | 109629627 | 109630699 | 372 | 78 | 43 |
| XR_303380.1 | X | + | 9939403 | 9957355 | 555 | 1 | 1 |
| XR_297291.1 | 9 | + | 160399099 | 160403206 | 430 | 8 | 6 |
| XM_005668395.1 | 9 | + | 16649549 | 16678321 | 1328 | 28 | 32 |
| XR_309232.1 | 6 | - | 67151878 | 67179369 | 19886 | 154303 | 96929 |
| XR_309233.1 | 9 | - | 67151878 | 67179369 | 8328 | 74560 | 47761 |
| XR_309234.1 | 9 | - | 67151878 | 67179369 | 9802 | 81851 | 52230 |
| XR_309235.1 | 9 | - | 67151878 | 67179369 | 8216 | 74066 | 47390 |
| XR_303384.1 | X | + | 10532608 | 10568718 | 1150 | 7 | 15 |
| XR_301580.1 | X | + | 6155870 | 6158222 | 1271 | 188 | 81 |
| XR_309078.1 | 13 | + | 47297161 | 47299212 | 302 | 21 | 11 |
| XR_305300.1 | 13 | + | 36135604 | 36157518 | 1290 | 583 | 516 |
| XR_305301.1 | 13 | + | 36135604 | 36157518 | 234 | 73 | 68 |
| XR_304323.1 | 3 | - | 21302932 | 21305478 | 276 | 9 | 1 |
| XR_300731.1 | 3 | - | 58700765 | 58774014 | 2056 | 2 | 26 |
| XR_303340.1 | 1 | - | 3624805 | 3632627 | 1260 | 3727 | 2436 |
| XR_306383.1 | 1 | + | 754739 | 757092 | 2067 | 121 | 115 |
| XR_299020.1 | 7 | + | 71362253 | 71365205 | 1665 | 95 | 87 |
| XR_297771.1 | 2 | + | 152168624 | 152190217 | 1826 | 2162 | 1255 |
| XR_303273.1 | 18 | + | 136877943 | 136893904 | 3719 | 9820 | 12828 |
| XR_303274.1 | 18 | + | 136877943 | 136893904 | 3286 | 8040 | 10722 |
| XR_303275.1 | 14 | + | 136877943 | 136893904 | 3704 | 9572 | 12551 |
| XR_304689.1 | 2 | - | 129247351 | 129258960 | 799 | 17 | 24 |
| XR_304690.1 | 7 | - | 129247351 | 129258960 | 790 | 17 | 24 |
| XR_299825.1 | 17 | - | 18634 | 31545 | 481 | 2 | 13 |
| NR_046261.1 | 5 | - | 874065 | 876366 | 2302 | 6673 | 6277 |
| XR_297366.1 | 1 | + | 28 | 5198 | 336 | 0 | 5 |
| XR_297367.1 | 2 | + | 5274 | 56792 | 1202 | 192 | 135 |
| XR_306384.1 | 18 | - | 26313 | 77420 | 1182 | 158 | 130 |
| XR_309492.1 | 18 | + | 45085 | 98936 | 990 | 299 | 211 |
| XR_309493.1 | 1 | + | 45085 | 98936 | 1197 | 313 | 242 |
| XR_309494.1 | 18 | + | 45085 | 98936 | 896 | 232 | 178 |
| XR_309495.1 | 4 | + | 45085 | 98936 | 1023 | 315 | 205 |
| XR_300123.1 | 6 | + | 131173 | 151642 | 1332 | 37 | 35 |
| XR_299891.1 | 14 | - | 166082 | 192797 | 542 | 12 | 13 |
| XR_298276.1 | 14 | - | 182684 | 186568 | 707 | 15 | 17 |
| XR_298277.1 | 1 | - | 182684 | 186568 | 797 | 17 | 20 |
| XR_305140.1 | 18 | - | 199151 | 223896 | 852 | 29 | 27 |
| XR_297836.1 | 15 | - | 322148 | 345957 | 537 | 83 | 59 |
| XR_299958.1 | 12 | - | 328816 | 331790 | 1073 | 71 | 48 |
| XR_306378.1 | 7 | + | 432714 | 443781 | 2490 | 646 | 447 |
| XR_303676.1 | 16 | - | 439297 | 442945 | 1682 | 265 | 248 |
| XR_304230.1 | 11 | + | 581928 | 593488 | 469 | 0 | 4 |
| XR_298284.1 | 11 | + | 618310 | 621618 | 738 | 336 | 343 |
| XR_304232.1 | 12 | - | 680092 | 714150 | 361 | 1 | 3 |
| XR_304233.1 | 12 | - | 680092 | 714150 | 565 | 2 | 11 |
| XR_299217.1 | 12 | + | 682598 | 687987 | 1328 | 204 | 114 |
| XR_307311.1 | 8 | + | 906135 | 911968 | 323 | 274 | 232 |
| XR_300468.1 | 8 | + | 940767 | 949860 | 328 | 318 | 261 |
| XR_308603.1 | 15 | - | 965008 | 1061153 | 383 | 6 | 5 |
| XR_308604.1 | 15 | - | 965008 | 1061153 | 415 | 8 | 6 |
| XR_305150.1 | 3 | + | 1021095 | 1028514 | 794 | 5 | 3 |
| XR_305151.1 | 3 | + | 1021095 | 1028514 | 788 | 5 | 3 |
| XR_305779.1 | 4 | + | 1172233 | 1553357 | 415 | 3 | 6 |
| XR_304716.1 | 4 | - | 1379977 | 1387781 | 1134 | 276 | 212 |
| XR_304717.1 | 11 | - | 1379977 | 1387781 | 531 | 92 | 77 |
| XR_304718.1 | 11 | - | 1379977 | 1387781 | 863 | 116 | 86 |
| XR_304719.1 | 11 | - | 1379977 | 1387781 | 657 | 111 | 95 |
| XR_306060.1 | 11 | + | 1507978 | 1530144 | 594 | 0 | 4 |
| XR_306061.1 | 12 | + | 1507978 | 1530144 | 773 | 4 | 3 |
| XR_306389.1 | 12 | - | 1589393 | 1593956 | 626 | 2 | 5 |
| XR_299793.1 | 1 | + | 1626075 | 1627631 | 722 | 3 | 15 |
| XR_297898.1 | 9 | + | 1751921 | 1769373 | 652 | 10 | 2 |
| XR_304239.1 | 14 | - | 1789792 | 1792644 | 559 | 70 | 73 |
| XR_306064.1 | 14 | - | 1895215 | 1964506 | 209 | 1 | 6 |
| XR_306066.1 | 4 | + | 1964560 | 2039952 | 1476 | 22 | 75 |
| XR_306067.1 | 4 | + | 1964560 | 2039952 | 1274 | 20 | 71 |
| XR_306068.1 | 4 | + | 1964560 | 2039952 | 1299 | 22 | 73 |
| XR_308937.1 | 18 | + | 1969927 | 2162518 | 825 | 10 | 22 |
| XR_297899.1 | 18 | + | 2120936 | 2128724 | 558 | 4 | 9 |
| XR_297900.1 | 18 | + | 2120936 | 2128724 | 543 | 4 | 9 |
| XR_304722.1 | 13 | - | 2145868 | 2150419 | 741 | 18 | 21 |
| XR_307914.1 | 13 | - | 2179531 | 2210186 | 925 | 154 | 43 |
| XR_304242.1 | 18 | - | 2349870 | 2379225 | 1174 | 132 | 161 |
| XR_304243.1 | 5 | - | 2349870 | 2379225 | 1048 | 139 | 181 |
| XR_304244.1 | 8 | + | 2379295 | 2380731 | 431 | 145 | 110 |
| XR_304245.1 | 12 | + | 2379295 | 2380731 | 540 | 164 | 127 |
| XR_307916.1 | 1 | - | 2411570 | 2499097 | 241 | 61 | 40 |
| XR_306071.1 | 10 | + | 2422011 | 2430626 | 588 | 1 | 5 |
| XR_304257.1 | 14 | - | 2464832 | 2481622 | 760 | 8 | 15 |
| XR_309172.1 | 14 | - | 2538768 | 2639664 | 614 | 2 | 0 |
| XR_309173.1 | 17 | - | 2538768 | 2639664 | 701 | 2 | 0 |
| XM_005654345.1 | 17 | + | 2623516 | 2706416 | 1218 | 7 | 6 |
| XR_309174.1 | 17 | - | 2639713 | 2664859 | 383 | 2 | 1 |
| XR_305780.1 | 17 | - | 2736799 | 2761968 | 707 | 9 | 8 |
| XR_297358.1 | 15 | + | 2810097 | 2814164 | 737 | 57 | 34 |
| XR_297359.1 | 7 | + | 2810097 | 2814164 | 717 | 57 | 34 |
| XR_301540.1 | 10 | - | 2810557 | 2812346 | 1309 | 39 | 33 |
| XR_308943.1 | 7 | - | 3037011 | 3060467 | 989 | 12 | 14 |
| XR_308944.1 | 13 | - | 3037011 | 3060467 | 1103 | 13 | 16 |
| XR_306659.1 | 2 | + | 3178202 | 3202080 | 673 | 1 | 2 |
| XR_306660.1 | 6 | + | 3178202 | 3202080 | 517 | 2 | 2 |
| XR_308609.1 | 14 | - | 3290461 | 3294107 | 1221 | 1 | 3 |
| XR_308610.1 | 1 | - | 3290461 | 3294107 | 2262 | 3 | 9 |
| XR_308611.1 | X | - | 3290461 | 3294107 | 1226 | 1 | 3 |
| XR_309177.1 | X | + | 3369026 | 3703800 | 922 | 62 | 45 |
| XR_302045.1 | 6 | + | 3382570 | 3478292 | 895 | 2 | 21 |
| XR_302046.1 | 14 | + | 3547515 | 3552591 | 1162 | 431 | 455 |
| XR_298891.1 | 14 | - | 3756020 | 3760419 | 552 | 1 | 7 |
| XR_302047.1 | 2 | + | 3775384 | 3779962 | 1674 | 2 | 4 |
| XR_302048.1 | 1 | + | 3775384 | 3779962 | 1453 | 6 | 6 |
| XR_305784.1 | 1 | - | 3841104 | 3845603 | 532 | 1 | 2 |
| XR_299923.1 | 9 | - | 3915197 | 4013693 | 333 | 12 | 17 |
| XR_299924.1 | 2 | - | 3915197 | 4013693 | 348 | 13 | 17 |
| XR_303325.1 | 7 | + | 3927264 | 3936743 | 648 | 2 | 12 |
| XR_302739.1 | X | - | 3961818 | 3976455 | 507 | 2 | 3 |
| XR_297626.1 | X | + | 4055369 | 4109901 | 1882 | 12 | 22 |
| XR_297625.1 | 16 | - | 4069165 | 4073224 | 702 | 2 | 11 |
| XR_302052.1 | 5 | - | 4104293 | 4110645 | 352 | 22 | 22 |
| XR_303326.1 | 16 | + | 4219476 | 4222502 | 672 | 93 | 76 |
| XR_303327.1 | 16 | + | 4219476 | 4222502 | 617 | 93 | 75 |
| XR_303328.1 | 2 | + | 4219476 | 4222502 | 644 | 92 | 75 |
| XR_299176.1 | 3 | + | 4226010 | 4238806 | 370 | 6 | 7 |
| XR_309180.1 | 3 | + | 4248583 | 4498930 | 1235 | 15 | 11 |
| XR_304269.1 | 3 | - | 4387725 | 4414698 | 411 | 1 | 5 |
| XR_308955.1 | 17 | - | 4454063 | 4578366 | 1457 | 19 | 17 |
| XM_005653059.1 | 5 | + | 4467890 | 4531254 | 866 | 14 | 25 |
| XR_297632.1 | 13 | + | 4526068 | 4529313 | 633 | 836 | 708 |
| XR_297633.1 | 12 | + | 4526068 | 4529313 | 742 | 1002 | 838 |
| XR_297634.1 | 10 | + | 4526068 | 4529313 | 607 | 799 | 677 |
| XR_302745.1 | 4 | - | 4534999 | 4549645 | 1150 | 2 | 7 |
| XR_302746.1 | 4 | - | 4534999 | 4549645 | 931 | 2 | 5 |
| XR_297636.1 | 4 | + | 4535438 | 4600837 | 1022 | 39 | 29 |
| XR_306398.1 | 4 | - | 4609396 | 4610555 | 750 | 1 | 5 |
| XR_309181.1 | 7 | + | 4623574 | 5221214 | 1738 | 285 | 326 |
| XR_135124.2 | 7 | - | 4641998 | 4649928 | 1633 | 221 | 292 |
| XR_304727.1 | 7 | - | 4681290 | 4725784 | 2550 | 85 | 141 |
| XR_299180.1 | 6 | - | 4694543 | 4731691 | 554 | 3 | 16 |
| XR_301556.1 | 8 | - | 4703859 | 4710320 | 580 | 8 | 7 |
| XR_298739.1 | 2 | + | 4802864 | 4808730 | 448 | 13 | 11 |
| XR_301561.1 | 17 | - | 4906027 | 4909010 | 901 | 25 | 21 |
| XR_299228.1 | 17 | - | 4953573 | 4960538 | 2349 | 3037 | 2674 |
| XR_299229.1 | 6 | - | 4953573 | 4960538 | 2715 | 3297 | 2839 |
| XR_308339.1 | X | - | 4972383 | 4987555 | 654 | 108 | 109 |
| XR_308340.1 | 5 | - | 4972383 | 4987555 | 609 | 91 | 85 |
| XR_301565.1 | 5 | + | 5047843 | 5052442 | 2024 | 215 | 172 |
| XR_302053.1 | 15 | + | 5228583 | 5230290 | 834 | 15 | 17 |
| XR_307374.1 | 15 | + | 5363601 | 5537586 | 661 | 2 | 8 |
| XR_306669.1 | 15 | - | 5427669 | 5444474 | 275 | 0 | 2 |
| XR_306085.1 | 15 | - | 5477898 | 5486849 | 1416 | 996 | 681 |
| XR_306086.1 | 15 | - | 5477898 | 5486849 | 2394 | 1255 | 922 |
| XM_003122469.2 | 15 | - | 5480791 | 5488639 | 1843 | 2 | 3 |
| XR_299385.1 | 15 | - | 5581584 | 5648727 | 346 | 0 | 1 |
| XR_297869.1 | 15 | - | 5602782 | 5607618 | 1128 | 19 | 40 |
| XR_297870.1 | 18 | + | 5726431 | 5779120 | 1686 | 368 | 284 |
| XR_297871.1 | 18 | + | 5726431 | 5779120 | 1778 | 240 | 171 |
| XR_297872.1 | 4 | + | 5726431 | 5779120 | 1847 | 257 | 179 |
| XR_303705.1 | 4 | - | 5858773 | 6254504 | 395 | 7 | 2 |
| XR_306399.1 | 6 | + | 6025677 | 6033646 | 390 | 5 | 9 |
| XR_303339.1 | 6 | + | 6062121 | 6069785 | 2007 | 34 | 80 |
| XR_299478.1 | 18 | - | 6094792 | 6192904 | 893 | 0 | 2 |
| XR_308345.1 | 17 | + | 6170442 | 6214121 | 783 | 61 | 6 |
| XR_308346.1 | 1 | + | 6170442 | 6214121 | 1197 | 150 | 31 |
| XR_304275.1 | 17 | - | 6278835 | 6305480 | 479 | 2 | 3 |
| XR_308347.1 | 17 | - | 6365179 | 6383725 | 2059 | 65 | 59 |
| XR_308348.1 | 17 | - | 6365179 | 6383725 | 1976 | 65 | 58 |
| XR_299594.1 | 17 | + | 6414332 | 6414767 | 746 | 1 | 8 |
| XR_309185.1 | 17 | + | 6454319 | 6460306 | 1781 | 92 | 81 |
| XR_302072.1 | 18 | - | 6467330 | 6469242 | 947 | 191 | 153 |
| XR_302073.1 | 9 | - | 6467330 | 6469242 | 1013 | 105 | 72 |
| XR_302074.1 | 6 | - | 6467330 | 6469242 | 1026 | 21 | 18 |
| XR_297536.1 | 6 | - | 6663723 | 6666018 | 745 | 78 | 72 |
| XR_297537.1 | 6 | - | 6663723 | 6666018 | 549 | 56 | 50 |
| XR_301571.1 | 6 | + | 6749941 | 6794116 | 1836 | 1495 | 2527 |
| XR_301572.1 | 7 | + | 6749941 | 6794116 | 1178 | 838 | 1433 |
| XR_301573.1 | 9 | + | 6749941 | 6794116 | 1671 | 1485 | 2517 |
| XR_301574.1 | 14 | + | 6749941 | 6794116 | 1768 | 1282 | 2129 |
| XR_115737.2 | 14 | - | 6803590 | 6811708 | 3275 | 25148 | 31677 |
| XR_299598.1 | 14 | + | 6869116 | 6891403 | 595 | 254 | 249 |
| XR_306105.1 | 3 | + | 7142448 | 7144571 | 447 | 33 | 41 |
| XR_306106.1 | 7 | + | 7142448 | 7144571 | 470 | 34 | 41 |
| XR_306102.1 | 7 | - | 7158285 | 7168410 | 646 | 80 | 88 |
| XR_306103.1 | 10 | - | 7158285 | 7168410 | 578 | 72 | 81 |
| XR_299688.1 | 10 | - | 7214449 | 7286319 | 975 | 198 | 127 |
| XR_300284.1 | 13 | + | 7282946 | 7294988 | 478 | 18 | 12 |
| XR_300605.1 | 2 | + | 7306696 | 7309868 | 574 | 0 | 1 |
| XR_309585.1 | 2 | - | 7352207 | 7375949 | 1787 | 2 | 9 |
| XR_300607.1 | 2 | - | 7488344 | 7504318 | 3240 | 27 | 44 |
| XR_300608.1 | 13 | - | 7488344 | 7504318 | 2943 | 21 | 38 |
| XR_302785.1 | 13 | - | 7753234 | 7759539 | 501 | 0 | 5 |
| XR_300613.1 | 3 | - | 8120290 | 8133925 | 671 | 3 | 3 |
| XR_308962.1 | 3 | + | 8168027 | 8200215 | 2614 | 423 | 630 |
| XR_308963.1 | 8 | + | 8168027 | 8200215 | 2399 | 418 | 620 |
| XR_299998.1 | 15 | - | 8311965 | 8313144 | 851 | 3 | 1 |
| XR_299999.1 | 15 | - | 8311965 | 8313144 | 824 | 3 | 1 |
| XR_300618.1 | 1 | - | 8346551 | 8395999 | 840 | 173 | 114 |
| XR_297179.1 | 11 | - | 8447570 | 8461924 | 899 | 6 | 7 |
| XR_297180.1 | 2 | - | 8447570 | 8461924 | 774 | 6 | 7 |
| XR_302794.1 | 6 | - | 8476600 | 8507377 | 337 | 0 | 4 |
| XR_297478.1 | 5 | + | 8563112 | 8589157 | 302 | 3 | 5 |
| XR_308625.1 | 5 | - | 8640948 | 8642429 | 786 | 198 | 140 |
| XR_306431.1 | 5 | + | 8714313 | 8736055 | 433 | 0 | 2 |
| XR_306427.1 | 5 | - | 8736144 | 8792336 | 1113 | 5 | 9 |
| XR_306428.1 | 5 | - | 8736144 | 8792336 | 1201 | 5 | 9 |
| XR_306429.1 | 1 | - | 8736144 | 8792336 | 1204 | 5 | 9 |
| XR_306430.1 | 1 | - | 8736144 | 8792336 | 1017 | 4 | 7 |
| XR_130373.3 | 15 | - | 8919230 | 8922581 | 995 | 2078 | 2454 |
| XR_301592.1 | 15 | - | 8919230 | 8922581 | 1285 | 2540 | 2985 |
| XR_301593.1 | 15 | - | 8919230 | 8922581 | 969 | 2026 | 2396 |
| XR_301594.1 | 15 | - | 8919230 | 8922581 | 965 | 2022 | 2386 |
| XR_301595.1 | 15 | - | 8919230 | 8922581 | 1289 | 2544 | 2995 |
| XR_301596.1 | 15 | - | 8919230 | 8922581 | 1182 | 2327 | 2736 |
| XR_301597.1 | 17 | - | 8919230 | 8922581 | 965 | 2009 | 2362 |
| XR_301598.1 | 17 | - | 8919230 | 8922581 | 1162 | 2308 | 2726 |
| XR_301599.1 | 2 | - | 8919230 | 8922581 | 676 | 1560 | 1855 |
| XR_300534.1 | 2 | + | 8941984 | 8947519 | 350 | 53 | 35 |
| XR_307329.1 | 2 | + | 9062798 | 9078825 | 559 | 0 | 1 |
| XR_297874.1 | 2 | - | 9175274 | 9187093 | 1014 | 510 | 347 |
| XR_297048.1 | 2 | + | 9244755 | 9375395 | 665 | 2 | 9 |
| XR_297049.1 | 2 | + | 9244755 | 9375395 | 650 | 2 | 9 |
| XR_301604.1 | 2 | - | 9283109 | 9287458 | 417 | 2 | 9 |
| XR_301605.1 | 2 | - | 9283109 | 9287458 | 573 | 3 | 12 |
| XR_301606.1 | 2 | - | 9283109 | 9287458 | 398 | 2 | 10 |
| XR_305181.1 | 17 | + | 9431425 | 9450355 | 412 | 1 | 2 |
| XR_297051.1 | 1 | + | 9505267 | 9523737 | 873 | 20 | 23 |
| XR_297052.1 | 1 | + | 9505267 | 9523737 | 936 | 20 | 24 |
| XR_297053.1 | 12 | + | 9505267 | 9523737 | 745 | 18 | 23 |
| XR_305184.1 | 6 | + | 9573979 | 9577014 | 1092 | 25 | 35 |
| XR_306676.1 | 6 | - | 9591537 | 9723825 | 256 | 0 | 3 |
| XR_309188.1 | 6 | - | 9641427 | 9649403 | 897 | 6 | 8 |
| XR_299109.1 | 1 | + | 9663619 | 9680532 | 1108 | 218 | 142 |
| XR_135127.2 | 9 | - | 9663891 | 9671792 | 1822 | 4067 | 2173 |
| XR_305188.1 | 9 | - | 9770962 | 9787323 | 3110 | 128 | 71 |
| XR_305189.1 | 6 | - | 9770962 | 9787323 | 3247 | 135 | 74 |
| XR_302802.1 | 6 | + | 9863181 | 9886339 | 390 | 0 | 1 |
| XR_304748.1 | 6 | + | 10020972 | 10388939 | 1233 | 98 | 64 |
| XR_304749.1 | 6 | + | 10020972 | 10388939 | 1221 | 97 | 64 |
| XR_305795.1 | 6 | - | 10145763 | 10153615 | 582 | 77 | 71 |
| XR_306434.1 | 6 | + | 10450512 | 10461010 | 892 | 3 | 11 |
| XR_306435.1 | 6 | + | 10450512 | 10461010 | 990 | 4 | 13 |
| XR_306436.1 | 6 | + | 10450512 | 10461010 | 1130 | 7 | 15 |
| XR_300340.1 | 6 | - | 10464447 | 11084641 | 1431 | 12 | 9 |
| XR_305192.1 | 2 | - | 10479235 | 10519239 | 735 | 5 | 8 |
| XR_305193.1 | 1 | - | 10614982 | 10627969 | 774 | 18 | 35 |
| XR_305194.1 | 9 | - | 10614982 | 10627969 | 585 | 62 | 91 |
| XR_303376.1 | 8 | - | 10692884 | 10694610 | 702 | 11 | 12 |
| XR_298295.1 | 8 | - | 10720719 | 10788751 | 1085 | 8 | 26 |
| XR_304753.1 | 8 | - | 10749018 | 10754173 | 229 | 27 | 34 |
| XR_304754.1 | 8 | - | 10871923 | 10874079 | 687 | 86 | 56 |
| XR_304292.1 | 8 | + | 10923375 | 10930957 | 1891 | 20 | 34 |
| XR_304293.1 | 3 | + | 10923375 | 10930957 | 1860 | 20 | 33 |
| XR_302110.1 | 3 | - | 10935613 | 10948922 | 518 | 6 | 23 |
| XM_005667926.1 | 14 | - | 10986225 | 11053748 | 1838 | 182 | 142 |
| XR_302118.1 | 14 | + | 11089321 | 11191336 | 1041 | 4 | 19 |
| XR_302119.1 | 4 | + | 11089321 | 11191336 | 1035 | 5 | 17 |
| XR_298303.1 | 4 | + | 11097048 | 11173783 | 523 | 1 | 13 |
| XR_302120.1 | 5 | - | 11704931 | 11706880 | 1214 | 976 | 737 |
| XR_302121.1 | 14 | + | 11863094 | 11869395 | 1464 | 162 | 133 |
| XR_299234.1 | 14 | + | 11996303 | 12005313 | 1452 | 110 | 116 |
| XR_299235.1 | 8 | + | 11996303 | 12005313 | 1712 | 94 | 111 |
| XR_299236.1 | 8 | + | 11996303 | 12005313 | 1648 | 89 | 106 |
| XR_302122.1 | 8 | + | 12040049 | 12206900 | 2050 | 46 | 42 |
| XR_304302.1 | 11 | + | 12066555 | 12071632 | 651 | 1 | 5 |
| XR_303721.1 | 2 | - | 12096784 | 12108318 | 437 | 24 | 18 |
| XR_302129.1 | 8 | + | 12217244 | 12530755 | 741 | 5 | 21 |
| XR_304761.1 | 8 | - | 12363420 | 12526103 | 1126 | 180 | 85 |
| XR_298408.1 | 8 | - | 12391379 | 12402575 | 450 | 3 | 6 |
| XR_302125.1 | X | - | 12497227 | 12589268 | 526 | 2 | 5 |
| XR_302126.1 | X | - | 12497227 | 12589268 | 564 | 6 | 5 |
| XR_302127.1 | X | - | 12497227 | 12589268 | 469 | 1 | 8 |
| XM_003355752.3 | X | - | 12555655 | 12668807 | 1177 | 1941 | 1514 |
| XR_308976.1 | X | + | 12599771 | 12601402 | 851 | 14 | 10 |
| XR_302130.1 | X | - | 12647595 | 12662529 | 514 | 4 | 8 |
| XR_298411.1 | X | - | 13171373 | 13255150 | 425 | 0 | 4 |
| XM_005653245.1 | 3 | - | 13228712 | 13233729 | 1156 | 355 | 316 |
| XR_306138.1 | 9 | + | 13252736 | 13322683 | 526 | 209 | 155 |
| XR_300147.1 | 8 | + | 13327184 | 13368613 | 355 | 6 | 4 |
| XR_297376.1 | 2 | + | 13390488 | 13394765 | 2063 | 1972 | 1920 |
| XR_298412.1 | 8 | + | 13437411 | 13485695 | 1117 | 175 | 172 |
| XR_298413.1 | 18 | + | 13437411 | 13485695 | 3028 | 746 | 651 |
| XR_302816.1 | 18 | + | 13493396 | 14097380 | 575 | 1 | 3 |
| XR_302817.1 | 18 | + | 13493396 | 14097380 | 464 | 1 | 2 |
| XM_005668674.1 | 18 | + | 13655093 | 13674213 | 679 | 18 | 27 |
| XR_303725.1 | 18 | + | 13712109 | 13717367 | 720 | 60 | 51 |
| XR_135462.2 | 13 | + | 13998498 | 14000927 | 684 | 10 | 6 |
| XR_306705.1 | 1 | + | 14043271 | 14050196 | 699 | 77 | 79 |
| XR_306703.1 | 2 | + | 14084559 | 14093205 | 1096 | 0 | 3 |
| XR_306704.1 | 3 | + | 14084559 | 14093205 | 1049 | 0 | 3 |
| XR_302818.1 | 4 | + | 14137818 | 14142353 | 510 | 1 | 3 |
| XR_300629.1 | 6 | - | 14148312 | 14151214 | 1441 | 139 | 185 |
| XR_308984.1 | 1 | - | 14295649 | 14298567 | 2837 | 1321 | 1344 |
| XR_298421.1 | 1 | + | 14394846 | 14536289 | 818 | 5 | 4 |
| XR_305804.1 | 7 | - | 14544284 | 14551457 | 1555 | 355 | 201 |
| XR_299722.1 | 11 | + | 14669922 | 14686944 | 611 | 4 | 0 |
| XR_299723.1 | 11 | + | 14669922 | 14686944 | 603 | 4 | 0 |
| XR_299724.1 | 11 | + | 14669922 | 14686944 | 757 | 7 | 1 |
| XR_305215.1 | 6 | - | 14678048 | 15984452 | 709 | 3 | 3 |
| XR_306156.1 | 12 | - | 14728270 | 14953801 | 539 | 730 | 456 |
| XR_299396.1 | 12 | - | 14780943 | 14806588 | 781 | 85 | 54 |
| XR_306462.1 | 13 | - | 14867795 | 14868220 | 787 | 236 | 162 |
| XR_309593.1 | 13 | + | 14910931 | 14954936 | 947 | 15 | 24 |
| XM_003361749.2 | 13 | + | 14985063 | 14998077 | 2576 | 439 | 405 |
| XM_005674360.1 | 13 | + | 14985063 | 14998077 | 2545 | 378 | 360 |
| XM_005674361.1 | 3 | + | 14985063 | 14998077 | 2465 | 388 | 360 |
| XR_299397.1 | 3 | + | 15059720 | 15063203 | 1647 | 15 | 28 |
| XR_299308.1 | 3 | + | 15177516 | 15188311 | 1271 | 76 | 39 |
| XR_302822.1 | 9 | - | 15204895 | 15263310 | 924 | 48 | 34 |
| XR_302823.1 | 15 | - | 15204895 | 15263310 | 835 | 48 | 34 |
| XR_299868.1 | X | - | 15305441 | 15311435 | 893 | 56 | 29 |
| XR_306159.1 | 7 | + | 15410633 | 15449633 | 491 | 292 | 252 |
| XR_303388.1 | 2 | - | 15761619 | 15805539 | 1050 | 207 | 233 |
| XR_297637.1 | 4 | - | 15887828 | 15892358 | 480 | 10 | 16 |
| XR_306167.1 | 4 | + | 15953097 | 15971119 | 667 | 2 | 18 |
| XR_308988.1 | 7 | - | 15969261 | 15988025 | 1458 | 22 | 14 |
| XR_308989.1 | 1 | + | 15988143 | 16012886 | 1357 | 3 | 2 |
| XR_297134.1 | 2 | + | 16006878 | 16008530 | 649 | 113 | 102 |
| XR_297135.1 | 8 | + | 16006878 | 16008530 | 587 | 108 | 91 |
| XR_307358.1 | 5 | - | 16149060 | 16190968 | 1556 | 2 | 3 |
| XR_307359.1 | 5 | - | 16149060 | 16190968 | 1230 | 3 | 0 |
| XR_302835.1 | 1 | + | 16352414 | 16403015 | 642 | 2 | 12 |
| XR_304313.1 | 4 | + | 16626169 | 16675063 | 473 | 8 | 19 |
| XR_302139.1 | 9 | - | 16639695 | 16640791 | 982 | 116 | 68 |
| XR_302142.1 | 13 | - | 16765349 | 16812315 | 993 | 83 | 54 |
| XR_302143.1 | 1 | - | 16765349 | 16812315 | 1318 | 41 | 31 |
| XR_300645.1 | 1 | + | 16819952 | 16843000 | 680 | 5 | 18 |
| XR_304317.1 | 1 | + | 16946458 | 17354912 | 1761 | 138 | 480 |
| XR_305809.1 | 1 | - | 16946932 | 16966152 | 1043 | 2 | 11 |
| XR_307942.1 | 1 | - | 17034425 | 17037005 | 717 | 76 | 58 |
| XR_306176.1 | Unknown | + | 17049798 | 17058769 | 489 | 3 | 7 |
| XR_306719.1 | 2 | - | 17157289 | 17232237 | 616 | 7 | 4 |
| XR_306720.1 | 9 | - | 17157289 | 17232237 | 675 | 8 | 6 |
| XR_299238.1 | 9 | + | 17211652 | 17247553 | 374 | 7 | 8 |
| XR_309442.1 | 12 | - | 17426474 | 17457682 | 273 | 3 | 2 |
| XR_298170.1 | 1 | + | 17431081 | 17439311 | 444 | 8 | 18 |
| XR_298516.1 | 1 | + | 17433269 | 17451683 | 1095 | 129 | 103 |
| XR_297579.1 | 1 | + | 17441409 | 17468510 | 368 | 22 | 8 |
| XM_005653793.1 | 4 | + | 17475266 | 17497060 | 2823 | 51467 | 43053 |
| XR_306178.1 | 4 | - | 17568037 | 17653511 | 1287 | 19 | 13 |
| XR_306179.1 | 12 | - | 17568037 | 17653511 | 1215 | 19 | 13 |
| XR_306180.1 | 3 | - | 17568037 | 17653511 | 1169 | 18 | 13 |
| XR_306721.1 | 3 | + | 17642049 | 17654981 | 859 | 22 | 14 |
| XR_306467.1 | 3 | - | 17690894 | 17767955 | 428 | 24 | 10 |
| XR_301648.1 | 3 | - | 17703252 | 17706852 | 1864 | 7 | 12 |
| XR_301649.1 | 7 | - | 17703252 | 17706852 | 1871 | 7 | 12 |
| XR_301650.1 | 7 | - | 17703252 | 17706852 | 1856 | 7 | 12 |
| XR_301651.1 | 7 | - | 17703252 | 17706852 | 2076 | 7 | 12 |
| XR_301652.1 | 3 | + | 17803684 | 17809726 | 2222 | 212 | 160 |
| XR_301653.1 | 5 | + | 17803684 | 17809726 | 2219 | 212 | 160 |
| XR_301654.1 | 10 | + | 17803684 | 17809726 | 2178 | 211 | 158 |
| XR_297301.1 | 10 | + | 17809852 | 17810985 | 704 | 60 | 44 |
| XR_298312.1 | 15 | + | 18003973 | 18145663 | 394 | 5 | 1 |
| XR_298313.1 | 1 | + | 18003973 | 18145663 | 364 | 4 | 1 |
| XR_306474.1 | 2 | - | 18121672 | 18142760 | 975 | 17 | 48 |
| XR_306475.1 | 4 | - | 18121672 | 18142760 | 982 | 18 | 48 |
| XR_306476.1 | 4 | - | 18121672 | 18142760 | 1029 | 18 | 41 |
| XR_306477.1 | 2 | - | 18121672 | 18142760 | 795 | 15 | 40 |
| XR_306478.1 | 3 | - | 18121672 | 18142760 | 827 | 15 | 44 |
| XR_306479.1 | 4 | - | 18121672 | 18142760 | 1032 | 18 | 41 |
| XR_306480.1 | 7 | - | 18121672 | 18142760 | 1114 | 19 | 51 |
| XR_306481.1 | 5 | - | 18121672 | 18142760 | 1203 | 32 | 53 |
| XR_301655.1 | 5 | + | 18266340 | 18270062 | 324 | 96 | 103 |
| XR_301656.1 | 9 | + | 18266340 | 18270062 | 286 | 78 | 79 |
| XR_301657.1 | 9 | + | 18266340 | 18270062 | 313 | 52 | 56 |
| XR_300647.1 | 9 | - | 18302411 | 18329596 | 1092 | 0 | 4 |
| XR_300648.1 | 17 | - | 18302411 | 18329596 | 950 | 0 | 4 |
| XR_304318.1 | 4 | - | 18328618 | 18365002 | 231 | 1 | 4 |
| XM_001924386.4 | 10 | - | 18436267 | 18458625 | 2681 | 529 | 495 |
| XM_005652562.1 | 15 | - | 18436267 | 18458625 | 3989 | 580 | 547 |
| XR_301658.1 | 15 | + | 18448312 | 18471502 | 681 | 75 | 69 |
| XR_297140.1 | 18 | - | 18458793 | 18472824 | 1674 | 24 | 26 |
| XR_300650.1 | 4 | + | 18513347 | 18900691 | 817 | 15 | 11 |
| XR_300651.1 | 4 | + | 18513347 | 18900691 | 884 | 15 | 11 |
| XR_300652.1 | X | + | 18513347 | 18900691 | 1492 | 21 | 30 |
| XR_300653.1 | 6 | + | 18513347 | 18900691 | 1425 | 21 | 30 |
| XR_115809.2 | 1 | - | 18530777 | 18533229 | 786 | 126 | 175 |
| XR_302149.1 | 8 | - | 18530777 | 18533229 | 536 | 80 | 110 |
| XR_297142.1 | 6 | - | 18586012 | 18588636 | 865 | 55 | 45 |
| XR_297143.1 | 4 | - | 18586012 | 18588636 | 971 | 58 | 42 |
| XR_297144.1 | 15 | - | 18586012 | 18588636 | 865 | 54 | 45 |
| XR_297145.1 | 4 | - | 18586012 | 18588636 | 866 | 42 | 37 |
| XR_297146.1 | 9 | - | 18586012 | 18588636 | 966 | 58 | 42 |
| XR_297147.1 | 9 | - | 18586012 | 18588636 | 926 | 56 | 45 |
| XR_301659.1 | 14 | - | 18618270 | 18750659 | 522 | 3 | 4 |
| XR_299193.1 | 4 | - | 18882747 | 18886928 | 530 | 12 | 14 |
| XR_299752.1 | 4 | - | 18912542 | 19353650 | 1399 | 2708 | 860 |
| XR_301667.1 | 4 | - | 19082206 | 19154699 | 727 | 4 | 20 |
| XR_304794.1 | 1 | + | 19136395 | 19182778 | 443 | 20 | 9 |
| XR_303394.1 | 6 | - | 19291553 | 19293988 | 864 | 107 | 143 |
| XR_303391.1 | 8 | + | 19339311 | 19344340 | 838 | 8 | 4 |
| XR_303392.1 | 8 | + | 19339311 | 19344340 | 393 | 2 | 8 |
| XR_303393.1 | 15 | + | 19339311 | 19344340 | 432 | 3 | 4 |
| XR_115919.2 | 15 | - | 19344435 | 19350326 | 862 | 6 | 8 |
| XR_297055.1 | 1 | + | 19384252 | 19406639 | 551 | 0 | 6 |
| XR_304804.1 | 1 | + | 19475679 | 19478992 | 571 | 1 | 16 |
| XR_303752.1 | 13 | + | 19692737 | 19704721 | 631 | 7 | 8 |
| XR_309198.1 | 6 | - | 19792655 | 19799197 | 807 | 1 | 11 |
| XR_116026.2 | 7 | + | 19835280 | 19845450 | 720 | 140 | 138 |
| XR_303406.1 | 12 | + | 19921830 | 19931748 | 399 | 0 | 7 |
| XR_307378.1 | 7 | - | 19946487 | 19957224 | 628 | 5 | 11 |
| XR_307948.1 | 7 | + | 20021662 | 20090628 | 344 | 18 | 6 |
| XR_305239.1 | 11 | + | 20034334 | 20036505 | 435 | 21 | 12 |
| XR_305831.1 | 16 | - | 20344684 | 20401547 | 1852 | 320 | 204 |
| XR_305241.1 | 17 | + | 20378201 | 20393092 | 1377 | 279 | 223 |
| XR_304328.1 | 17 | - | 20424851 | 20428737 | 410 | 0 | 4 |
| XR_298745.1 | 18 | - | 21022303 | 21024557 | 569 | 35 | 31 |
| XR_298746.1 | 12 | - | 21022303 | 21024557 | 574 | 31 | 28 |
| XR_303407.1 | 14 | + | 21175893 | 21177239 | 925 | 163 | 187 |
| XR_302154.1 | 14 | - | 21415241 | 21465881 | 1039 | 31 | 45 |
| XR_309009.1 | 3 | - | 21517358 | 21554962 | 989 | 7 | 22 |
| XR_304333.1 | 1 | + | 21559156 | 21564456 | 490 | 593 | 410 |
| XR_302157.1 | 2 | - | 21647940 | 21649332 | 1408 | 3823 | 6793 |
| XR_299534.1 | 5 | + | 21725515 | 21737922 | 248 | 1 | 4 |
| XR_305248.1 | 8 | + | 21761852 | 21918741 | 729 | 79 | 60 |
| XR_305249.1 | 13 | + | 21761852 | 21918741 | 1211 | 82 | 74 |
| XR_297927.1 | 14 | + | 22081617 | 22085731 | 613 | 7 | 9 |
| XR_297928.1 | 3 | + | 22081617 | 22085731 | 602 | 7 | 6 |
| XR_297929.1 | 3 | + | 22081617 | 22085731 | 554 | 7 | 7 |
| XR_307381.1 | 6 | + | 22139193 | 22166328 | 483 | 2 | 3 |
| XR_307382.1 | 13 | + | 22139193 | 22166328 | 626 | 2 | 4 |
| XR_297648.1 | 1 | + | 22185242 | 22190351 | 609 | 28 | 43 |
| XR_302846.1 | 2 | + | 22261900 | 22601897 | 418 | 3 | 5 |
| XR_302847.1 | 4 | + | 22261900 | 22601897 | 481 | 3 | 6 |
| XR_302848.1 | X | + | 22506613 | 22524212 | 922 | 3 | 18 |
| XR_297655.1 | 6 | - | 22726717 | 22738585 | 1279 | 14 | 11 |
| XR_297656.1 | 8 | - | 22726717 | 22738585 | 1269 | 14 | 9 |
| XR_297657.1 | 12 | - | 22726717 | 22738585 | 1147 | 14 | 10 |
| XR_301672.1 | 12 | + | 23027900 | 23095128 | 885 | 1 | 4 |
| XR_308379.1 | 12 | + | 23146874 | 23235019 | 613 | 10 | 14 |
| XR_309014.1 | 15 | + | 23229956 | 23251722 | 494 | 9 | 13 |
| XR_309015.1 | 6 | + | 23229956 | 23251722 | 499 | 10 | 12 |
| XR_304362.1 | 17 | + | 23321059 | 23348248 | 371 | 1 | 11 |
| XR_304364.1 | 3 | - | 23469282 | 23473202 | 859 | 7 | 10 |
| XR_304363.1 | 3 | + | 23594056 | 23596818 | 779 | 87 | 126 |
| XR_304368.1 | 4 | - | 23669156 | 23670345 | 744 | 44 | 43 |
| XR_304369.1 | 4 | - | 23669156 | 23670345 | 670 | 38 | 38 |
| XR_304370.1 | 2 | - | 23669156 | 23670345 | 740 | 44 | 42 |
| XR_307386.1 | 16 | + | 23674347 | 23678986 | 1456 | 4 | 15 |
| XR_307387.1 | 16 | + | 23674347 | 23678986 | 1239 | 4 | 14 |
| XR_300041.1 | 10 | - | 23815136 | 23904331 | 201 | 7 | 10 |
| XR_299737.1 | 10 | + | 23933356 | 23935759 | 1219 | 25912 | 26696 |
| XR_299738.1 | 10 | + | 23933356 | 23935759 | 1223 | 25938 | 26716 |
| XR_297670.1 | 11 | - | 24172933 | 24192804 | 382 | 26 | 53 |
| XR_299194.1 | 1 | - | 24228313 | 24230488 | 382 | 3 | 5 |
| XR_304374.1 | 14 | + | 24232784 | 24233930 | 759 | 408 | 477 |
| XR_297305.1 | 8 | + | 24313518 | 24366018 | 394 | 53 | 20 |
| XR_297306.1 | 10 | + | 24313518 | 24366018 | 410 | 54 | 21 |
| XR_297307.1 | 15 | + | 24313518 | 24366018 | 420 | 54 | 20 |
| XR_303420.1 | 2 | + | 24332129 | 24344564 | 459 | 0 | 2 |
| XR_299195.1 | 2 | + | 24527828 | 24544088 | 659 | 1 | 7 |
| XM_005663945.1 | 2 | - | 24703594 | 24712895 | 1081 | 51 | 51 |
| XR_299755.1 | 2 | - | 24730726 | 24790284 | 343 | 2 | 1 |
| XR_297250.1 | 4 | - | 24837255 | 24853726 | 971 | 11 | 21 |
| XR_297251.1 | 12 | - | 24837255 | 24853726 | 895 | 11 | 16 |
| XR_297672.1 | 6 | - | 24838726 | 24858994 | 4155 | 37 | 135 |
| XR_297673.1 | 6 | - | 24838726 | 24858994 | 4979 | 42 | 181 |
| XR_300050.1 | 13 | + | 24843411 | 24854919 | 426 | 32 | 26 |
| XM_005665845.1 | 1 | - | 24980348 | 24992759 | 1201 | 135 | 74 |
| XR_304375.1 | 10 | - | 24980348 | 24992759 | 514 | 31 | 35 |
| XR_307961.1 | 10 | - | 25031770 | 25037665 | 427 | 1 | 6 |
| XR_297591.1 | 10 | - | 25034746 | 25078959 | 868 | 50 | 36 |
| XR_300667.1 | 1 | + | 25124567 | 25134196 | 416 | 2 | 7 |
| XM_005661028.1 | X | - | 25240466 | 25271631 | 916 | 148 | 145 |
| XR_309025.1 | 6 | + | 25280849 | 25524222 | 1460 | 5 | 14 |
| XR_309026.1 | 6 | + | 25280849 | 25524222 | 1626 | 7 | 20 |
| XR_307967.1 | 13 | - | 25318181 | 25328679 | 560 | 3 | 18 |
| XR_308671.1 | 13 | - | 25353422 | 25363527 | 650 | 13 | 14 |
| XR_308672.1 | 9 | - | 25353422 | 25363527 | 545 | 12 | 13 |
| XR_304814.1 | 9 | + | 25448743 | 25457814 | 328 | 0 | 3 |
| XR_304815.1 | 9 | + | 25448743 | 25457814 | 356 | 0 | 4 |
| XM_005667232.1 | 9 | - | 26094004 | 26103124 | 1127 | 209 | 177 |
| XR_298522.1 | 13 | - | 26466891 | 26919430 | 529 | 3 | 20 |
| XR_300671.1 | 3 | + | 26499195 | 26529634 | 1339 | 19 | 16 |
| XR_130939.2 | 9 | - | 26587046 | 26622941 | 784 | 1543 | 895 |
| XR_306751.1 | 9 | - | 26668575 | 26669126 | 1433 | 37 | 51 |
| XR_306752.1 | 7 | - | 26668575 | 26669126 | 1474 | 40 | 58 |
| XR_308679.1 | 7 | - | 26753047 | 26759944 | 278 | 8 | 19 |
| XR_308680.1 | X | + | 26804722 | 26807347 | 598 | 1073 | 702 |
| XR_300673.1 | 14 | + | 26966686 | 26973753 | 364 | 2 | 4 |
| XR_301693.1 | 2 | - | 27005619 | 27025833 | 1747 | 36 | 38 |
| XR_301694.1 | 3 | - | 27005619 | 27025833 | 2306 | 15 | 27 |
| XR_304382.1 | 3 | - | 27128045 | 27195431 | 201 | 0 | 1 |
| XR_299114.1 | 5 | + | 27215721 | 27222863 | 458 | 13 | 27 |
| XR_307394.1 | 5 | - | 27281995 | 27307521 | 698 | 3 | 14 |
| XR_308385.1 | 5 | + | 27610192 | 27615979 | 261 | 38 | 30 |
| XR_308386.1 | 4 | + | 27610192 | 27615979 | 433 | 47 | 34 |
| XR_308387.1 | 11 | + | 27705062 | 27718003 | 573 | 8 | 4 |
| XR_299536.1 | X | - | 27748943 | 27759477 | 1116 | 120 | 120 |
| XR_299116.1 | X | + | 27846282 | 27848624 | 1146 | 215 | 283 |
| XR_297933.1 | 6 | + | 27935090 | 27941357 | 345 | 19 | 5 |
| XR_301700.1 | 6 | - | 28358562 | 28411612 | 1255 | 710 | 832 |
| XR_301701.1 | 13 | - | 28358562 | 28411612 | 1108 | 615 | 708 |
| XR_301702.1 | 6 | - | 28358562 | 28411612 | 845 | 156 | 206 |
| XR_301703.1 | 7 | - | 28486730 | 28499066 | 901 | 84 | 77 |
| XR_301704.1 | 18 | - | 28486730 | 28499066 | 1156 | 34 | 33 |
| XR_301705.1 | 6 | - | 28486730 | 28499066 | 1974 | 137 | 130 |
| XR_301706.1 | 6 | - | 28486730 | 28499066 | 1812 | 129 | 126 |
| XR_304390.1 | 8 | + | 28765547 | 28766251 | 429 | 239 | 193 |
| XR_299121.1 | 15 | - | 29051268 | 29066111 | 578 | 15 | 18 |
| XR_299539.1 | 16 | - | 29062515 | 29111834 | 2795 | 64 | 48 |
| XR_308688.1 | 17 | - | 29708509 | 29719526 | 637 | 4 | 13 |
| XR_308689.1 | 17 | - | 29708509 | 29719526 | 761 | 3 | 15 |
| XR_301708.1 | 17 | + | 29800960 | 29826690 | 838 | 11 | 7 |
| XR_304396.1 | 2 | - | 29807822 | 29815476 | 244 | 268 | 171 |
| NR_045041.1 | 8 | - | 29837647 | 30025429 | 1962 | 134 | 135 |
| XR_297387.1 | 9 | - | 30372774 | 30386941 | 823 | 4 | 8 |
| XR_302872.1 | 13 | + | 30409647 | 30432093 | 529 | 4 | 3 |
| XR_305870.1 | 13 | - | 30444033 | 30450757 | 1231 | 22 | 13 |
| XR_305871.1 | 3 | - | 30444033 | 30450757 | 1180 | 20 | 12 |
| XR_305872.1 | 3 | - | 30490119 | 30494843 | 334 | 6 | 7 |
| XR_300187.1 | 6 | - | 30515158 | 30570985 | 917 | 112 | 62 |
| XR_300188.1 | 6 | - | 30515158 | 30570985 | 913 | 112 | 62 |
| XR_300189.1 | 6 | - | 30515158 | 30570985 | 825 | 109 | 56 |
| XR_299486.1 | 6 | - | 30794471 | 30795802 | 930 | 1 | 1 |
| XR_299487.1 | 7 | - | 30794471 | 30795802 | 1036 | 1 | 2 |
| XR_297207.1 | 8 | - | 30829640 | 30835487 | 665 | 1 | 4 |
| XM_003128933.4 | 10 | - | 30988366 | 31134331 | 4147 | 10364 | 8083 |
| XM_005666604.1 | 4 | - | 30988366 | 31134331 | 4104 | 10307 | 8044 |
| XM_005666605.1 | 6 | - | 30988366 | 31134331 | 4140 | 10298 | 8043 |
| XM_005666606.1 | 6 | - | 30988366 | 31134331 | 4236 | 10313 | 8056 |
| XR_306767.1 | 8 | + | 31053371 | 31118866 | 1984 | 826 | 738 |
| XR_306768.1 | 8 | + | 31053371 | 31118866 | 1377 | 662 | 614 |
| XR_306769.1 | 8 | + | 31053371 | 31118866 | 1981 | 826 | 738 |
| XR_306770.1 | 8 | + | 31053371 | 31118866 | 1951 | 812 | 715 |
| XR_306771.1 | 17 | + | 31053371 | 31118866 | 1888 | 728 | 654 |
| XR_306772.1 | 17 | + | 31053371 | 31118866 | 1855 | 714 | 631 |
| XR_297817.1 | 17 | + | 31095622 | 31117368 | 285 | 0 | 4 |
| XM_005668038.1 | 7 | - | 31115794 | 31140539 | 824 | 243 | 246 |
| XR_298185.1 | 7 | - | 31133403 | 31139541 | 2091 | 1120 | 604 |
| XR_306773.1 | 7 | + | 31153614 | 31156339 | 605 | 9008 | 7510 |
| XR_299580.1 | 7 | - | 31217420 | 31238407 | 1091 | 125 | 115 |
| XR_300107.1 | 7 | - | 31234669 | 31237366 | 504 | 9 | 19 |
| XR_297786.1 | 8 | - | 31502990 | 31507746 | 1187 | 307 | 191 |
| XR_304407.1 | 9 | - | 31573751 | 31590905 | 1533 | 327 | 326 |
| XR_306512.1 | 4 | + | 32040435 | 32043762 | 787 | 6 | 14 |
| XR_305302.1 | 5 | + | 32205771 | 32310330 | 788 | 234 | 191 |
| XR_298451.1 | 7 | - | 32239216 | 32327027 | 268 | 0 | 1 |
| XR_309046.1 | 7 | - | 32278394 | 32644334 | 265 | 9 | 14 |
| XR_304410.1 | 7 | - | 32296077 | 32338320 | 1151 | 176 | 138 |
| XR_304411.1 | 10 | - | 32296077 | 32338320 | 1342 | 112 | 119 |
| XR_304412.1 | 10 | - | 32296077 | 32338320 | 733 | 160 | 119 |
| XR_116374.1 | 14 | + | 32594223 | 32604508 | 1446 | 1998 | 2088 |
| XR_308703.1 | 13 | + | 32594223 | 32604508 | 775 | 1338 | 1287 |
| XR_309047.1 | 6 | + | 32644445 | 32646659 | 1097 | 5846 | 2791 |
| XR_297389.1 | 9 | + | 32664743 | 32725492 | 650 | 0 | 3 |
| XR_297390.1 | 9 | + | 32664743 | 32725492 | 830 | 0 | 4 |
| XR_309543.1 | 13 | - | 32673830 | 32736934 | 1084 | 2 | 4 |
| XR_309544.1 | 5 | - | 32673830 | 32736934 | 1141 | 3 | 4 |
| XR_309545.1 | 7 | - | 32673830 | 32736934 | 1169 | 3 | 4 |
| XR_309546.1 | 2 | - | 32673830 | 32736934 | 1240 | 3 | 4 |
| XR_309547.1 | 2 | - | 32673830 | 32736934 | 1177 | 2 | 4 |
| XR_309542.1 | 2 | + | 32674898 | 32700045 | 587 | 1 | 3 |
| XR_309048.1 | 16 | - | 32727494 | 32749410 | 320 | 1 | 0 |
| XR_309049.1 | 16 | - | 32727494 | 32749410 | 351 | 1 | 3 |
| XR_304419.1 | 6 | + | 32738810 | 32755509 | 946 | 29 | 27 |
| XR_304420.1 | 6 | + | 32738810 | 32755509 | 838 | 28 | 25 |
| XR_304421.1 | 6 | + | 32738810 | 32755509 | 1023 | 22 | 26 |
| XR_304422.1 | 6 | + | 32738810 | 32755509 | 844 | 29 | 26 |
| XR_304423.1 | 6 | + | 32738810 | 32755509 | 736 | 28 | 24 |
| XR_304424.1 | 10 | + | 32738810 | 32755509 | 715 | 28 | 20 |
| XR_304425.1 | 11 | + | 32738810 | 32755509 | 613 | 28 | 19 |
| XR_305312.1 | 11 | - | 32809519 | 32811632 | 1545 | 1602 | 1725 |
| XR_308705.1 | 10 | + | 32952102 | 32955075 | 987 | 17 | 17 |
| XR_308706.1 | 6 | + | 32952102 | 32955075 | 849 | 14 | 12 |
| XR_309052.1 | 1 | - | 33068348 | 33181898 | 500 | 2 | 4 |
| XR_309053.1 | 1 | - | 33068348 | 33181898 | 518 | 1 | 3 |
| XR_309054.1 | 4 | - | 33068348 | 33181898 | 515 | 1 | 3 |
| XR_301711.1 | 4 | + | 33134045 | 33234797 | 929 | 10 | 9 |
| XR_301712.1 | 16 | + | 33134045 | 33234797 | 910 | 9 | 9 |
| XR_301713.1 | 7 | + | 33134045 | 33234797 | 916 | 10 | 9 |
| XR_301714.1 | 13 | + | 33134045 | 33234797 | 897 | 9 | 9 |
| XR_302209.1 | 1 | - | 33278316 | 33498829 | 282 | 4 | 3 |
| XR_305884.1 | 2 | + | 33279047 | 33313561 | 412 | 265 | 148 |
| XR_305885.1 | 2 | + | 33279047 | 33313561 | 409 | 328 | 164 |
| XR_305886.1 | 2 | + | 33279047 | 33313561 | 487 | 331 | 167 |
| XR_305887.1 | 5 | + | 33279047 | 33313561 | 544 | 265 | 149 |
| XR_305888.1 | X | + | 33279047 | 33313561 | 523 | 252 | 144 |
| XR_305889.1 | 7 | + | 33279047 | 33313561 | 405 | 250 | 138 |
| XR_308716.1 | 7 | - | 33648696 | 33657289 | 375 | 0 | 4 |
| XR_298187.1 | 9 | + | 33700062 | 33704706 | 531 | 2 | 3 |
| XR_308720.1 | 9 | + | 33897260 | 33907325 | 1202 | 8 | 14 |
| XR_308722.1 | 9 | + | 34164801 | 34205318 | 1167 | 47 | 31 |
| XR_303456.1 | 9 | - | 34216869 | 34426805 | 1276 | 137 | 132 |
| XR_303457.1 | 10 | - | 34216869 | 34426805 | 1684 | 194 | 170 |
| XR_302876.1 | 11 | - | 34328428 | 34332552 | 952 | 114 | 121 |
| XR_302877.1 | 2 | - | 34328428 | 34332552 | 742 | 78 | 75 |
| XR_297196.1 | 16 | + | 34554239 | 34602779 | 937 | 58 | 48 |
| XR_297197.1 | 3 | + | 34554239 | 34602779 | 808 | 78 | 95 |
| XR_297198.1 | 7 | + | 34554239 | 34602779 | 744 | 76 | 93 |
| XR_302878.1 | 7 | + | 34575600 | 34598807 | 658 | 12 | 9 |
| XR_297213.1 | 7 | - | 34616179 | 34617214 | 621 | 2 | 1 |
| XR_297214.1 | 17 | - | 34616179 | 34617214 | 501 | 2 | 0 |
| XR_308406.1 | 17 | + | 34858476 | 34874745 | 227 | 30 | 28 |
| XR_308408.1 | 17 | - | 35079464 | 35154971 | 248 | 5 | 6 |
| XR_308407.1 | 17 | + | 35165622 | 35228460 | 309 | 1 | 9 |
| XR_297848.1 | 8 | + | 35167885 | 35173855 | 441 | 0 | 14 |
| XR_309057.1 | 12 | - | 35246559 | 35334032 | 664 | 0 | 4 |
| XR_304437.1 | 1 | + | 35670703 | 35709921 | 568 | 0 | 3 |
| XR_304438.1 | 4 | - | 35710104 | 35712441 | 1122 | 8 | 19 |
| XR_302879.1 | 3 | + | 35787610 | 35921837 | 2513 | 47 | 46 |
| XR_302880.1 | 3 | + | 35787610 | 35921837 | 2478 | 48 | 45 |
| XR_302881.1 | 11 | + | 35787610 | 35921837 | 2339 | 40 | 41 |
| XR_305327.1 | 1 | + | 35839816 | 35872752 | 2159 | 479 | 366 |
| XR_305328.1 | 1 | + | 35839816 | 35872752 | 2566 | 472 | 346 |
| XR_305329.1 | 1 | + | 35839816 | 35872752 | 2004 | 454 | 349 |
| XR_116407.3 | 15 | - | 35982261 | 35994241 | 1848 | 99 | 59 |
| XR_309206.1 | 3 | - | 35982261 | 35994241 | 642 | 40 | 18 |
| XR_305333.1 | 11 | + | 36119956 | 36137176 | 1037 | 86 | 56 |
| XR_305334.1 | 13 | + | 36119956 | 36137176 | 996 | 81 | 52 |
| XR_305335.1 | 13 | + | 36119956 | 36137176 | 1007 | 81 | 52 |
| XR_306533.1 | 13 | + | 36174917 | 36308352 | 326 | 5 | 9 |
| XR_303775.1 | 13 | - | 36185632 | 36193090 | 663 | 0 | 1 |
| XR_308748.1 | 13 | + | 36325511 | 36333353 | 411 | 19 | 19 |
| XR_302888.1 | 13 | + | 36475929 | 36502189 | 493 | 1 | 4 |
| XR_308411.1 | 13 | + | 36802016 | 37035669 | 754 | 51 | 55 |
| XR_306817.1 | 13 | + | 37193512 | 37356237 | 1248 | 44 | 62 |
| XR_304835.1 | 15 | + | 37645924 | 37647431 | 478 | 2 | 3 |
| XR_304836.1 | 17 | + | 37645924 | 37647431 | 420 | 2 | 3 |
| XR_303782.1 | 11 | + | 37723761 | 37760531 | 725 | 145 | 120 |
| XM_005672869.1 | 11 | + | 37810113 | 37918963 | 5742 | 7191 | 6400 |
| XR_307983.1 | 11 | - | 37914300 | 37919520 | 1711 | 7 | 26 |
| XR_299491.1 | 7 | + | 38085414 | 38091866 | 475 | 2 | 2 |
| XR_299492.1 | 10 | - | 38096027 | 38099470 | 473 | 3 | 4 |
| XR_299493.1 | 9 | - | 38096027 | 38099470 | 608 | 3 | 7 |
| XR_300683.1 | 2 | - | 38170350 | 38278975 | 714 | 7 | 6 |
| XR_299630.1 | 16 | - | 38196817 | 38284617 | 2689 | 160 | 195 |
| XR_298324.1 | 16 | + | 38375098 | 38385908 | 270 | 0 | 2 |
| XR_308421.1 | 16 | + | 38519498 | 38522178 | 448 | 24 | 33 |
| XR_306229.1 | 3 | + | 38701524 | 38719375 | 512 | 17 | 21 |
| XR_306230.1 | 3 | + | 38701524 | 38719375 | 452 | 16 | 19 |
| XR_299135.1 | 3 | - | 38947746 | 38970385 | 716 | 92 | 113 |
| XR_304839.1 | 14 | - | 38964878 | 38982609 | 780 | 170 | 141 |
| XR_303472.1 | 1 | + | 39005049 | 39042155 | 501 | 6 | 4 |
| XR_299247.1 | 15 | - | 39273002 | 39305398 | 709 | 13 | 19 |
| XR_297824.1 | 6 | + | 39369213 | 39370893 | 700 | 12 | 19 |
| XR_299637.1 | 6 | + | 39374524 | 39438544 | 1196 | 98 | 75 |
| XR_299638.1 | 8 | + | 39374524 | 39438544 | 1280 | 114 | 90 |
| XR_299639.1 | 7 | + | 39374524 | 39438544 | 1259 | 100 | 88 |
| XR_299640.1 | 11 | + | 39374524 | 39438544 | 1140 | 96 | 76 |
| XR_299641.1 | 11 | + | 39374524 | 39438544 | 1192 | 98 | 75 |
| XR_299642.1 | 11 | + | 39374524 | 39438544 | 1168 | 98 | 75 |
| XR_299643.1 | 11 | + | 39374524 | 39438544 | 1108 | 96 | 75 |
| XR_299644.1 | 8 | + | 39374524 | 39438544 | 1052 | 20 | 16 |
| XR_299645.1 | 8 | + | 39374524 | 39438544 | 1146 | 96 | 72 |
| XR_299646.1 | 8 | + | 39374524 | 39438544 | 1087 | 96 | 72 |
| XR_299647.1 | 8 | + | 39374524 | 39438544 | 1105 | 98 | 74 |
| XR_299648.1 | 14 | + | 39374524 | 39438544 | 1073 | 97 | 72 |
| XR_303482.1 | 5 | - | 39480823 | 39485377 | 507 | 2 | 5 |
| XR_304844.1 | 5 | + | 39603068 | 39605041 | 315 | 465 | 513 |
| XR_298209.1 | 5 | - | 39621848 | 39626240 | 696 | 111 | 65 |
| XR_298207.1 | 5 | - | 39691087 | 39698882 | 2535 | 63 | 57 |
| XR_298208.1 | 13 | - | 39691087 | 39698882 | 2407 | 57 | 48 |
| XR_308425.1 | 2 | + | 39958613 | 40084728 | 1219 | 649 | 485 |
| XR_308426.1 | 16 | + | 39958613 | 40084728 | 1211 | 659 | 489 |
| XR_308427.1 | 16 | + | 39958613 | 40084728 | 1168 | 630 | 472 |
| XR_305344.1 | 17 | + | 40004670 | 40085792 | 827 | 68 | 145 |
| XR_300709.1 | 17 | + | 40022800 | 40089690 | 1378 | 2 | 5 |
| XR_307439.1 | 3 | + | 40286503 | 40310984 | 549 | 5 | 16 |
| XR_297826.1 | 9 | + | 40407955 | 40646779 | 730 | 3 | 4 |
| XR_298200.1 | 9 | - | 40551609 | 40556671 | 624 | 1513 | 1254 |
| XR_298201.1 | 3 | - | 40551609 | 40556671 | 728 | 1513 | 1255 |
| XR_309071.1 | 1 | - | 40564071 | 40568654 | 478 | 118 | 87 |
| XR_309072.1 | 14 | - | 40564071 | 40568654 | 782 | 122 | 79 |
| XR_309070.1 | 14 | + | 40569228 | 40597378 | 342 | 3 | 2 |
| XR_309210.1 | 15 | - | 40593019 | 40681904 | 462 | 100 | 118 |
| XR_301729.1 | 18 | - | 40609502 | 40617090 | 598 | 27 | 26 |
| XR_300710.1 | 14 | + | 40659251 | 40685914 | 254 | 7 | 36 |
| XR_306825.1 | 14 | - | 40768742 | 40934895 | 729 | 8 | 5 |
| XR_298756.1 | 15 | + | 40868637 | 40896475 | 627 | 27 | 26 |
| XR_307448.1 | 3 | - | 40925727 | 40943919 | 616 | 234 | 129 |
| XR_307449.1 | 3 | - | 40925727 | 40943919 | 657 | 236 | 133 |
| XR_307451.1 | 18 | - | 40943932 | 40950610 | 1470 | 729 | 502 |
| XR_307452.1 | 4 | - | 40943932 | 40950610 | 1539 | 734 | 502 |
| XR_308768.1 | 16 | - | 40973828 | 40978404 | 1881 | 7 | 16 |
| XR_308769.1 | 14 | - | 40973828 | 40978404 | 1789 | 7 | 14 |
| XR_308770.1 | 13 | - | 40973828 | 40978404 | 1739 | 7 | 13 |
| XR_302900.1 | 1 | - | 41094220 | 41289181 | 350 | 32 | 40 |
| XR_308774.1 | 8 | - | 41464070 | 41476914 | 2834 | 5456 | 4105 |
| XR_305353.1 | 11 | + | 41481141 | 41487339 | 929 | 52 | 44 |
| XR_305354.1 | 3 | + | 41481141 | 41487339 | 949 | 52 | 44 |
| XR_308778.1 | 17 | + | 41490109 | 41496322 | 1206 | 1695 | 1038 |
| XR_308779.1 | 1 | + | 41490109 | 41496322 | 1187 | 1781 | 1093 |
| XR_308780.1 | 1 | + | 41490109 | 41496322 | 1105 | 1672 | 1019 |
| XR_308781.1 | 1 | + | 41490109 | 41496322 | 1095 | 1651 | 999 |
| XR_308782.1 | 1 | + | 41490109 | 41496322 | 1162 | 1653 | 1011 |
| XR_306559.1 | 2 | + | 41501716 | 41506075 | 457 | 0 | 3 |
| XR_303484.1 | 2 | + | 41819274 | 41823070 | 1308 | 2 | 3 |
| XR_303485.1 | 5 | + | 41819274 | 41823070 | 1233 | 2 | 3 |
| XM_005667361.1 | 4 | - | 41840596 | 41858037 | 665 | 2 | 10 |
| XR_305358.1 | Y | - | 42017643 | 42027282 | 1416 | 7048 | 3313 |
| XR_304461.1 | 9 | - | 42147746 | 42151898 | 319 | 1 | 1 |
| XR_305912.1 | 9 | + | 42258834 | 42266966 | 500 | 1 | 6 |
| XR_303487.1 | 9 | + | 42299629 | 42314018 | 738 | 34 | 39 |
| XR_303488.1 | 14 | + | 42299629 | 42314018 | 651 | 29 | 39 |
| XR_298204.1 | 14 | - | 42338547 | 42352803 | 2751 | 1196 | 1136 |
| XR_298205.1 | 4 | - | 42338547 | 42352803 | 1689 | 692 | 689 |
| XR_298206.1 | 4 | - | 42338547 | 42352803 | 2072 | 297 | 349 |
| XR_302257.1 | 4 | + | 42859275 | 42861361 | 454 | 40 | 64 |
| XR_302908.1 | 2 | + | 42985572 | 43254877 | 397 | 10 | 12 |
| XR_297394.1 | 2 | + | 43086118 | 43089178 | 1022 | 21 | 16 |
| XR_303490.1 | 9 | - | 43223103 | 43289792 | 408 | 17 | 21 |
| XR_304464.1 | 16 | - | 43373637 | 43571135 | 391 | 10 | 16 |
| XR_300114.1 | 13 | - | 43510250 | 43526127 | 1927 | 37 | 20 |
| XR_300115.1 | 13 | - | 43510250 | 43526127 | 1726 | 35 | 18 |
| XR_305365.1 | 1 | - | 43666930 | 44009747 | 875 | 10 | 13 |
| XR_305366.1 | 1 | - | 43666930 | 44009747 | 989 | 10 | 14 |
| XR_305367.1 | 3 | - | 43666930 | 44009747 | 1022 | 13 | 13 |
| XR_305368.1 | 8 | - | 43666930 | 44009747 | 862 | 7 | 12 |
| XR_309082.1 | 9 | - | 43667473 | 43692059 | 1059 | 128 | 207 |
| XR_300087.1 | 10 | - | 43707934 | 43716571 | 378 | 0 | 1 |
| XR_304466.1 | 6 | + | 43795680 | 43806995 | 312 | 1 | 2 |
| XR_307476.1 | 1 | + | 44113876 | 44114551 | 561 | 23 | 18 |
| XR_307477.1 | 15 | + | 44113876 | 44114551 | 556 | 23 | 18 |
| XM_003482387.2 | 15 | - | 44121473 | 44124508 | 703 | 10 | 11 |
| XR_307478.1 | 2 | + | 44233563 | 44254151 | 1784 | 17 | 17 |
| XR_307479.1 | 16 | + | 44233563 | 44254151 | 1496 | 14 | 12 |
| XR_309084.1 | 16 | + | 44269223 | 44324746 | 747 | 0 | 9 |
| XR_309085.1 | 5 | + | 44269223 | 44324746 | 665 | 0 | 8 |
| XR_309086.1 | 5 | + | 44269223 | 44324746 | 524 | 0 | 8 |
| XR_309087.1 | 4 | + | 44269223 | 44324746 | 638 | 0 | 8 |
| XR_309088.1 | 4 | + | 44269223 | 44324746 | 850 | 0 | 12 |
| XR_309089.1 | 1 | + | 44269223 | 44324746 | 556 | 0 | 7 |
| XM_005664492.1 | 8 | + | 44473636 | 44498597 | 3542 | 13 | 31 |
| XR_306232.1 | 8 | - | 44807715 | 44816252 | 733 | 2 | 16 |
| XR_299251.1 | 8 | + | 44848642 | 44857848 | 540 | 53 | 42 |
| XR_299252.1 | 10 | + | 44848642 | 44857848 | 1025 | 388 | 290 |
| XR_299253.1 | 10 | + | 44848642 | 44857848 | 536 | 64 | 43 |
| XM_003359466.2 | 12 | - | 44893544 | 44917836 | 1134 | 311 | 246 |
| XM_005671684.1 | 12 | - | 44893544 | 44917836 | 1243 | 394 | 334 |
| XM_005671685.1 | 12 | - | 44893544 | 44917836 | 1432 | 479 | 464 |
| XM_005671686.1 | 12 | - | 44893544 | 44917836 | 1271 | 394 | 334 |
| XM_005671687.1 | 12 | - | 44893544 | 44917836 | 1253 | 394 | 334 |
| XM_005671688.1 | 12 | - | 44893544 | 44917836 | 1068 | 306 | 240 |
| XM_005671689.1 | 12 | - | 44893544 | 44917836 | 1283 | 308 | 240 |
| XR_308002.1 | 12 | - | 44893544 | 44917836 | 944 | 280 | 215 |
| XR_300716.1 | 1 | - | 45063293 | 45084132 | 700 | 2 | 2 |
| XR_300717.1 | 1 | - | 45106872 | 45128389 | 1076 | 68 | 53 |
| XR_300718.1 | 1 | - | 45106872 | 45128389 | 1073 | 68 | 53 |
| XR_300719.1 | 14 | - | 45106872 | 45128389 | 1040 | 69 | 53 |
| XR_300720.1 | 4 | - | 45106872 | 45128389 | 1279 | 68 | 56 |
| XR_300721.1 | 7 | - | 45106872 | 45128389 | 1034 | 68 | 53 |
| XR_303806.1 | 7 | - | 45130026 | 45135203 | 548 | 2 | 4 |
| XR_303807.1 | 7 | - | 45130026 | 45135203 | 683 | 2 | 8 |
| XR_303808.1 | 6 | - | 45130026 | 45135203 | 469 | 2 | 5 |
| XM_005658718.1 | 13 | + | 45325741 | 45449550 | 1264 | 8 | 6 |
| XR_309093.1 | 13 | - | 45384466 | 45388260 | 1762 | 8 | 18 |
| XR_297589.1 | 16 | - | 45386000 | 45390421 | 272 | 12 | 13 |
| XR_299259.1 | 16 | + | 45549295 | 45593048 | 719 | 5 | 5 |
| XR_299260.1 | 6 | + | 45549295 | 45593048 | 820 | 5 | 5 |
| XR_299261.1 | 14 | + | 45549295 | 45593048 | 698 | 5 | 5 |
| XR_299262.1 | 2 | + | 45549295 | 45593048 | 640 | 5 | 5 |
| XR_299263.1 | 3 | + | 45549295 | 45593048 | 792 | 5 | 6 |
| XR_303815.1 | 5 | + | 45656580 | 45679976 | 291 | 6 | 4 |
| XR_303813.1 | 1 | - | 45666491 | 45694658 | 467 | 4 | 6 |
| XR_303814.1 | 1 | - | 45666491 | 45694658 | 352 | 3 | 6 |
| XR_297546.1 | 8 | - | 45686221 | 45706772 | 472 | 1760 | 1315 |
| XR_309215.1 | 3 | - | 45687396 | 45757972 | 1792 | 9 | 12 |
| XM_003135123.2 | 3 | + | 45721918 | 45731594 | 541 | 1 | 2 |
| XM_005673625.1 | 6 | + | 45721918 | 45731594 | 511 | 1 | 2 |
| XR_115866.2 | 5 | + | 45793437 | 45807328 | 2172 | 1718 | 2110 |
| XR_302918.1 | 18 | + | 45793437 | 45807328 | 2180 | 1823 | 2219 |
| XR_309220.1 | 3 | + | 45856343 | 45880197 | 2040 | 49 | 22 |
| XR_309221.1 | 9 | + | 45920043 | 45939730 | 1061 | 78 | 66 |
| XR_297851.1 | 1 | - | 45958959 | 46118914 | 776 | 99 | 53 |
| XR_297852.1 | 13 | - | 45958959 | 46118914 | 866 | 102 | 53 |
| XR_297853.1 | 6 | - | 45958959 | 46118914 | 762 | 109 | 52 |
| XR_297854.1 | 18 | - | 45958959 | 46118914 | 684 | 103 | 51 |
| XR_306839.1 | 3 | + | 46075664 | 46103035 | 2112 | 472 | 491 |
| XR_306840.1 | 6 | + | 46075664 | 46103035 | 2092 | 470 | 490 |
| XR_306841.1 | 6 | + | 46075664 | 46103035 | 2051 | 461 | 477 |
| XR_306842.1 | 15 | + | 46075664 | 46103035 | 2017 | 461 | 477 |
| XR_300232.1 | 2 | - | 46118963 | 46137466 | 294 | 1 | 0 |
| XR_303827.1 | 9 | + | 46363776 | 46376626 | 787 | 154 | 149 |
| XR_309113.1 | 9 | + | 46513323 | 46692485 | 599 | 25 | 23 |
| XR_306845.1 | 9 | - | 46584783 | 46668737 | 1415 | 3 | 12 |
| XR_305384.1 | 6 | + | 46622197 | 46638290 | 429 | 3 | 2 |
| XR_305385.1 | 2 | + | 46622197 | 46638290 | 411 | 2 | 2 |
| XR_305386.1 | 4 | + | 46622197 | 46638290 | 389 | 3 | 2 |
| XR_308449.1 | 4 | - | 46660481 | 46938008 | 953 | 83 | 207 |
| XR_308450.1 | 4 | - | 46660481 | 46938008 | 1019 | 92 | 215 |
| XR_308451.1 | 4 | - | 46660481 | 46938008 | 864 | 79 | 201 |
| XR_305387.1 | 4 | + | 46662307 | 46728352 | 826 | 7 | 9 |
| XR_309117.1 | 15 | - | 46666111 | 46986756 | 496 | 0 | 3 |
| XR_309118.1 | 15 | - | 46666111 | 46986756 | 433 | 0 | 3 |
| XR_309119.1 | 3 | - | 46666111 | 46986756 | 544 | 3 | 3 |
| XR_309120.1 | 3 | - | 46666111 | 46986756 | 436 | 0 | 3 |
| XR_309121.1 | 6 | - | 46666111 | 46986756 | 490 | 0 | 3 |
| XR_309122.1 | 3 | - | 46666111 | 46986756 | 401 | 1 | 3 |
| XR_309123.1 | 5 | - | 46666111 | 46986756 | 473 | 3 | 4 |
| XR_297252.1 | 10 | + | 46669673 | 46770489 | 399 | 6 | 9 |
| XR_303829.1 | 13 | + | 46707828 | 46709227 | 547 | 8 | 30 |
| XR_308806.1 | 2 | - | 46768005 | 46816635 | 1009 | 7 | 12 |
| XR_308807.1 | 4 | - | 46768005 | 46816635 | 1070 | 7 | 14 |
| XR_308808.1 | 17 | - | 46768005 | 46816635 | 1265 | 9 | 13 |
| XR_308809.1 | 17 | - | 46768005 | 46816635 | 1050 | 7 | 11 |
| XR_305388.1 | 9 | - | 46779332 | 46782420 | 832 | 10 | 9 |
| XR_305389.1 | 9 | - | 46779332 | 46782420 | 605 | 9 | 7 |
| XR_297679.1 | 7 | - | 47030580 | 47031320 | 349 | 44 | 40 |
| XR_308454.1 | 7 | - | 47053285 | 47195109 | 339 | 1 | 4 |
| XR_308455.1 | 7 | - | 47053285 | 47195109 | 493 | 1 | 5 |
| XR_304855.1 | 7 | + | 47097359 | 47112734 | 1133 | 869 | 893 |
| XR_304856.1 | 12 | + | 47097359 | 47112734 | 1130 | 869 | 893 |
| XR_303838.1 | 6 | - | 47209647 | 47223857 | 3172 | 1020 | 1026 |
| XR_308456.1 | 3 | - | 47244827 | 47262186 | 701 | 8 | 8 |
| XR_301761.1 | 1 | + | 47321961 | 47336697 | 640 | 3 | 4 |
| XR_308458.1 | 1 | - | 47364244 | 47386346 | 836 | 66 | 52 |
| XR_308459.1 | 1 | - | 47364244 | 47386346 | 699 | 64 | 53 |
| XR_308810.1 | 1 | - | 47556634 | 47558241 | 553 | 8 | 20 |
| XR_308811.1 | 1 | - | 47556634 | 47558241 | 610 | 8 | 20 |
| XR_308015.1 | 14 | - | 47808712 | 47815636 | 1177 | 127 | 159 |
| XR_299472.1 | 15 | - | 47963617 | 47981594 | 2195 | 187 | 123 |
| XR_305399.1 | 17 | - | 48272279 | 48357083 | 802 | 84 | 88 |
| XR_305400.1 | 11 | - | 48272279 | 48357083 | 854 | 87 | 81 |
| XR_305401.1 | 1 | - | 48272279 | 48357083 | 865 | 80 | 84 |
| XR_305402.1 | 16 | - | 48272279 | 48357083 | 929 | 85 | 92 |
| XR_305403.1 | 4 | - | 48272279 | 48357083 | 736 | 81 | 87 |
| XR_305404.1 | 9 | - | 48272279 | 48357083 | 797 | 77 | 83 |
| XR_305405.1 | 9 | - | 48272279 | 48357083 | 626 | 50 | 64 |
| XR_308826.1 | 13 | - | 48598943 | 48616947 | 301 | 2 | 1 |
| XR_298562.1 | 2 | + | 48713184 | 48722160 | 1507 | 219 | 166 |
| XR_297149.1 | 1 | + | 49035031 | 49049259 | 444 | 0 | 6 |
| XR_298764.1 | 4 | - | 49099838 | 49150374 | 2450 | 37 | 103 |
| XR_301768.1 | 4 | + | 49231953 | 49234725 | 2103 | 61 | 87 |
| XR_130489.2 | 4 | - | 49463080 | 49469094 | 1223 | 565 | 433 |
| XR_130490.2 | 9 | - | 49463080 | 49469094 | 1504 | 685 | 515 |
| XR_306257.1 | 9 | - | 49496916 | 49536526 | 858 | 2 | 2 |
| XR_308476.1 | 12 | - | 49556779 | 49558025 | 597 | 2 | 6 |
| XR_307524.1 | 9 | - | 49661621 | 49671796 | 999 | 488 | 311 |
| XR_307525.1 | 5 | - | 49661621 | 49671796 | 1026 | 489 | 309 |
| XR_299136.1 | 5 | - | 50174100 | 50298199 | 394 | 2 | 6 |
| XR_309126.1 | 5 | + | 50185320 | 50191520 | 657 | 2 | 0 |
| XR_299137.1 | 7 | + | 50262382 | 50268897 | 1015 | 3 | 8 |
| XR_302924.1 | 17 | + | 50332475 | 50335496 | 751 | 353 | 276 |
| XR_299139.1 | 17 | - | 50361604 | 50365372 | 1562 | 154 | 210 |
| XR_299551.1 | 17 | + | 50421309 | 50438341 | 2346 | 1304 | 984 |
| XR_309130.1 | 9 | + | 50450512 | 50463100 | 622 | 4076 | 2615 |
| XR_309131.1 | 9 | + | 50450512 | 50463100 | 773 | 4231 | 2707 |
| XR_309132.1 | 9 | + | 50450512 | 50463100 | 741 | 4048 | 2614 |
| XR_309133.1 | 9 | + | 50450512 | 50463100 | 594 | 3893 | 2522 |
| XR_309134.1 | 9 | + | 50450512 | 50463100 | 585 | 3778 | 2436 |
| XR_299553.1 | 9 | + | 50503639 | 50734470 | 3185 | 541 | 401 |
| XR_298578.1 | 7 | + | 50569482 | 50576824 | 651 | 72 | 76 |
| XR_298582.1 | 8 | - | 50766518 | 50770096 | 1217 | 195 | 118 |
| XR_300503.1 | 12 | + | 50958841 | 50963160 | 721 | 2944 | 3461 |
| XR_300504.1 | 12 | + | 50958841 | 50963160 | 739 | 2974 | 3485 |
| XR_299556.1 | 12 | + | 51138446 | 51152999 | 1118 | 40 | 32 |
| XR_299557.1 | 12 | + | 51138446 | 51152999 | 1238 | 41 | 32 |
| XR_299558.1 | 12 | + | 51138446 | 51152999 | 1071 | 39 | 30 |
| XR_299561.1 | 17 | - | 51432214 | 51457882 | 1543 | 132 | 101 |
| XR_305944.1 | 5 | - | 51647169 | 51677190 | 760 | 3 | 1 |
| XR_308843.1 | 3 | + | 51698126 | 51712510 | 1196 | 20 | 11 |
| XR_308844.1 | 6 | + | 51698126 | 51712510 | 1192 | 20 | 11 |
| XR_308845.1 | 6 | + | 51698126 | 51712510 | 1506 | 315 | 310 |
| XR_308846.1 | 7 | + | 51698126 | 51712510 | 1113 | 20 | 11 |
| XR_300566.1 | 6 | + | 51727662 | 51729662 | 1292 | 35 | 34 |
| XR_306854.1 | 9 | + | 51825271 | 52224301 | 214 | 0 | 4 |
| XR_306270.1 | 9 | - | 51862538 | 51867308 | 958 | 5 | 5 |
| XR_308848.1 | 9 | - | 51926509 | 51930179 | 507 | 6 | 6 |
| XM_003131920.2 | 1 | + | 51999504 | 52016592 | 1779 | 28 | 31 |
| XM_005669168.1 | 16 | + | 51999504 | 52016592 | 1773 | 28 | 31 |
| XR_308854.1 | 16 | + | 52115945 | 52139269 | 605 | 12 | 26 |
| XR_304474.1 | 17 | - | 52194114 | 52202650 | 554 | 89 | 105 |
| XR_298459.1 | 4 | + | 52216598 | 52225599 | 903 | 4 | 10 |
| XR_298460.1 | 4 | + | 52216598 | 52225599 | 654 | 2 | 1 |
| XR_303848.1 | 1 | + | 52234124 | 52242805 | 503 | 19 | 5 |
| XM_003133379.3 | 1 | + | 52416082 | 52528692 | 1999 | 123 | 124 |
| XR_306602.1 | 1 | + | 52420175 | 52424455 | 950 | 4893 | 3402 |
| XR_303849.1 | 1 | + | 52422376 | 52429103 | 554 | 1 | 3 |
| XR_303850.1 | 1 | + | 52422376 | 52429103 | 353 | 1 | 3 |
| XR_305947.1 | 1 | - | 52426076 | 52479834 | 2678 | 7 | 40 |
| XR_306601.1 | 1 | - | 52442479 | 52447953 | 931 | 7 | 15 |
| XR_308036.1 | 1 | + | 52575634 | 52583504 | 832 | 16 | 23 |
| XR_308037.1 | 1 | + | 52575634 | 52583504 | 672 | 14 | 23 |
| XR_302328.1 | 1 | - | 52667219 | 52699961 | 248 | 1 | 3 |
| XR_308857.1 | 1 | + | 52682410 | 52723182 | 1489 | 12 | 37 |
| XR_308858.1 | 12 | + | 52682410 | 52723182 | 1666 | 13 | 40 |
| XR_308859.1 | 17 | + | 52682410 | 52723182 | 1688 | 14 | 42 |
| XR_308860.1 | 6 | + | 52682410 | 52723182 | 1563 | 11 | 40 |
| XR_308861.1 | 7 | + | 52682410 | 52723182 | 1460 | 12 | 37 |
| XR_308862.1 | 7 | + | 52682410 | 52723182 | 1496 | 13 | 37 |
| XR_308863.1 | 8 | + | 52682410 | 52723182 | 1393 | 11 | 37 |
| XR_299565.1 | 8 | + | 52781826 | 52791008 | 840 | 59 | 100 |
| XR_299566.1 | 8 | + | 52781826 | 52791008 | 957 | 54 | 98 |
| XR_299567.1 | 8 | + | 52826736 | 52836219 | 545 | 2 | 9 |
| XM_005667483.1 | 9 | + | 52858504 | 52861012 | 1195 | 28 | 32 |
| XR_305437.1 | 13 | + | 52858504 | 52861012 | 1274 | 30 | 31 |
| XR_309150.1 | 13 | - | 52876628 | 52877202 | 342 | 6 | 3 |
| XM_003482642.2 | 13 | + | 52945921 | 53008839 | 1828 | 316 | 276 |
| XR_305439.1 | 13 | + | 52945921 | 53008839 | 1897 | 452 | 315 |
| XR_299202.1 | 13 | + | 53099767 | 53140867 | 574 | 1 | 1 |
| XR_299267.1 | 13 | - | 53119241 | 53136246 | 1058 | 238 | 158 |
| XR_308864.1 | 13 | + | 53146342 | 53150019 | 1474 | 41 | 68 |
| XR_299268.1 | 5 | + | 53166108 | 53176970 | 1604 | 358 | 311 |
| XR_308482.1 | 6 | + | 53279221 | 53328367 | 275 | 0 | 7 |
| XR_305955.1 | 9 | - | 53325974 | 53329961 | 929 | 48 | 51 |
| XR_305956.1 | 9 | - | 53325974 | 53329961 | 1144 | 40 | 45 |
| XR_305957.1 | 3 | - | 53325974 | 53329961 | 1227 | 40 | 45 |
| XR_302330.1 | 3 | + | 53500633 | 53505018 | 1138 | 4 | 17 |
| XR_308866.1 | 16 | - | 53502771 | 53526274 | 1151 | 2 | 11 |
| XR_299273.1 | 16 | - | 53541661 | 53547921 | 928 | 99 | 75 |
| XM_005664649.1 | 16 | - | 53545796 | 53551238 | 737 | 4 | 14 |
| XR_304874.1 | 5 | - | 53576927 | 53637383 | 829 | 7 | 11 |
| XR_302331.1 | 4 | + | 53647051 | 53668630 | 766 | 20 | 37 |
| XR_304870.1 | 4 | + | 53691001 | 53739084 | 1061 | 75 | 70 |
| XR_304871.1 | 4 | + | 53691001 | 53739084 | 1058 | 75 | 69 |
| XR_304872.1 | 4 | + | 53691001 | 53739084 | 475 | 21 | 28 |
| XR_304873.1 | 4 | + | 53691001 | 53739084 | 1168 | 76 | 70 |
| XR_308868.1 | 4 | + | 53705171 | 53792503 | 205 | 1 | 3 |
| XM_005658926.1 | 4 | - | 53890649 | 53907697 | 690 | 11 | 9 |
| XR_300741.1 | 4 | - | 54015929 | 54400999 | 546 | 20 | 21 |
| XR_303874.1 | 4 | + | 54053888 | 54062758 | 1576 | 1335 | 1060 |
| XR_309164.1 | 4 | + | 54435901 | 54470796 | 971 | 8 | 18 |
| XR_297760.1 | 4 | - | 54538200 | 54541569 | 2069 | 218 | 420 |
| XR_297761.1 | 4 | - | 54538200 | 54541569 | 2308 | 238 | 469 |
| XR_298461.1 | 1 | + | 54732645 | 54736059 | 963 | 4 | 13 |
| XR_308880.1 | 1 | - | 54753781 | 54757216 | 1278 | 7 | 14 |
| XR_309165.1 | 1 | + | 54795042 | 54799900 | 1818 | 23 | 29 |
| XR_304889.1 | 1 | - | 54950106 | 54963084 | 1071 | 2 | 14 |
| XR_304888.1 | 1 | + | 55057653 | 55113615 | 369 | 17 | 24 |
| XR_300753.1 | 1 | - | 55126495 | 55268553 | 208 | 4 | 4 |
| XR_306274.1 | 1 | - | 55393361 | 55444294 | 334 | 0 | 2 |
| XR_298223.1 | 7 | + | 55490155 | 55507770 | 594 | 5 | 3 |
| XR_298224.1 | 7 | + | 55490155 | 55507770 | 522 | 2 | 1 |
| XR_306275.1 | 7 | - | 55596534 | 55696459 | 586 | 0 | 3 |
| XR_309167.1 | 7 | + | 55606026 | 55611420 | 877 | 19 | 36 |
| XR_115980.3 | 7 | - | 55927357 | 55929995 | 1649 | 71 | 73 |
| XR_303889.1 | 7 | + | 56034838 | 56068799 | 1509 | 2 | 21 |
| XR_305962.1 | 8 | + | 56063320 | 56077085 | 1152 | 42 | 47 |
| XR_302930.1 | 10 | + | 56080088 | 56085720 | 930 | 15 | 16 |
| XR_304879.1 | 7 | + | 56140904 | 56149530 | 1639 | 55 | 72 |
| XR_304880.1 | 18 | + | 56140904 | 56149530 | 1524 | 54 | 69 |
| XR_304881.1 | 2 | + | 56140904 | 56149530 | 2073 | 75 | 88 |
| XR_304882.1 | 2 | + | 56140904 | 56149530 | 1570 | 54 | 70 |
| XR_305453.1 | 2 | + | 56176955 | 56180614 | 1377 | 283 | 291 |
| XR_305454.1 | 3 | + | 56176955 | 56180614 | 1550 | 300 | 317 |
| XR_305455.1 | 7 | + | 56176955 | 56180614 | 846 | 214 | 227 |
| XR_307540.1 | 7 | - | 56242424 | 56246887 | 670 | 4 | 16 |
| XR_307541.1 | 13 | + | 56255862 | 56266692 | 1071 | 5 | 12 |
| XR_305965.1 | 13 | + | 56262075 | 56290224 | 1263 | 2990 | 1633 |
| XR_308055.1 | 13 | + | 56282808 | 56366021 | 1116 | 1495 | 553 |
| XR_306619.1 | 13 | - | 56328970 | 56330563 | 534 | 44 | 97 |
| XR_307542.1 | 13 | - | 56461225 | 56525356 | 1050 | 56 | 37 |
| XR_308890.1 | 1 | + | 57195504 | 57201642 | 1271 | 11 | 3 |
| XR_308891.1 | 1 | + | 57195504 | 57201642 | 1274 | 11 | 3 |
| XR_308892.1 | 14 | + | 57195504 | 57201642 | 1237 | 11 | 3 |
| XR_308893.1 | 18 | + | 57195504 | 57201642 | 1191 | 9 | 4 |
| XR_308894.1 | 3 | + | 57195504 | 57201642 | 1194 | 9 | 4 |
| XR_308895.1 | 3 | + | 57195504 | 57201642 | 1271 | 11 | 3 |
| XR_308896.1 | 6 | + | 57195504 | 57201642 | 1332 | 13 | 3 |
| XR_308897.1 | 8 | + | 57195504 | 57201642 | 1205 | 11 | 3 |
| XR_309526.1 | 2 | + | 57373952 | 57382121 | 730 | 1 | 6 |
| XR_297312.1 | 18 | + | 57387094 | 57411477 | 830 | 84 | 83 |
| XR_297313.1 | 14 | + | 57387094 | 57411477 | 705 | 86 | 82 |
| XM_005652711.1 | 13 | + | 57543794 | 57572299 | 973 | 213 | 232 |
| XM_005652712.1 | 15 | + | 57543794 | 57572299 | 900 | 154 | 192 |
| XR_306281.1 | 4 | + | 57819075 | 57878071 | 700 | 7 | 3 |
| XR_306282.1 | 5 | + | 57819075 | 57878071 | 632 | 7 | 3 |
| XR_297289.1 | 5 | - | 57848401 | 57899465 | 743 | 114 | 109 |
| XR_297290.1 | 7 | - | 57848401 | 57899465 | 748 | 109 | 106 |
| XR_304477.1 | 7 | + | 57889396 | 57901860 | 2939 | 9 | 39 |
| XR_308900.1 | 11 | + | 57898613 | 57904998 | 594 | 1 | 1 |
| XR_304481.1 | 12 | + | 58268457 | 58279665 | 844 | 239 | 211 |
| XR_300762.1 | 6 | + | 58446821 | 58458325 | 1103 | 7 | 10 |
| NR_045043.1 | 7 | + | 58801130 | 58803241 | 1597 | 21 | 28 |
| XR_305983.1 | 6 | + | 58816329 | 58884026 | 868 | 4 | 11 |
| XR_305984.1 | 6 | + | 58816329 | 58884026 | 734 | 4 | 11 |
| XR_306286.1 | 14 | + | 58941913 | 59180271 | 736 | 2 | 4 |
| XR_306287.1 | 14 | + | 58941913 | 59180271 | 856 | 2 | 5 |
| XR_306288.1 | 14 | + | 58941913 | 59180271 | 860 | 2 | 5 |
| XR_306289.1 | 14 | + | 58941913 | 59180271 | 643 | 2 | 3 |
| XR_306290.1 | 5 | + | 58941913 | 59180271 | 661 | 2 | 4 |
| XR_297705.1 | 5 | + | 59158071 | 59181467 | 797 | 100 | 79 |
| XR_308914.1 | 4 | - | 59214102 | 59233283 | 1368 | 1 | 10 |
| XR_308915.1 | 2 | - | 59214102 | 59233283 | 1381 | 2 | 8 |
| XR_304485.1 | 12 | + | 59221849 | 59235214 | 2389 | 2398 | 974 |
| XR_297706.1 | 7 | + | 59222571 | 59246397 | 631 | 63 | 28 |
| XR_308916.1 | 11 | + | 59233310 | 59252214 | 1968 | 8 | 12 |
| XR_304899.1 | 14 | + | 59270974 | 59437784 | 910 | 2 | 13 |
| XR_304488.1 | 14 | + | 59412344 | 59423429 | 7139 | 420 | 435 |
| XR_304489.1 | 13 | + | 59412344 | 59423429 | 7832 | 588 | 644 |
| XR_309237.1 | 13 | - | 59544249 | 59608630 | 3914 | 1748 | 1624 |
| XR_309238.1 | 18 | - | 59544249 | 59608630 | 3815 | 1712 | 1597 |
| XR_309239.1 | 15 | - | 59544249 | 59608630 | 3349 | 1416 | 1319 |
| XR_298235.1 | 15 | - | 59735560 | 59757424 | 470 | 3 | 2 |
| XM_005669259.1 | 4 | + | 59959841 | 59960458 | 879 | 103 | 93 |
| XR_306640.1 | 13 | + | 59959841 | 59960458 | 966 | 103 | 98 |
| XR_302933.1 | 13 | - | 60048446 | 60151061 | 1185 | 1 | 8 |
| XR_307552.1 | 13 | - | 60348969 | 60353327 | 616 | 11 | 12 |
| XR_307553.1 | 8 | - | 60348969 | 60353327 | 847 | 5 | 13 |
| XR_303895.1 | 15 | + | 60458015 | 60460130 | 293 | 1 | 1 |
| XR_306291.1 | 9 | - | 60471862 | 60534530 | 259 | 0 | 5 |
| XR_309388.1 | 18 | - | 60507961 | 60532358 | 751 | 2 | 3 |
| XR_307554.1 | 14 | + | 60528629 | 60599808 | 1202 | 16 | 26 |
| XR_304498.1 | 14 | - | 60884241 | 60971721 | 1637 | 117 | 102 |
| XR_297088.1 | 14 | - | 60929545 | 61061162 | 466 | 2 | 8 |
| XR_301790.1 | 4 | - | 60982831 | 60984068 | 408 | 13 | 11 |
| XR_301791.1 | 4 | - | 60982831 | 60984068 | 408 | 13 | 11 |
| XR_297086.1 | 3 | - | 61114839 | 61130394 | 687 | 1 | 5 |
| XR_297087.1 | 18 | - | 61114839 | 61130394 | 508 | 1 | 4 |
| XR_302934.1 | 18 | + | 61285006 | 61352062 | 1333 | 3 | 8 |
| XR_302935.1 | 4 | + | 61285006 | 61352062 | 1329 | 3 | 8 |
| XR_302936.1 | 4 | + | 61285006 | 61352062 | 1207 | 3 | 8 |
| XR_302937.1 | 4 | + | 61285006 | 61352062 | 1348 | 6 | 9 |
| XR_302938.1 | 4 | + | 61285006 | 61352062 | 1331 | 3 | 8 |
| XM_005673775.1 | 4 | - | 61531171 | 61562920 | 2520 | 21 | 20 |
| XR_304501.1 | 4 | + | 61614233 | 61627810 | 516 | 14 | 5 |
| XR_307560.1 | 4 | + | 61667986 | 61678075 | 397 | 11 | 11 |
| XR_307561.1 | 4 | + | 61667986 | 61678075 | 360 | 10 | 10 |
| XR_135407.2 | 7 | - | 61684775 | 61688742 | 980 | 86 | 64 |
| XR_307562.1 | 7 | - | 61684775 | 61688742 | 892 | 70 | 53 |
| XR_306888.1 | 16 | + | 61955215 | 61969756 | 829 | 0 | 7 |
| XR_304502.1 | 17 | - | 62014291 | 62201254 | 660 | 1 | 5 |
| XR_304503.1 | 17 | - | 62014291 | 62201254 | 684 | 1 | 7 |
| XR_304504.1 | 15 | - | 62014291 | 62201254 | 550 | 1 | 6 |
| XR_304505.1 | 9 | - | 62014291 | 62201254 | 740 | 1 | 5 |
| XR_304506.1 | 14 | - | 62014291 | 62201254 | 612 | 1 | 6 |
| XR_303905.1 | 14 | + | 62140428 | 62157608 | 1837 | 31 | 48 |
| XR_303906.1 | 16 | - | 62185386 | 62190995 | 1949 | 152 | 181 |
| XR_303910.1 | 12 | - | 62653075 | 62667628 | 1176 | 2026 | 1711 |
| XR_303911.1 | 11 | - | 62653075 | 62667628 | 1174 | 2024 | 1709 |
| XR_303912.1 | 6 | - | 62653075 | 62667628 | 1130 | 1899 | 1601 |
| XR_303915.1 | 13 | - | 62921420 | 62925969 | 1000 | 2 | 1 |
| XR_303919.1 | 13 | - | 62979460 | 62981249 | 458 | 64 | 38 |
| XR_308531.1 | 13 | - | 63133343 | 63182128 | 1321 | 501 | 563 |
| XR_300772.1 | 13 | - | 63279427 | 63296921 | 620 | 1 | 2 |
| XR_304512.1 | 13 | + | 63494069 | 63539399 | 436 | 3 | 1 |
| XR_304513.1 | 13 | + | 63494069 | 63539399 | 454 | 2 | 1 |
| XR_304514.1 | 14 | + | 63494069 | 63539399 | 382 | 2 | 1 |
| XR_307563.1 | 14 | + | 63531063 | 63535922 | 413 | 42 | 35 |
| XR_297164.1 | 14 | - | 64001977 | 64003429 | 305 | 17 | 26 |
| XR_297162.1 | 14 | + | 64105001 | 64105896 | 575 | 189 | 132 |
| XR_297163.1 | 14 | + | 64105001 | 64105896 | 579 | 189 | 133 |
| XR_308539.1 | 14 | - | 64223748 | 64235115 | 607 | 12 | 16 |
| XR_308540.1 | 18 | - | 64223748 | 64235115 | 489 | 12 | 13 |
| XR_308069.1 | 18 | + | 64599079 | 64771634 | 820 | 9 | 10 |
| XR_298778.1 | 18 | + | 64611921 | 64617040 | 528 | 20 | 31 |
| XR_298779.1 | 8 | + | 64611921 | 64617040 | 549 | 20 | 35 |
| XM_003360380.3 | 8 | - | 64760340 | 64776631 | 531 | 4 | 8 |
| XR_307570.1 | 1 | - | 65152326 | 65152745 | 310 | 8 | 10 |
| XM_005668223.1 | 5 | - | 65415352 | 65418616 | 1677 | 36 | 39 |
| XR_298478.1 | 8 | - | 65457800 | 65472176 | 3113 | 605 | 171 |
| XR_306029.1 | 8 | - | 65588649 | 65604460 | 854 | 56 | 48 |
| XR_297487.1 | 9 | - | 66297887 | 66330790 | 422 | 1 | 2 |
| XR_135135.2 | 9 | - | 66314440 | 66329011 | 696 | 543 | 373 |
| XR_299147.1 | 9 | - | 66433251 | 66437168 | 1183 | 37 | 69 |
| XR_299148.1 | 13 | - | 66433251 | 66437168 | 949 | 33 | 63 |
| XR_299146.1 | 1 | + | 66434735 | 66439205 | 1507 | 36 | 49 |
| XR_299149.1 | 13 | - | 66484139 | 66484962 | 619 | 115 | 101 |
| XR_299150.1 | 2 | - | 66484139 | 66484962 | 724 | 167 | 128 |
| XR_299151.1 | 2 | + | 66537706 | 66577560 | 533 | 0 | 1 |
| XM_005664072.1 | 2 | - | 66760204 | 66839658 | 7774 | 10 | 80 |
| XM_005664965.1 | 2 | - | 66815282 | 66928245 | 2676 | 1105 | 709 |
| XR_299153.1 | 2 | - | 66843379 | 66912034 | 953 | 1 | 7 |
| XR_299154.1 | 5 | - | 66843379 | 66912034 | 824 | 1 | 6 |
| XR_299155.1 | 6 | - | 66843379 | 66912034 | 900 | 1 | 5 |
| XR_300785.1 | 6 | - | 66896988 | 66971133 | 444 | 0 | 5 |
| XR_299160.1 | 6 | + | 66945472 | 66977029 | 1325 | 7 | 18 |
| XR_299161.1 | 6 | + | 66945472 | 66977029 | 1304 | 7 | 18 |
| XR_299165.1 | 6 | - | 66990651 | 66996762 | 1439 | 1 | 0 |
| XR_306311.1 | 6 | + | 67077598 | 67133247 | 510 | 2 | 14 |
| XR_307585.1 | 6 | + | 67079108 | 67114782 | 1950 | 2265 | 1393 |
| XR_307586.1 | 5 | + | 67079108 | 67114782 | 1855 | 2208 | 1372 |
| XR_307588.1 | 5 | + | 67253414 | 67561771 | 247 | 16 | 2 |
| XR_305506.1 | 1 | + | 67430454 | 67432551 | 704 | 4 | 8 |
| XR_303531.1 | 1 | + | 67716137 | 67721254 | 992 | 34 | 44 |
| XR_303532.1 | 1 | + | 67716137 | 67721254 | 1047 | 35 | 44 |
| XR_303533.1 | 1 | + | 67716137 | 67721254 | 905 | 33 | 44 |
| XR_303540.1 | 1 | + | 67917477 | 67992641 | 533 | 0 | 6 |
| XR_304522.1 | 1 | - | 68081512 | 68106575 | 970 | 99 | 87 |
| XR_304523.1 | 1 | - | 68237800 | 68281460 | 717 | 481 | 350 |
| XR_135162.2 | 1 | + | 68638310 | 68651650 | 1249 | 27 | 14 |
| XR_302361.1 | 3 | + | 68638310 | 68651650 | 1327 | 29 | 20 |
| XR_115757.4 | 3 | + | 68654429 | 68657910 | 2679 | 4454 | 3760 |
| XR_298095.1 | 2 | - | 68885257 | 68890957 | 658 | 7 | 11 |
| XR_306051.1 | 15 | + | 69304646 | 69319921 | 882 | 36 | 59 |
| XR_300104.1 | 6 | + | 69520092 | 69651090 | 411 | 2 | 2 |
| XR_309248.1 | 7 | + | 70658762 | 70667000 | 1539 | 4 | 10 |
| XR_304526.1 | 13 | + | 70661874 | 71013124 | 428 | 4 | 3 |
| XR_304928.1 | 1 | - | 70885315 | 70891375 | 1123 | 21 | 20 |
| XR_306326.1 | 1 | - | 70913094 | 70915346 | 1729 | 3 | 5 |
| XR_306327.1 | 1 | - | 70913094 | 70915346 | 1622 | 3 | 4 |
| XR_304527.1 | 1 | + | 71068340 | 71237578 | 273 | 1 | 6 |
| XR_308096.1 | 1 | - | 71257371 | 71269664 | 581 | 4 | 10 |
| XR_306340.1 | 1 | - | 71532459 | 71699936 | 2333 | 4 | 32 |
| XR_299876.1 | 4 | - | 71614884 | 71620576 | 837 | 1 | 5 |
| XR_299877.1 | 9 | - | 71614884 | 71620576 | 758 | 0 | 1 |
| XM_005664140.1 | 9 | - | 71675516 | 71753922 | 819 | 163 | 127 |
| XR_303945.1 | 9 | + | 71812970 | 71825239 | 922 | 1 | 8 |
| XR_303946.1 | 10 | + | 71812970 | 71825239 | 945 | 1 | 8 |
| XR_303947.1 | 1 | + | 71812970 | 71825239 | 1183 | 10 | 10 |
| XR_303948.1 | 1 | + | 71812970 | 71825239 | 1136 | 2 | 8 |
| XR_303949.1 | 15 | + | 71812970 | 71825239 | 874 | 1 | 8 |
| XR_303950.1 | 1 | + | 71812970 | 71825239 | 1734 | 7 | 11 |
| XR_305520.1 | 1 | + | 71853945 | 71866210 | 637 | 1 | 4 |
| XR_305521.1 | 1 | + | 71853945 | 71866210 | 571 | 1 | 4 |
| XR_297711.1 | 14 | + | 71908633 | 71917720 | 987 | 327 | 211 |
| XR_306926.1 | 1 | - | 71933441 | 71958425 | 426 | 0 | 6 |
| XR_306929.1 | 4 | + | 71982610 | 71988551 | 4729 | 426 | 489 |
| XR_306930.1 | 14 | + | 71982610 | 71988551 | 4833 | 437 | 499 |
| XR_305522.1 | 14 | + | 72079729 | 72161280 | 276 | 222 | 164 |
| XR_305523.1 | 16 | + | 72079729 | 72161280 | 449 | 221 | 159 |
| XR_305524.1 | 3 | + | 72079729 | 72161280 | 352 | 196 | 136 |
| XR_305525.1 | 2 | + | 72079729 | 72161280 | 354 | 210 | 147 |
| XR_299943.1 | 2 | + | 72243099 | 72243875 | 203 | 17 | 8 |
| XR_302389.1 | 9 | - | 72756120 | 72793810 | 439 | 44 | 35 |
| XR_305528.1 | 14 | - | 72766822 | 72919764 | 383 | 1 | 3 |
| XR_300445.1 | 14 | + | 72779920 | 72804940 | 261 | 2 | 1 |
| XR_303556.1 | 3 | - | 72903835 | 72955021 | 739 | 183 | 95 |
| XM_003121326.4 | 3 | + | 73119758 | 73124368 | 2203 | 304 | 1382 |
| XR_299448.1 | 3 | + | 73168000 | 73175039 | 645 | 7 | 13 |
| XR_299449.1 | 13 | + | 73168000 | 73175039 | 533 | 7 | 12 |
| XR_306344.1 | 6 | + | 73319731 | 73327581 | 845 | 2 | 7 |
| XR_300799.1 | 6 | + | 73510556 | 73539717 | 1020 | 39 | 49 |
| XR_300800.1 | 4 | + | 73510556 | 73539717 | 1106 | 51 | 58 |
| XR_309497.1 | 6 | + | 73700096 | 73707034 | 340 | 3 | 6 |
| XR_308569.1 | 1 | + | 73757062 | 73975096 | 364 | 13 | 9 |
| XR_308570.1 | 1 | + | 73757062 | 73975096 | 397 | 13 | 9 |
| XR_305532.1 | 14 | - | 73766509 | 73777601 | 528 | 10 | 25 |
| XR_305534.1 | 14 | + | 73792726 | 73982962 | 325 | 107 | 110 |
| XR_305535.1 | 3 | + | 73792726 | 73982962 | 383 | 112 | 110 |
| XR_305536.1 | 1 | + | 73792726 | 73982962 | 727 | 144 | 160 |
| XR_300804.1 | 11 | - | 73805547 | 73833458 | 256 | 2 | 2 |
| XR_299800.1 | 13 | + | 74063358 | 74067641 | 1188 | 2 | 9 |
| XR_299801.1 | 13 | + | 74063358 | 74067641 | 1079 | 2 | 9 |
| XR_299802.1 | 14 | + | 74063358 | 74067641 | 1182 | 2 | 9 |
| XR_306937.1 | 16 | + | 74180120 | 74452310 | 629 | 89 | 67 |
| XR_308572.1 | 6 | - | 74283392 | 74287961 | 764 | 8 | 8 |
| XR_302995.1 | 4 | - | 74388622 | 74393134 | 824 | 3 | 4 |
| XR_302996.1 | 12 | - | 74388622 | 74393134 | 756 | 3 | 3 |
| XR_308104.1 | 9 | + | 74396040 | 74445215 | 1087 | 8 | 13 |
| XR_308105.1 | 5 | + | 74396040 | 74445215 | 828 | 6 | 14 |
| XR_298335.1 | 5 | + | 74526462 | 74599657 | 3062 | 58 | 74 |
| XR_298336.1 | 5 | + | 74526462 | 74599657 | 2992 | 58 | 73 |
| XR_304530.1 | 5 | - | 74537765 | 74542945 | 417 | 7 | 2 |
| XR_303007.1 | 5 | - | 75294209 | 75296319 | 737 | 1167 | 596 |
| XR_306938.1 | 5 | + | 75449018 | 75450819 | 882 | 29 | 63 |
| XR_303959.1 | 7 | - | 75451207 | 75458957 | 2179 | 2042 | 1711 |
| XR_300811.1 | 7 | + | 75458244 | 75490563 | 1033 | 28 | 34 |
| XR_300812.1 | 8 | + | 75458244 | 75490563 | 1093 | 33 | 37 |
| XR_306941.1 | 8 | + | 75843222 | 75845049 | 653 | 2 | 3 |
| XR_298066.1 | 8 | + | 76158950 | 76163582 | 1145 | 65 | 73 |
| XR_302414.1 | 8 | - | 76306959 | 76320767 | 807 | 0 | 11 |
| XR_302415.1 | 8 | - | 76306959 | 76320767 | 765 | 0 | 11 |
| XR_302416.1 | 8 | - | 76306959 | 76320767 | 725 | 0 | 11 |
| XR_305546.1 | 8 | + | 76349285 | 76587343 | 760 | 4 | 9 |
| XR_305547.1 | 9 | + | 76349285 | 76587343 | 706 | 3 | 8 |
| XR_130914.3 | 9 | - | 76514352 | 76516982 | 500 | 4 | 1 |
| XR_298337.1 | 9 | - | 76517870 | 76547407 | 812 | 2 | 6 |
| XR_302427.1 | 9 | - | 77167358 | 77192315 | 1350 | 10 | 29 |
| XR_302428.1 | 9 | - | 77167358 | 77192315 | 1047 | 3 | 17 |
| XR_303569.1 | 9 | - | 77186542 | 77241769 | 461 | 208 | 130 |
| XR_309571.1 | 9 | + | 77360550 | 77509258 | 796 | 2 | 3 |
| XR_309572.1 | 10 | + | 77360550 | 77509258 | 687 | 2 | 3 |
| XR_297614.1 | 12 | + | 77378209 | 77387751 | 637 | 3 | 11 |
| XR_297615.1 | 13 | + | 77378209 | 77387751 | 770 | 21 | 33 |
| XR_297613.1 | 15 | - | 77421716 | 77427099 | 2475 | 10 | 31 |
| XR_301825.1 | 5 | - | 77465079 | 77472232 | 519 | 8 | 12 |
| XR_301831.1 | 9 | + | 77563317 | 77567518 | 640 | 77 | 69 |
| XR_301832.1 | 9 | + | 77563317 | 77567518 | 809 | 118 | 79 |
| XR_301833.1 | 9 | + | 77563317 | 77567518 | 505 | 53 | 53 |
| XR_301834.1 | 4 | - | 77569613 | 77579457 | 669 | 8 | 9 |
| NR_045015.1 | 6 | + | 77636229 | 77769907 | 1346 | 915 | 405 |
| NR_045016.1 | 7 | + | 77636229 | 77769907 | 1280 | 369 | 221 |
| XR_303558.1 | 7 | + | 77636229 | 77769907 | 2592 | 4263 | 1883 |
| XR_303559.1 | 7 | + | 77636229 | 77769907 | 1800 | 3540 | 1529 |
| XR_303560.1 | 7 | + | 77636229 | 77769907 | 2175 | 3772 | 1648 |
| XR_304957.1 | 7 | - | 77684720 | 77690559 | 427 | 8 | 2 |
| XR_300825.1 | 7 | + | 77694902 | 77696055 | 413 | 15 | 10 |
| XR_304958.1 | 7 | + | 77897628 | 77906183 | 697 | 7 | 8 |
| XR_304959.1 | 11 | + | 77897628 | 77906183 | 726 | 7 | 8 |
| XR_304960.1 | 13 | + | 77897628 | 77906183 | 1155 | 9 | 18 |
| XR_304961.1 | 2 | + | 77897628 | 77906183 | 611 | 7 | 8 |
| XR_304962.1 | 7 | + | 77897628 | 77906183 | 600 | 4 | 7 |
| XR_304963.1 | 7 | + | 77897628 | 77906183 | 514 | 4 | 7 |
| XR_298343.1 | 7 | + | 78167779 | 78227777 | 391 | 2 | 4 |
| XR_301839.1 | X | - | 78237427 | 78285104 | 415 | 21 | 13 |
| XR_301840.1 | 11 | - | 78237427 | 78285104 | 456 | 18 | 12 |
| XR_303967.1 | 11 | + | 78429784 | 78437012 | 215 | 1 | 0 |
| XR_300829.1 | 5 | + | 78538921 | 78606947 | 585 | 3 | 2 |
| XR_305581.1 | 5 | - | 78553447 | 78554400 | 463 | 1 | 4 |
| XR_305582.1 | 12 | - | 78553447 | 78554400 | 460 | 1 | 4 |
| XR_116057.2 | 16 | + | 78553680 | 78576057 | 2497 | 944 | 745 |
| XR_298793.1 | 17 | + | 78553680 | 78576057 | 2611 | 1002 | 782 |
| XR_298794.1 | 3 | + | 78553680 | 78576057 | 2079 | 60 | 53 |
| XR_298111.1 | 12 | + | 78612083 | 78617004 | 378 | 343 | 327 |
| XR_300227.1 | 9 | + | 78696851 | 78712900 | 640 | 4 | 7 |
| XR_297618.1 | 9 | - | 79046065 | 79052053 | 1116 | 0 | 3 |
| XR_297619.1 | 9 | - | 79046065 | 79052053 | 1113 | 0 | 3 |
| XR_297620.1 | 13 | - | 79046065 | 79052053 | 1071 | 0 | 3 |
| XR_297621.1 | 2 | - | 79046065 | 79052053 | 955 | 0 | 2 |
| XR_298112.1 | 2 | - | 79201330 | 79210219 | 1910 | 162 | 115 |
| XR_303026.1 | 6 | - | 79322942 | 79330336 | 1063 | 8 | 11 |
| XR_303027.1 | 11 | - | 79322942 | 79330336 | 1101 | 8 | 11 |
| XR_303028.1 | 3 | - | 79322942 | 79330336 | 1392 | 9 | 13 |
| XR_303029.1 | 8 | - | 79322942 | 79330336 | 1107 | 9 | 11 |
| XR_303030.1 | 12 | - | 79322942 | 79330336 | 1059 | 9 | 11 |
| XR_303031.1 | 5 | - | 79322942 | 79330336 | 973 | 8 | 10 |
| XR_303032.1 | 6 | - | 79322942 | 79330336 | 1162 | 6 | 11 |
| XR_303034.1 | 1 | + | 79342919 | 79345820 | 985 | 13 | 23 |
| XR_303047.1 | 1 | + | 79655269 | 79685488 | 791 | 23 | 28 |
| XR_302445.1 | 1 | - | 80329969 | 80331234 | 330 | 110 | 78 |
| XR_298602.1 | 4 | - | 80348900 | 80419781 | 1091 | 10 | 17 |
| XR_298603.1 | 5 | - | 80348900 | 80419781 | 977 | 10 | 15 |
| XR_298604.1 | 1 | - | 80348900 | 80419781 | 1041 | 10 | 16 |
| XR_307625.1 | 7 | + | 80554449 | 81080365 | 311 | 32 | 39 |
| XR_302449.1 | 8 | - | 81072355 | 81097857 | 2884 | 8 | 24 |
| XR_302450.1 | 13 | - | 81072355 | 81097857 | 2609 | 7 | 21 |
| XR_302451.1 | 13 | - | 81072355 | 81097857 | 2775 | 8 | 24 |
| XR_302452.1 | 2 | - | 81072355 | 81097857 | 2500 | 7 | 21 |
| XR_302453.1 | 2 | - | 81072355 | 81097857 | 2278 | 6 | 13 |
| XR_300470.1 | 6 | - | 81457667 | 81481427 | 653 | 3 | 10 |
| XR_301844.1 | 6 | + | 81617424 | 81640144 | 1055 | 295 | 388 |
| XR_301845.1 | 6 | + | 81617424 | 81640144 | 923 | 268 | 349 |
| XR_301846.1 | 7 | + | 81617424 | 81640144 | 925 | 269 | 349 |
| XR_301847.1 | 14 | + | 81617424 | 81640144 | 840 | 196 | 248 |
| XR_300432.1 | 6 | - | 81796135 | 81798759 | 484 | 3 | 8 |
| XR_308116.1 | 6 | + | 81804907 | 81806589 | 882 | 19 | 46 |
| XR_302470.1 | 6 | + | 81911252 | 81944077 | 491 | 3 | 2 |
| XR_302471.1 | 9 | + | 81911252 | 81944077 | 527 | 4 | 2 |
| XR_305592.1 | 9 | - | 82079543 | 82106106 | 596 | 1 | 2 |
| XR_303058.1 | 18 | - | 82087618 | 82111961 | 584 | 1 | 11 |
| XR_300842.1 | 18 | + | 82193289 | 82217780 | 418 | 2 | 2 |
| XR_305594.1 | 18 | + | 82268640 | 82361822 | 340 | 1 | 3 |
| XR_307630.1 | 18 | - | 82289740 | 82305602 | 1793 | 60 | 71 |
| XR_304980.1 | 18 | - | 82306876 | 82322192 | 587 | 33 | 33 |
| XR_304981.1 | 18 | - | 82306876 | 82322192 | 651 | 40 | 36 |
| XR_308123.1 | 18 | - | 82436751 | 82446225 | 1142 | 27 | 19 |
| XR_306974.1 | 18 | - | 82442107 | 82548440 | 306 | 33 | 20 |
| XR_302475.1 | 18 | - | 82563268 | 83162816 | 1347 | 7 | 22 |
| XR_303059.1 | 18 | + | 82794800 | 82834274 | 2715 | 512 | 317 |
| XR_297488.1 | 18 | - | 82864967 | 82950642 | 813 | 152 | 99 |
| XR_297489.1 | 18 | - | 82864967 | 82950642 | 385 | 45 | 28 |
| XR_303603.1 | 2 | + | 82913191 | 82917675 | 330 | 5 | 6 |
| XR_299664.1 | 2 | + | 83223432 | 83224720 | 440 | 80 | 122 |
| XR_299665.1 | 2 | + | 83223432 | 83224720 | 371 | 64 | 93 |
| XR_301851.1 | 2 | + | 83278418 | 83427331 | 453 | 1 | 2 |
| XR_301853.1 | 14 | - | 83369511 | 83380090 | 509 | 40 | 64 |
| XR_309401.1 | 14 | - | 83446310 | 83547516 | 525 | 3 | 13 |
| XR_298808.1 | 14 | - | 83550040 | 83570438 | 1202 | 5 | 9 |
| XR_299670.1 | 14 | + | 84320380 | 84338947 | 643 | 190 | 154 |
| XR_299671.1 | 14 | + | 84320380 | 84338947 | 544 | 156 | 124 |
| XR_298815.1 | 14 | - | 84380071 | 84416482 | 551 | 11 | 17 |
| XR_299851.1 | 14 | - | 84535416 | 84546882 | 664 | 80 | 66 |
| XR_301855.1 | 14 | + | 84731094 | 84733771 | 1239 | 8 | 9 |
| XR_309259.1 | 1 | + | 84903297 | 84922577 | 473 | 18 | 11 |
| XR_309260.1 | 10 | + | 84903297 | 84922577 | 759 | 19 | 11 |
| XR_309261.1 | 4 | + | 84903297 | 84922577 | 713 | 16 | 11 |
| XR_309262.1 | 1 | + | 84903297 | 84922577 | 537 | 18 | 11 |
| XR_309263.1 | 3 | + | 84903297 | 84922577 | 695 | 19 | 11 |
| XR_309264.1 | 7 | + | 84903297 | 84922577 | 491 | 15 | 11 |
| XR_309265.1 | 13 | + | 84903297 | 84922577 | 716 | 2 | 1 |
| XR_303982.1 | 13 | + | 85533523 | 85536155 | 1909 | 52602 | 57504 |
| XR_303983.1 | 3 | + | 85533523 | 85536155 | 1837 | 52476 | 57363 |
| XR_303984.1 | X | - | 85593745 | 85597231 | 1056 | 3088 | 3295 |
| XR_303985.1 | 6 | - | 85593745 | 85597231 | 1375 | 3564 | 3824 |
| XR_303986.1 | 6 | - | 85593745 | 85597231 | 1357 | 3541 | 3809 |
| XR_303987.1 | 6 | - | 85593745 | 85597231 | 1167 | 2642 | 2814 |
| XR_302482.1 | 6 | - | 85742635 | 85803328 | 610 | 84 | 53 |
| XR_299782.1 | 6 | + | 85771616 | 85789559 | 1116 | 6 | 2 |
| XR_299783.1 | 6 | + | 85771616 | 85789559 | 1003 | 6 | 2 |
| XR_299784.1 | 6 | + | 85771616 | 85789559 | 1114 | 6 | 2 |
| XR_304991.1 | 6 | - | 85915063 | 85917156 | 275 | 0 | 4 |
| XR_307635.1 | 15 | + | 85916338 | 86247444 | 621 | 1 | 6 |
| XR_303619.1 | 17 | - | 86094295 | 86240246 | 813 | 94 | 82 |
| NR_033705.1 | 13 | - | 86109966 | 86124428 | 931 | 3909 | 3210 |
| XR_304583.1 | 13 | - | 86109966 | 86124428 | 1256 | 4381 | 3595 |
| XR_304584.1 | 13 | - | 86109966 | 86124428 | 1047 | 4143 | 3413 |
| XR_309595.1 | 11 | + | 86259016 | 86260562 | 419 | 1 | 6 |
| XR_305609.1 | 11 | - | 86527750 | 86544757 | 1504 | 69 | 38 |
| XR_305006.1 | 14 | - | 86617601 | 86623835 | 1503 | 13 | 16 |
| XR_305007.1 | 14 | - | 86617601 | 86623835 | 1578 | 15 | 16 |
| XR_306986.1 | 6 | - | 87022119 | 87027679 | 223 | 3 | 3 |
| XR_303621.1 | 8 | + | 87122584 | 87175011 | 1172 | 3 | 8 |
| XR_298348.1 | 3 | + | 87194306 | 87246779 | 739 | 84 | 91 |
| XR_298349.1 | 3 | + | 87194306 | 87246779 | 848 | 143 | 145 |
| XR_298350.1 | 3 | + | 87194306 | 87246779 | 806 | 98 | 95 |
| XR_298932.1 | 3 | - | 87459598 | 87465271 | 735 | 10 | 20 |
| XR_298933.1 | 3 | - | 87570013 | 87609083 | 1345 | 357 | 279 |
| XR_303622.1 | 13 | + | 87639593 | 87652560 | 331 | 0 | 3 |
| XR_297317.1 | 13 | + | 87664864 | 88052718 | 575 | 3 | 6 |
| XR_307642.1 | 13 | + | 87940863 | 87953938 | 415 | 1 | 0 |
| XR_299510.1 | X | + | 87966309 | 87993761 | 1109 | 3 | 10 |
| XR_299511.1 | X | + | 87966309 | 87993761 | 1010 | 2 | 10 |
| XR_303623.1 | X | - | 88417116 | 88425466 | 1775 | 18 | 24 |
| XR_305614.1 | X | + | 88735126 | 88765983 | 1128 | 86 | 105 |
| XR_304604.1 | 12 | - | 89035239 | 89049426 | 577 | 5 | 12 |
| XR_304613.1 | 9 | - | 89208965 | 89236647 | 2258 | 9 | 17 |
| XR_304614.1 | 9 | - | 89208965 | 89236647 | 2254 | 9 | 17 |
| XR_303635.1 | 9 | + | 89227083 | 89247231 | 591 | 23 | 26 |
| XR_303634.1 | 12 | - | 89479424 | 89490051 | 630 | 69 | 96 |
| XR_297491.1 | 2 | + | 89522556 | 89705132 | 374 | 2 | 5 |
| XR_297721.1 | 2 | + | 89909370 | 89944647 | 744 | 7 | 6 |
| XR_304618.1 | 11 | - | 89957988 | 90002528 | 308 | 0 | 1 |
| XR_297722.1 | 1 | + | 89969661 | 89988621 | 800 | 2 | 5 |
| XR_297723.1 | 3 | + | 89969661 | 89988621 | 797 | 2 | 5 |
| XR_303640.1 | 15 | + | 90055019 | 90088357 | 861 | 5 | 11 |
| XR_297727.1 | 18 | - | 90243805 | 90251319 | 711 | 0 | 3 |
| XR_298817.1 | 2 | - | 90287490 | 90346202 | 981 | 8 | 15 |
| XR_298818.1 | 7 | - | 90287490 | 90346202 | 834 | 8 | 12 |
| XR_135257.2 | 7 | - | 90433903 | 90439551 | 1304 | 14 | 11 |
| XR_298625.1 | 1 | - | 90433903 | 90439551 | 1632 | 17 | 12 |
| XR_298626.1 | 15 | - | 90433903 | 90439551 | 1570 | 11 | 12 |
| XR_298627.1 | X | - | 90433903 | 90439551 | 1603 | 14 | 12 |
| XR_298628.1 | 4 | - | 90433903 | 90439551 | 1368 | 14 | 11 |
| XR_298629.1 | 4 | - | 90433903 | 90439551 | 1618 | 16 | 11 |
| XR_298630.1 | 7 | - | 90433903 | 90439551 | 1504 | 15 | 9 |
| XR_298631.1 | 8 | - | 90433903 | 90439551 | 1445 | 15 | 9 |
| XR_305017.1 | 9 | - | 90780338 | 90789711 | 732 | 6 | 8 |
| XR_303642.1 | 4 | + | 90919800 | 91114826 | 1401 | 8 | 39 |
| XR_298825.1 | 6 | + | 91132970 | 91141045 | 710 | 21 | 33 |
| XR_303086.1 | 3 | - | 91285119 | 91288620 | 886 | 3 | 9 |
| XR_303646.1 | 12 | + | 91422115 | 91561899 | 950 | 2 | 7 |
| XR_303647.1 | 9 | + | 91422115 | 91561899 | 906 | 3 | 7 |
| XR_303648.1 | 9 | + | 91422115 | 91561899 | 943 | 3 | 8 |
| XR_303649.1 | 13 | + | 91422115 | 91561899 | 865 | 2 | 9 |
| XR_299035.1 | 13 | + | 91597881 | 91600765 | 276 | 31 | 42 |
| XR_299036.1 | 2 | + | 91597881 | 91600765 | 428 | 33 | 34 |
| XR_299037.1 | 15 | + | 91609323 | 91644866 | 320 | 3 | 8 |
| XR_301877.1 | 15 | + | 91902465 | 92122360 | 1034 | 153 | 256 |
| XR_301878.1 | 3 | + | 91902465 | 92122360 | 1031 | 153 | 256 |
| XR_301879.1 | 3 | + | 91902465 | 92122360 | 974 | 153 | 256 |
| XR_299038.1 | 15 | - | 92011394 | 92019461 | 632 | 6 | 15 |
| XM_005656351.1 | 17 | - | 92516504 | 92518846 | 1315 | 295 | 300 |
| XR_297427.1 | 18 | + | 92547745 | 92755362 | 541 | 14 | 8 |
| XR_299042.1 | 18 | - | 92875662 | 92911135 | 710 | 17 | 35 |
| XR_299043.1 | 8 | - | 92875662 | 92911135 | 850 | 17 | 43 |
| XR_299044.1 | 10 | - | 92875662 | 92911135 | 881 | 17 | 41 |
| XR_299045.1 | 1 | - | 92875662 | 92911135 | 912 | 16 | 41 |
| XR_299049.1 | 1 | - | 93047287 | 93051702 | 1750 | 822 | 830 |
| XR_306991.1 | 5 | + | 93051936 | 93053724 | 819 | 55 | 91 |
| XR_306992.1 | 8 | + | 93051936 | 93053724 | 627 | 49 | 75 |
| XR_308147.1 | 10 | - | 93326913 | 93341435 | 277 | 5 | 10 |
| XR_305026.1 | 6 | + | 93383945 | 93439528 | 459 | 11 | 6 |
| XR_303098.1 | 5 | - | 93622747 | 93625502 | 643 | 109 | 107 |
| XR_302514.1 | 2 | + | 93670769 | 93681093 | 326 | 3 | 15 |
| XR_304016.1 | 9 | + | 94086235 | 94101569 | 540 | 0 | 4 |
| XR_303110.1 | 9 | - | 94252706 | 94263977 | 679 | 22 | 16 |
| XR_303111.1 | 9 | - | 94252706 | 94263977 | 676 | 22 | 16 |
| XR_303112.1 | 9 | - | 94252706 | 94263977 | 1614 | 62 | 47 |
| XR_300520.1 | 9 | - | 94525300 | 94541235 | 525 | 3 | 3 |
| XR_300521.1 | 6 | - | 94525300 | 94541235 | 528 | 3 | 3 |
| XR_303116.1 | 5 | + | 94673976 | 94683184 | 261 | 0 | 4 |
| XR_308152.1 | 5 | - | 95042671 | 95049500 | 585 | 33 | 54 |
| XR_304023.1 | 5 | - | 95120929 | 95125794 | 3225 | 28199 | 14715 |
| XR_298943.1 | 5 | - | 95199490 | 95219084 | 204 | 15 | 14 |
| XR_298488.1 | 5 | + | 95211025 | 95369880 | 431 | 2 | 4 |
| XR_304025.1 | 13 | + | 95599243 | 95600713 | 373 | 2 | 6 |
| XR_298634.1 | 13 | + | 95719122 | 95724388 | 379 | 3 | 3 |
| XR_298635.1 | 15 | + | 95719122 | 95724388 | 338 | 1 | 3 |
| XR_298636.1 | 16 | + | 95719122 | 95724388 | 376 | 3 | 3 |
| XR_297089.1 | 11 | - | 96209046 | 96313749 | 763 | 241 | 332 |
| XR_297090.1 | 11 | - | 96209046 | 96313749 | 842 | 243 | 342 |
| XR_309380.1 | 11 | - | 96281234 | 96616438 | 630 | 7 | 12 |
| XR_309381.1 | 11 | - | 96281234 | 96616438 | 555 | 7 | 12 |
| XR_307677.1 | 11 | - | 96322654 | 96425391 | 778 | 136 | 75 |
| XR_307003.1 | 7 | - | 96343394 | 96349266 | 235 | 21 | 13 |
| XR_307681.1 | 17 | - | 96449517 | 96516562 | 2103 | 57 | 72 |
| XR_309335.1 | 17 | + | 96567001 | 96608058 | 333 | 0 | 5 |
| XR_305033.1 | 17 | - | 96641208 | 96693568 | 778 | 7 | 10 |
| XR_307007.1 | 17 | + | 96931579 | 96951501 | 553 | 3 | 4 |
| XM_005669950.1 | 17 | + | 96985590 | 96985967 | 804 | 225 | 152 |
| XR_305037.1 | 5 | + | 97013360 | 97056003 | 656 | 238 | 221 |
| XR_305038.1 | 15 | + | 97013360 | 97056003 | 576 | 122 | 119 |
| XR_305050.1 | 14 | + | 97441898 | 97450209 | 293 | 1 | 0 |
| XR_297274.1 | 14 | + | 97593212 | 97633163 | 387 | 32 | 34 |
| XR_302529.1 | 3 | + | 98489919 | 99130510 | 631 | 2 | 9 |
| XR_309270.1 | 12 | - | 98758563 | 98821344 | 780 | 117 | 98 |
| XR_309271.1 | 14 | - | 98758563 | 98821344 | 694 | 117 | 98 |
| XR_304052.1 | 14 | + | 99018171 | 99065181 | 3107 | 293 | 311 |
| XM_003480325.2 | 14 | - | 99036269 | 99043229 | 1197 | 4311 | 2900 |
| XM_003480326.2 | 14 | - | 99036269 | 99043229 | 1067 | 4239 | 2852 |
| XM_005659458.1 | 18 | - | 99036269 | 99043229 | 1071 | 4240 | 2852 |
| XM_005659459.1 | X | - | 99036269 | 99043229 | 1201 | 4311 | 2901 |
| XR_300883.1 | 3 | + | 99311334 | 99314082 | 410 | 371 | 337 |
| XR_301888.1 | 3 | + | 99339739 | 99922668 | 286 | 1 | 1 |
| XR_307690.1 | 1 | + | 99888802 | 99907659 | 859 | 3 | 12 |
| XR_307691.1 | 1 | + | 99888802 | 99907659 | 854 | 3 | 12 |
| XR_307692.1 | 12 | + | 99888802 | 99907659 | 827 | 2 | 12 |
| XR_307693.1 | 7 | + | 99888802 | 99907659 | 770 | 0 | 9 |
| XM_005672042.1 | 16 | - | 100065679 | 100066780 | 936 | 170 | 174 |
| XR_304631.1 | 5 | + | 100075826 | 100077936 | 1017 | 81 | 50 |
| XR_304632.1 | 2 | - | 100132090 | 100139431 | 1491 | 160 | 467 |
| XR_297333.1 | 2 | + | 100321612 | 100324272 | 484 | 26 | 8 |
| XR_303666.1 | 2 | + | 100429452 | 100461030 | 1981 | 17 | 27 |
| XR_303667.1 | 7 | + | 100429452 | 100461030 | 1870 | 16 | 20 |
| XR_297338.1 | 9 | + | 100435552 | 100452440 | 977 | 871 | 695 |
| XR_305624.1 | 18 | - | 100816875 | 100848365 | 1561 | 238 | 235 |
| XR_298642.1 | 7 | + | 100846687 | 100854138 | 869 | 7 | 8 |
| XR_299761.1 | 7 | + | 100853837 | 101152401 | 471 | 3 | 3 |
| XR_303152.1 | 2 | - | 101083811 | 101090824 | 282 | 15 | 36 |
| XR_307699.1 | 2 | + | 101094689 | 101137452 | 3446 | 444 | 378 |
| XR_307700.1 | 2 | + | 101094689 | 101137452 | 3194 | 426 | 365 |
| XR_301234.1 | 17 | + | 101145447 | 101211391 | 1117 | 3 | 13 |
| XR_304057.1 | 9 | + | 101179649 | 101209794 | 1345 | 7 | 9 |
| XR_300181.1 | 14 | + | 101702555 | 101753154 | 581 | 47 | 25 |
| XR_300182.1 | 18 | + | 101702555 | 101753154 | 603 | 45 | 26 |
| XR_300183.1 | 18 | + | 101702555 | 101753154 | 718 | 52 | 29 |
| XR_300184.1 | 18 | + | 101702555 | 101753154 | 726 | 59 | 37 |
| XR_300185.1 | 18 | + | 101702555 | 101753154 | 615 | 49 | 26 |
| XR_302530.1 | 18 | + | 101800461 | 101818001 | 1882 | 18 | 35 |
| XR_297173.1 | 18 | + | 101815969 | 101851201 | 673 | 18 | 28 |
| XR_307710.1 | 18 | - | 101924146 | 101936888 | 1140 | 0 | 12 |
| XR_307711.1 | 5 | - | 101924146 | 101936888 | 1037 | 0 | 10 |
| XR_307712.1 | 7 | - | 101924146 | 101936888 | 1231 | 0 | 12 |
| XR_307713.1 | 2 | - | 101924146 | 101936888 | 1115 | 0 | 11 |
| XR_307714.1 | 1 | - | 101924146 | 101936888 | 1023 | 0 | 11 |
| XR_307715.1 | 14 | - | 101924146 | 101936888 | 930 | 0 | 11 |
| XR_302531.1 | 2 | - | 102069048 | 102182738 | 422 | 30 | 45 |
| XR_302532.1 | 9 | - | 102069048 | 102182738 | 597 | 39 | 66 |
| XR_305626.1 | 11 | + | 102434526 | 102555421 | 916 | 189 | 181 |
| XR_305627.1 | 6 | + | 102564176 | 102608310 | 716 | 57 | 38 |
| XR_297504.1 | 7 | + | 102731252 | 102736210 | 640 | 8 | 7 |
| XR_308170.1 | 8 | - | 102904074 | 102951000 | 301 | 1 | 1 |
| XM_001924349.2 | 9 | + | 103347001 | 103351895 | 694 | 34 | 48 |
| XR_309574.1 | 11 | - | 103995354 | 104044644 | 234 | 3 | 7 |
| XR_308173.1 | 11 | - | 104270608 | 104325236 | 667 | 4 | 4 |
| XR_307015.1 | 14 | + | 104471238 | 104799180 | 323 | 2 | 3 |
| XR_303163.1 | 1 | + | 104589533 | 104614247 | 1146 | 31 | 43 |
| XR_304067.1 | 3 | - | 104629408 | 104644003 | 612 | 1 | 6 |
| XR_304068.1 | 2 | - | 104629408 | 104644003 | 744 | 4 | 6 |
| XR_303164.1 | 2 | + | 104755053 | 104799079 | 761 | 109 | 130 |
| XR_303165.1 | 2 | + | 104755053 | 104799079 | 557 | 66 | 71 |
| XR_307016.1 | 2 | + | 104872177 | 104901870 | 826 | 1 | 6 |
| XR_303166.1 | 2 | - | 105071736 | 105113670 | 991 | 9 | 20 |
| XR_303167.1 | 10 | - | 105071736 | 105113670 | 908 | 9 | 20 |
| XR_303168.1 | 6 | - | 105071736 | 105113670 | 1171 | 13 | 21 |
| XR_303169.1 | 11 | - | 105071736 | 105113670 | 907 | 8 | 19 |
| XR_303170.1 | 1 | - | 105071736 | 105113670 | 822 | 7 | 18 |
| XR_130811.2 | 14 | - | 105197311 | 105198419 | 702 | 4321 | 3982 |
| XR_301912.1 | 14 | + | 105276010 | 105412618 | 803 | 33 | 36 |
| XR_303172.1 | 1 | - | 105386771 | 105486406 | 495 | 1 | 1 |
| XR_304070.1 | 1 | + | 105418334 | 105424564 | 702 | 1 | 7 |
| XR_304071.1 | 6 | + | 105418334 | 105424564 | 829 | 2 | 7 |
| XR_304072.1 | 6 | + | 105418334 | 105424564 | 864 | 1 | 8 |
| XR_308178.1 | 6 | - | 105772330 | 105777770 | 551 | 2 | 9 |
| XR_308179.1 | 1 | - | 105885723 | 105893562 | 674 | 66 | 65 |
| XR_300908.1 | 4 | - | 106511500 | 106750239 | 650 | 5 | 13 |
| XR_305636.1 | 5 | - | 106522573 | 106632452 | 634 | 5 | 5 |
| XR_298356.1 | 5 | + | 106563408 | 106578834 | 1391 | 1626 | 1368 |
| XR_307028.1 | 5 | + | 106910198 | 107014731 | 521 | 11 | 10 |
| XR_307741.1 | X | + | 106993920 | 106995615 | 376 | 32 | 29 |
| XR_299673.1 | X | + | 107067893 | 107110871 | 572 | 14 | 8 |
| XR_298643.1 | X | - | 107179783 | 107242591 | 1060 | 18 | 36 |
| XR_305640.1 | X | + | 107683099 | 107688606 | 1893 | 5698 | 6859 |
| XR_307029.1 | 6 | + | 107707197 | 107732495 | 898 | 7 | 22 |
| XR_307750.1 | 6 | + | 108033526 | 108037242 | 1369 | 186 | 217 |
| XR_302552.1 | 8 | - | 108072558 | 108076595 | 1698 | 10 | 18 |
| XR_303179.1 | 17 | - | 108210147 | 108261813 | 364 | 2 | 4 |
| XR_303180.1 | 14 | - | 108210147 | 108261813 | 539 | 3 | 4 |
| XR_303176.1 | 4 | + | 108378739 | 108393773 | 757 | 73 | 96 |
| XR_303177.1 | 4 | + | 108378739 | 108393773 | 855 | 79 | 88 |
| XR_299054.1 | 5 | - | 108529340 | 108631115 | 632 | 4 | 6 |
| XR_300930.1 | 6 | - | 108941524 | 108952743 | 654 | 3 | 20 |
| XR_303184.1 | 6 | + | 109050279 | 109060278 | 827 | 47 | 52 |
| XR_300932.1 | 6 | - | 109054398 | 109061345 | 1007 | 8 | 10 |
| XR_300933.1 | 13 | - | 109054398 | 109061345 | 1058 | 8 | 10 |
| XR_299456.1 | 3 | + | 109069194 | 109074446 | 3010 | 8679 | 7268 |
| XR_299457.1 | 3 | + | 109069194 | 109074446 | 2805 | 7973 | 6663 |
| XR_299458.1 | 6 | - | 109134610 | 109138493 | 515 | 5 | 9 |
| XR_305067.1 | 2 | - | 109202397 | 109284890 | 773 | 38 | 26 |
| XR_309569.1 | 2 | - | 109244218 | 109252065 | 497 | 0 | 1 |
| XR_309288.1 | 1 | - | 109261358 | 109276269 | 612 | 10 | 3 |
| XR_304077.1 | 7 | + | 109339905 | 109671306 | 1943 | 12 | 22 |
| XR_304078.1 | 10 | + | 109339905 | 109671306 | 1707 | 11 | 19 |
| XR_304079.1 | 5 | + | 109339905 | 109671306 | 1763 | 11 | 20 |
| XR_304080.1 | 6 | + | 109339905 | 109671306 | 1790 | 10 | 22 |
| XR_304081.1 | 7 | + | 109339905 | 109671306 | 1789 | 11 | 17 |
| XR_309289.1 | 12 | + | 109516724 | 109542290 | 1955 | 21 | 45 |
| XR_309290.1 | 4 | + | 109516724 | 109542290 | 1366 | 20 | 28 |
| XR_303192.1 | 3 | - | 110226328 | 110268209 | 1002 | 2 | 15 |
| XR_303190.1 | 4 | - | 110339289 | 110380590 | 1375 | 16 | 25 |
| XR_305069.1 | 4 | + | 110450671 | 111427895 | 436 | 5 | 8 |
| XR_303194.1 | 5 | - | 110679943 | 110695339 | 3123 | 252 | 180 |
| XR_303195.1 | 5 | - | 110679943 | 110695339 | 2825 | 205 | 140 |
| XR_303196.1 | 10 | - | 110679943 | 110695339 | 3384 | 247 | 168 |
| XR_297881.1 | 10 | + | 110834949 | 110842958 | 847 | 2 | 4 |
| XR_298258.1 | 1 | - | 110967442 | 110968685 | 444 | 37 | 175 |
| XR_116000.2 | 17 | - | 111234418 | 111264810 | 1436 | 65 | 75 |
| XR_303204.1 | X | + | 111347576 | 111350486 | 688 | 54 | 61 |
| XR_303205.1 | 4 | + | 111347576 | 111350486 | 1126 | 150 | 143 |
| XM_003481269.2 | 6 | - | 111781661 | 111786377 | 503 | 403 | 390 |
| XR_309291.1 | 6 | - | 112010531 | 112100731 | 728 | 8 | 9 |
| XR_307765.1 | 6 | + | 112287771 | 112335178 | 1167 | 7 | 18 |
| XR_302578.1 | 1 | - | 112330110 | 112332613 | 454 | 28 | 36 |
| XR_302581.1 | 1 | + | 112427214 | 112432032 | 465 | 1 | 5 |
| XR_309642.1 | 1 | + | 112527659 | 112550536 | 552 | 59 | 52 |
| XR_297882.1 | 2 | + | 112710919 | 112747593 | 1051 | 4 | 26 |
| XR_308211.1 | X | - | 112716569 | 112723180 | 2002 | 27 | 47 |
| XR_307766.1 | X | + | 112734941 | 112747452 | 898 | 92 | 87 |
| XR_308223.1 | X | + | 113044105 | 113082816 | 670 | 7 | 7 |
| XR_305076.1 | X | + | 113472115 | 113491171 | 885 | 24 | 22 |
| XR_305079.1 | 7 | - | 113539193 | 113825271 | 2198 | 6 | 46 |
| XR_305080.1 | 2 | - | 113539193 | 113825271 | 2266 | 6 | 46 |
| XR_305081.1 | 2 | - | 113539193 | 113825271 | 2082 | 6 | 45 |
| XR_300955.1 | 2 | + | 114066375 | 114119508 | 676 | 5 | 7 |
| XR_300957.1 | 2 | + | 114266687 | 114297565 | 755 | 366 | 395 |
| XR_300958.1 | 2 | + | 114266687 | 114297565 | 821 | 373 | 400 |
| XR_302604.1 | 5 | - | 114495337 | 114495945 | 509 | 29 | 18 |
| XR_305668.1 | 11 | - | 114912525 | 114916796 | 1169 | 9 | 20 |
| XR_301924.1 | 11 | + | 114930145 | 115124845 | 629 | 1 | 6 |
| XR_304089.1 | 2 | + | 115162309 | 115176970 | 589 | 2 | 7 |
| XR_302606.1 | 2 | + | 115177880 | 115460750 | 346 | 68 | 63 |
| XR_299961.1 | 2 | - | 115188068 | 115196184 | 372 | 1 | 0 |
| XR_302611.1 | 3 | + | 115700284 | 116292922 | 997 | 30 | 33 |
| XR_302612.1 | 2 | + | 115700284 | 116292922 | 943 | 26 | 33 |
| XR_302613.1 | 7 | + | 115700284 | 116292922 | 826 | 22 | 30 |
| XR_305093.1 | 14 | + | 116141745 | 116194320 | 594 | 1 | 1 |
| XR_305094.1 | 16 | + | 116141745 | 116194320 | 577 | 1 | 1 |
| XR_305095.1 | Y | + | 116141745 | 116194320 | 591 | 1 | 1 |
| XR_301934.1 | 8 | + | 116257130 | 116278588 | 453 | 18 | 19 |
| XR_298647.1 | 12 | + | 116325237 | 116341436 | 342 | 3 | 4 |
| XR_298264.1 | 12 | + | 116435953 | 117201890 | 422 | 0 | 6 |
| XR_298870.1 | 12 | + | 116437061 | 116439382 | 1280 | 172 | 166 |
| XR_298871.1 | 10 | + | 116437061 | 116439382 | 1208 | 166 | 156 |
| XR_298872.1 | 11 | + | 116437061 | 116439382 | 1097 | 164 | 153 |
| XR_298874.1 | 12 | + | 116574159 | 116587893 | 995 | 20 | 34 |
| XR_298875.1 | 12 | - | 116592901 | 116594233 | 573 | 7 | 5 |
| XR_298876.1 | 12 | - | 116592901 | 116594233 | 743 | 7 | 6 |
| XR_298877.1 | 12 | - | 116592901 | 116594233 | 569 | 7 | 5 |
| XR_116071.2 | 4 | - | 116784929 | 116787100 | 531 | 1592 | 1198 |
| XR_298878.1 | 4 | - | 116784929 | 116787100 | 528 | 1555 | 1157 |
| XR_298879.1 | 1 | - | 116784929 | 116787100 | 819 | 13844 | 13687 |
| XR_299914.1 | 1 | + | 117078912 | 117087595 | 342 | 4 | 5 |
| XM_005661580.1 | 17 | - | 117235187 | 117501698 | 1750 | 1187 | 770 |
| XR_299065.1 | 17 | + | 117247208 | 117628291 | 1020 | 117 | 107 |
| XR_299066.1 | 17 | + | 117247208 | 117628291 | 828 | 107 | 97 |
| XR_299067.1 | 17 | + | 117247208 | 117628291 | 729 | 103 | 92 |
| XR_302621.1 | 17 | - | 117443780 | 117463783 | 1246 | 52 | 38 |
| XR_302622.1 | 17 | - | 117443780 | 117463783 | 1333 | 54 | 38 |
| XR_302623.1 | 17 | - | 117443780 | 117463783 | 1250 | 52 | 38 |
| XR_304093.1 | 17 | + | 117526165 | 117560187 | 438 | 0 | 5 |
| XR_302624.1 | 18 | - | 117601219 | 117608925 | 653 | 6 | 5 |
| XR_302627.1 | 4 | - | 117854026 | 117857827 | 663 | 42 | 46 |
| XR_302628.1 | 4 | - | 117854026 | 117857827 | 563 | 43 | 47 |
| XR_307042.1 | 5 | - | 118172531 | 118614418 | 517 | 9 | 9 |
| XR_298956.1 | 10 | - | 118198046 | 118203226 | 2266 | 411 | 344 |
| XR_298957.1 | 16 | - | 118198046 | 118203226 | 2301 | 405 | 350 |
| XR_298958.1 | 16 | - | 118198046 | 118203226 | 2337 | 404 | 333 |
| XR_298883.1 | 5 | + | 118716605 | 118747502 | 1547 | 7 | 17 |
| XR_300973.1 | 5 | + | 118899352 | 118904226 | 645 | 4 | 12 |
| XR_303229.1 | 10 | - | 118923080 | 118948281 | 361 | 0 | 9 |
| XM_005665337.1 | 6 | + | 119069137 | 119076597 | 1138 | 116 | 128 |
| XM_005665338.1 | 1 | + | 119069137 | 119076597 | 833 | 116 | 129 |
| XR_304095.1 | 3 | + | 119069137 | 119076597 | 644 | 1 | 3 |
| XR_303230.1 | 3 | - | 119263447 | 119785430 | 788 | 27 | 31 |
| XR_307056.1 | 3 | + | 119724228 | 119772526 | 979 | 1 | 1 |
| XR_307057.1 | 4 | + | 119724228 | 119772526 | 1014 | 1 | 1 |
| XR_307058.1 | 8 | + | 119724228 | 119772526 | 885 | 1 | 2 |
| XR_307059.1 | 8 | + | 119724228 | 119772526 | 975 | 1 | 1 |
| XR_307060.1 | 6 | + | 119724228 | 119772526 | 942 | 1 | 1 |
| XR_307061.1 | 14 | + | 119724228 | 119772526 | 907 | 1 | 1 |
| XR_307062.1 | X | + | 119724228 | 119772526 | 763 | 1 | 1 |
| XR_301950.1 | X | - | 119895438 | 119900551 | 895 | 7 | 12 |
| XR_307790.1 | 6 | - | 120296328 | 120303238 | 842 | 0 | 6 |
| XR_307791.1 | 6 | - | 120296328 | 120303238 | 815 | 1 | 7 |
| XR_307792.1 | 9 | - | 120296328 | 120303238 | 733 | 0 | 6 |
| XR_307793.1 | 9 | - | 120296328 | 120303238 | 865 | 1 | 3 |
| XR_304675.1 | 14 | - | 120363944 | 120379056 | 485 | 3 | 5 |
| XR_304676.1 | X | - | 120363944 | 120379056 | 799 | 5 | 9 |
| XR_307795.1 | 12 | - | 120437564 | 120440267 | 924 | 5 | 6 |
| XR_301956.1 | 4 | + | 120587468 | 120617443 | 534 | 47 | 69 |
| XR_301957.1 | 4 | + | 120587468 | 120617443 | 635 | 52 | 76 |
| XR_301958.1 | 16 | + | 120587468 | 120617443 | 484 | 46 | 63 |
| XR_307800.1 | 16 | + | 120739762 | 120751454 | 755 | 12 | 13 |
| XR_307801.1 | 16 | + | 120739762 | 120751454 | 684 | 14 | 18 |
| XR_307802.1 | 16 | + | 120739762 | 120751454 | 734 | 8 | 11 |
| XR_308244.1 | 12 | - | 120952380 | 121003692 | 1136 | 36 | 25 |
| XR_299313.1 | X | + | 120958386 | 120982406 | 1363 | 139 | 98 |
| XR_299314.1 | 13 | + | 121378573 | 121428800 | 2849 | 350 | 276 |
| XR_299315.1 | 9 | + | 121378573 | 121428800 | 2805 | 347 | 273 |
| XR_307812.1 | 10 | + | 121450126 | 121455242 | 1756 | 24 | 41 |
| XR_307813.1 | 11 | + | 121477509 | 121646659 | 1574 | 17 | 18 |
| XR_307814.1 | 11 | + | 121477509 | 121646659 | 1516 | 16 | 18 |
| XR_307815.1 | 11 | + | 121477509 | 121646659 | 1418 | 15 | 19 |
| NR_021488.1 | 6 | - | 121656253 | 121660495 | 1380 | 516 | 533 |
| XR_297885.1 | 1 | + | 121724884 | 121738242 | 912 | 5 | 18 |
| XR_304707.1 | 1 | - | 121736798 | 121792384 | 652 | 76 | 55 |
| XR_305112.1 | 3 | - | 122079023 | 122115033 | 473 | 25 | 44 |
| XR_299687.1 | 3 | + | 122270027 | 122282517 | 449 | 17 | 6 |
| XR_305698.1 | 3 | + | 122692575 | 122732549 | 534 | 4 | 9 |
| XR_299813.1 | 7 | + | 122931762 | 123001296 | 417 | 1 | 1 |
| XR_299814.1 | 9 | + | 122931762 | 123001296 | 416 | 1 | 1 |
| XM_003135459.2 | 9 | + | 122935361 | 122938090 | 586 | 10 | 11 |
| XR_303244.1 | 9 | - | 123060370 | 123085742 | 1602 | 14 | 21 |
| XR_303245.1 | 9 | - | 123060370 | 123085742 | 1550 | 13 | 21 |
| XR_303248.1 | 9 | - | 123409196 | 123419690 | 1661 | 71 | 48 |
| XR_303249.1 | 15 | - | 123409196 | 123419690 | 1662 | 72 | 49 |
| XR_303250.1 | 6 | - | 123409196 | 123419690 | 1524 | 72 | 49 |
| XR_303251.1 | 8 | - | 123409196 | 123419690 | 1816 | 76 | 56 |
| XR_303252.1 | 10 | - | 123409196 | 123419690 | 1905 | 79 | 57 |
| XR_303253.1 | 3 | - | 123409196 | 123419690 | 1875 | 76 | 56 |
| XR_303254.1 | 7 | - | 123409196 | 123419690 | 1752 | 74 | 49 |
| XR_303255.1 | 7 | - | 123409196 | 123419690 | 1850 | 71 | 50 |
| XR_303256.1 | 2 | - | 123409196 | 123419690 | 1701 | 73 | 50 |
| XR_303257.1 | 13 | - | 123409196 | 123419690 | 1731 | 71 | 49 |
| XR_303258.1 | X | - | 123409196 | 123419690 | 1547 | 68 | 42 |
| XR_303259.1 | 10 | - | 123409196 | 123419690 | 1582 | 70 | 48 |
| XR_309522.1 | 18 | - | 123419532 | 123422447 | 790 | 1 | 2 |
| XR_297968.1 | 1 | + | 123461312 | 123548943 | 1494 | 1307 | 1103 |
| XR_297969.1 | 12 | + | 123461312 | 123548943 | 1554 | 1319 | 1114 |
| XR_297970.1 | 12 | + | 123461312 | 123548943 | 1199 | 1294 | 1098 |
| XR_297971.1 | 13 | + | 123461312 | 123548943 | 1139 | 1282 | 1087 |
| XR_297972.1 | 7 | + | 123461312 | 123548943 | 1463 | 1301 | 1108 |
| XR_297973.1 | 7 | + | 123461312 | 123548943 | 1403 | 1289 | 1097 |
| XR_297974.1 | 5 | + | 123461312 | 123548943 | 2474 | 2083 | 1688 |
| XR_305700.1 | 4 | + | 123472967 | 123485856 | 835 | 4 | 13 |
| XR_305701.1 | 4 | + | 123472967 | 123485856 | 872 | 4 | 11 |
| XR_305702.1 | 6 | + | 123472967 | 123485856 | 819 | 4 | 11 |
| XR_299467.1 | 6 | - | 123541989 | 123545140 | 1751 | 197 | 210 |
| XR_297976.1 | 10 | + | 123650158 | 123672379 | 1652 | 251 | 163 |
| XR_297977.1 | 14 | + | 123650158 | 123672379 | 1404 | 224 | 140 |
| XR_302682.1 | 6 | + | 124009458 | 124040065 | 1501 | 7 | 10 |
| XR_302683.1 | 7 | + | 124009458 | 124040065 | 1479 | 5 | 10 |
| XR_307084.1 | X | + | 124690130 | 124700999 | 681 | 20 | 46 |
| XR_307085.1 | 2 | + | 124690130 | 124700999 | 699 | 20 | 46 |
| XR_299321.1 | 10 | + | 124824568 | 124901819 | 989 | 4 | 5 |
| XR_299322.1 | 11 | + | 124824568 | 124901819 | 904 | 8 | 4 |
| XR_307827.1 | 12 | - | 125226537 | 125262089 | 1007 | 47 | 21 |
| XR_298136.1 | 12 | - | 125364325 | 125507719 | 1395 | 35 | 46 |
| XR_298137.1 | 12 | - | 125364325 | 125507719 | 1578 | 34 | 43 |
| XR_299809.1 | 7 | + | 125522685 | 125528929 | 411 | 2 | 3 |
| XR_298396.1 | 17 | + | 125905766 | 125991206 | 1109 | 14 | 25 |
| XR_298397.1 | 1 | + | 125905766 | 125991206 | 800 | 7 | 13 |
| XR_298398.1 | 6 | + | 126042210 | 126136575 | 515 | 1 | 8 |
| XR_304904.1 | 3 | - | 126548161 | 126563573 | 727 | 1 | 0 |
| XR_302691.1 | 4 | - | 126581257 | 126584115 | 345 | 70 | 48 |
| XR_303283.1 | 4 | + | 126717957 | 126726700 | 1878 | 58 | 52 |
| XM_005663703.1 | 4 | + | 127037316 | 127049681 | 855 | 45 | 44 |
| XR_299847.1 | 4 | + | 127275614 | 127388236 | 720 | 9 | 20 |
| XR_299848.1 | 4 | + | 127275614 | 127388236 | 607 | 6 | 14 |
| XR_302696.1 | 6 | - | 127517872 | 127524648 | 4217 | 383 | 255 |
| XR_307836.1 | 6 | + | 127526551 | 127530189 | 1955 | 74 | 54 |
| XR_307837.1 | 6 | + | 127526551 | 127530189 | 2085 | 91 | 78 |
| XR_307838.1 | 4 | + | 127526551 | 127530189 | 2148 | 94 | 82 |
| XR_307839.1 | 1 | + | 127526551 | 127530189 | 2191 | 93 | 78 |
| XR_307840.1 | 10 | + | 127526551 | 127530189 | 1976 | 74 | 54 |
| XR_307841.1 | 5 | + | 127526551 | 127530189 | 2021 | 75 | 55 |
| XR_307842.1 | 7 | + | 127526551 | 127530189 | 2035 | 75 | 55 |
| XR_307843.1 | 8 | + | 127526551 | 127530189 | 1932 | 74 | 54 |
| XR_302697.1 | 12 | + | 127555564 | 127559321 | 1436 | 19 | 18 |
| XR_307847.1 | 13 | - | 127621858 | 127649806 | 1223 | 312 | 171 |
| XR_302698.1 | 13 | + | 127786369 | 127790877 | 1245 | 2 | 6 |
| XR_302701.1 | 15 | + | 127910226 | 127913990 | 1668 | 0 | 16 |
| XR_301013.1 | 15 | - | 127937026 | 127942424 | 602 | 8 | 4 |
| XR_308259.1 | X | - | 128150047 | 128185645 | 539 | 61 | 94 |
| XR_301525.1 | 6 | - | 128281165 | 128376907 | 542 | 1 | 4 |
| XR_301973.1 | 1 | - | 128318053 | 129486348 | 593 | 4 | 8 |
| XR_301524.1 | 6 | + | 128398747 | 128439696 | 241 | 0 | 3 |
| XR_301975.1 | 5 | + | 128866622 | 128870893 | 551 | 0 | 8 |
| XR_305120.1 | 7 | - | 129238599 | 129247799 | 524 | 3 | 3 |
| XR_300039.1 | 13 | + | 129270880 | 129297004 | 282 | 3 | 14 |
| XR_130524.2 | 1 | - | 129589056 | 129613037 | 1674 | 7 | 14 |
| XR_305123.1 | 8 | + | 130467404 | 130472064 | 475 | 17 | 21 |
| XR_305726.1 | 11 | + | 130834186 | 130919552 | 3101 | 403 | 477 |
| XR_305727.1 | 11 | + | 130834186 | 130919552 | 3119 | 415 | 496 |
| XR_301980.1 | 18 | - | 131046929 | 131118849 | 336 | 9 | 10 |
| XR_307098.1 | 4 | - | 131096980 | 131135579 | 1003 | 138 | 82 |
| XR_307099.1 | 4 | - | 131096980 | 131135579 | 903 | 131 | 78 |
| XR_307100.1 | 7 | - | 131096980 | 131135579 | 992 | 131 | 79 |
| XR_305729.1 | 6 | - | 131182399 | 131201117 | 468 | 13 | 12 |
| XR_305732.1 | 15 | - | 131538595 | 131546909 | 1497 | 116 | 88 |
| XR_305733.1 | 15 | - | 131538595 | 131546909 | 1664 | 126 | 92 |
| XR_305734.1 | 16 | - | 131538595 | 131546909 | 1668 | 126 | 92 |
| XR_305735.1 | 2 | - | 131538595 | 131546909 | 1595 | 124 | 92 |
| XR_305736.1 | 2 | - | 131538595 | 131546909 | 1426 | 110 | 85 |
| XR_116317.2 | 2 | + | 131587116 | 131602771 | 693 | 88 | 61 |
| XM_005667866.1 | 2 | - | 131738051 | 131768168 | 526 | 6 | 9 |
| XR_302717.1 | 14 | - | 131847819 | 131861622 | 1512 | 8 | 17 |
| XR_302718.1 | 14 | - | 131847819 | 131861622 | 1371 | 8 | 17 |
| XR_302719.1 | 14 | - | 131847819 | 131861622 | 1492 | 8 | 17 |
| XR_305742.1 | 14 | - | 131905118 | 131928082 | 521 | 12 | 4 |
| XR_305743.1 | 14 | - | 131905118 | 131928082 | 298 | 6 | 3 |
| XR_305744.1 | 14 | - | 131905118 | 131928082 | 538 | 5 | 2 |
| XR_301982.1 | 14 | + | 131998019 | 132144256 | 1457 | 28 | 35 |
| XR_301032.1 | 5 | - | 132190398 | 132269017 | 508 | 4 | 15 |
| XR_301033.1 | 11 | - | 132190398 | 132269017 | 378 | 4 | 11 |
| XR_308276.1 | 1 | - | 132195947 | 132209469 | 549 | 41 | 38 |
| XR_304108.1 | 5 | + | 132421247 | 132450962 | 914 | 2 | 13 |
| XR_305746.1 | 1 | + | 133141284 | 133144534 | 577 | 153 | 133 |
| XR_305747.1 | 1 | + | 133141284 | 133144534 | 592 | 157 | 133 |
| XR_299088.1 | 1 | + | 133295042 | 133312291 | 2220 | 46 | 32 |
| XR_299089.1 | 9 | - | 133425512 | 133561284 | 878 | 17 | 10 |
| XR_299090.1 | 9 | - | 133425512 | 133561284 | 772 | 13 | 7 |
| XR_307873.1 | 8 | + | 133793362 | 133799601 | 461 | 95 | 84 |
| XR_307874.1 | 11 | + | 133793362 | 133799601 | 395 | 84 | 70 |
| XR_116256.2 | 11 | - | 133969419 | 133970560 | 725 | 1589 | 965 |
| XR_116257.1 | 6 | - | 133969419 | 133970560 | 670 | 1583 | 961 |
| XR_307122.1 | 6 | - | 133969419 | 133970560 | 746 | 1580 | 967 |
| XR_301991.1 | 8 | - | 134361391 | 134402756 | 1030 | 3 | 9 |
| XR_300089.1 | 8 | + | 134491989 | 134493977 | 209 | 0 | 1 |
| XR_301992.1 | 13 | - | 134636582 | 134660659 | 1327 | 33 | 42 |
| XR_301993.1 | 14 | - | 134693295 | 134700923 | 341 | 18 | 17 |
| XR_299093.1 | 2 | + | 134859508 | 134868754 | 631 | 213 | 195 |
| XR_307885.1 | 2 | + | 134865674 | 134956720 | 538 | 1 | 5 |
| XR_299525.1 | 2 | + | 134952187 | 134967448 | 1771 | 14 | 16 |
| XR_298981.1 | 12 | - | 135035179 | 135130438 | 969 | 65 | 46 |
| XR_298982.1 | 1 | - | 135035179 | 135130438 | 976 | 65 | 41 |
| XR_307881.1 | 1 | + | 135093747 | 135097378 | 366 | 3 | 2 |
| XR_297985.1 | 13 | - | 135135636 | 135148641 | 480 | 4 | 4 |
| XR_301995.1 | 1 | + | 135152450 | 135154928 | 819 | 189 | 179 |
| XR_135296.2 | 1 | + | 135420839 | 135429051 | 961 | 898 | 714 |
| XR_298983.1 | 1 | + | 135420839 | 135429051 | 953 | 895 | 713 |
| XR_298984.1 | 3 | + | 135420839 | 135429051 | 966 | 902 | 715 |
| XR_298985.1 | 3 | + | 135420839 | 135429051 | 958 | 899 | 714 |
| XR_301048.1 | 5 | + | 135688649 | 135691660 | 841 | 26 | 20 |
| XR_301052.1 | 1 | - | 135997148 | 136003598 | 486 | 13 | 23 |
| XR_301053.1 | 16 | - | 135997148 | 136003598 | 522 | 10 | 25 |
| XR_307130.1 | 2 | + | 136800693 | 136834051 | 1941 | 295 | 289 |
| XR_307131.1 | 4 | + | 136800693 | 136834051 | 1876 | 285 | 282 |
| XR_307132.1 | 4 | + | 136800693 | 136834051 | 1849 | 292 | 277 |
| XR_307133.1 | 4 | + | 136800693 | 136834051 | 1784 | 282 | 270 |
| XR_298148.1 | 5 | - | 136931805 | 136957532 | 560 | 522 | 387 |
| XR_298150.1 | 8 | - | 137059483 | 137060506 | 279 | 26 | 22 |
| XR_298151.1 | 8 | - | 137059483 | 137060506 | 316 | 126 | 136 |
| XR_298152.1 | 10 | - | 137059483 | 137060506 | 749 | 165 | 156 |
| XR_307138.1 | 10 | + | 137419110 | 137423260 | 563 | 17 | 15 |
| XR_297443.1 | 13 | + | 137582657 | 137591172 | 463 | 0 | 4 |
| XR_297444.1 | 13 | + | 138366907 | 138427178 | 663 | 10 | 4 |
| XR_307903.1 | 14 | - | 138490485 | 138501987 | 5017 | 1224 | 1055 |
| XR_307904.1 | 14 | - | 138490485 | 138501987 | 4860 | 1223 | 1051 |
| XR_305775.1 | 2 | + | 139168724 | 139187290 | 1104 | 258 | 136 |
| XR_297801.1 | 2 | - | 139531952 | 139534113 | 1153 | 48 | 65 |
| XR_297802.1 | 3 | - | 139531952 | 139534113 | 1023 | 44 | 59 |
| XR_297803.1 | 14 | - | 139531952 | 139534113 | 1158 | 47 | 64 |
| XR_297804.1 | X | - | 139531952 | 139534113 | 941 | 41 | 58 |
| XR_297805.1 | 5 | - | 139531952 | 139534113 | 947 | 40 | 57 |
| XR_297806.1 | 7 | - | 139531952 | 139534113 | 867 | 37 | 56 |
| XR_309664.1 | 7 | + | 139714830 | 139752554 | 778 | 6 | 11 |
| XR_307908.1 | 12 | - | 139844136 | 139845539 | 783 | 0 | 5 |
| XR_302003.1 | 15 | - | 140102473 | 140121830 | 297 | 0 | 1 |
| XR_299526.1 | 5 | - | 140165950 | 140172607 | 1354 | 67 | 67 |
| XR_135392.2 | 10 | + | 140969561 | 141003936 | 1089 | 2726 | 2205 |
| XR_307154.1 | 14 | + | 140969561 | 141003936 | 1151 | 2809 | 2273 |
| XR_116319.3 | 14 | + | 141713131 | 141741455 | 4386 | 2635 | 2076 |
| XR_299476.1 | 14 | + | 141713131 | 141741455 | 4472 | 2667 | 2100 |
| XR_298155.1 | 14 | - | 142727675 | 142738939 | 855 | 2 | 10 |
| XR_298156.1 | 18 | - | 142727675 | 142738939 | 689 | 2 | 7 |
| XR_301074.1 | 1 | + | 143505537 | 143512535 | 956 | 110 | 110 |
| XR_297280.1 | 1 | - | 144782179 | 144794628 | 1029 | 13 | 15 |
| XR_299879.1 | 1 | - | 144788426 | 144821610 | 384 | 1 | 0 |
| XR_301533.1 | 2 | + | 145733621 | 145749253 | 359 | 0 | 2 |
| XR_298679.1 | 9 | + | 146085121 | 146133278 | 2482 | 19 | 59 |
| XR_298681.1 | 3 | + | 146264165 | 146378405 | 402 | 2 | 3 |
| XR_300003.1 | 6 | + | 147151740 | 147155001 | 796 | 0 | 1 |
| XR_300004.1 | 6 | + | 147151740 | 147155001 | 742 | 0 | 1 |
| XR_307177.1 | 6 | + | 147417886 | 147443588 | 708 | 39 | 54 |
| XR_307178.1 | 1 | + | 147417886 | 147443588 | 668 | 37 | 51 |
| XR_301093.1 | 18 | - | 147848656 | 147853764 | 1303 | 6 | 17 |
| XR_297993.1 | 18 | - | 147959481 | 147964077 | 493 | 6 | 15 |
| XR_298687.1 | 18 | - | 150117378 | 150123278 | 982 | 36 | 21 |
| XR_298688.1 | 18 | - | 150117378 | 150123278 | 998 | 40 | 20 |
| XM_005654477.1 | 18 | - | 150935404 | 150950493 | 938 | 4 | 11 |
| XR_297287.1 | 18 | - | 151235245 | 151243027 | 2184 | 3 | 12 |
| XR_301109.1 | 4 | - | 152461496 | 152467143 | 697 | 5 | 19 |
| XR_307184.1 | 6 | + | 152468306 | 152532383 | 543 | 0 | 11 |
| XR_301110.1 | 13 | + | 152998582 | 153070342 | 1774 | 54 | 61 |
| XR_301111.1 | 16 | + | 152998582 | 153070342 | 1696 | 54 | 61 |
| XR_301112.1 | 3 | + | 152998582 | 153070342 | 1763 | 54 | 61 |
| XR_301113.1 | 9 | + | 152998582 | 153070342 | 1688 | 49 | 60 |
| XR_301114.1 | 1 | + | 152998582 | 153070342 | 1633 | 52 | 60 |
| XR_301115.1 | 1 | + | 152998582 | 153070342 | 1510 | 46 | 58 |
| XR_304127.1 | 1 | + | 153213252 | 153403889 | 496 | 4 | 2 |
| XR_304161.1 | 1 | + | 155931494 | 155932384 | 733 | 7 | 7 |
| XR_299338.1 | 1 | + | 155987877 | 156000614 | 1387 | 56 | 42 |
| XR_299339.1 | 14 | + | 155987877 | 156000614 | 1382 | 56 | 42 |
| XR_304163.1 | 11 | - | 156121242 | 156322903 | 420 | 3 | 5 |
| XR_304164.1 | 11 | - | 156121242 | 156322903 | 448 | 5 | 5 |
| XM_005665389.1 | 3 | - | 156599855 | 156877335 | 909 | 78 | 58 |
| XR_304166.1 | 10 | + | 156723005 | 156766635 | 354 | 19 | 20 |
| XR_304167.1 | 11 | + | 156723005 | 156766635 | 481 | 41 | 41 |
| XR_304168.1 | 4 | + | 156723005 | 156766635 | 625 | 33 | 24 |
| XR_304169.1 | 4 | + | 156723005 | 156766635 | 633 | 34 | 19 |
| XR_304171.1 | 4 | - | 159015184 | 159020426 | 1975 | 300 | 205 |
| XR_304172.1 | 4 | - | 159015184 | 159020426 | 1823 | 288 | 195 |
| XM_003128006.2 | 11 | - | 159798692 | 159819252 | 776 | 566 | 423 |
| XR_301130.1 | 10 | + | 160153146 | 160483082 | 282 | 0 | 2 |
| XR_304179.1 | 10 | - | 160811174 | 160816349 | 1055 | 24 | 37 |
| XM_005665446.1 | 10 | - | 160985550 | 161098997 | 615 | 14 | 21 |
| XM_005659843.1 | 10 | - | 161878099 | 161986028 | 2441 | 2218 | 2205 |
| XM_005659844.1 | 10 | - | 161878099 | 161986028 | 2445 | 2218 | 2206 |
| XR_301138.1 | 10 | + | 162780371 | 162790356 | 1262 | 10 | 1 |
| XR_301149.1 | 6 | + | 164751946 | 164810399 | 340 | 6 | 5 |
| XR_307201.1 | 12 | + | 165823334 | 165830295 | 463 | 14 | 9 |
| XR_304199.1 | 10 | + | 165834621 | 165843961 | 365 | 18 | 2 |
| XR_298713.1 | 1 | + | 166685555 | 166697232 | 974 | 88 | 45 |
| XR_298714.1 | 6 | + | 166685555 | 166697232 | 1053 | 89 | 45 |
| XR_298715.1 | 4 | + | 166685555 | 166697232 | 922 | 83 | 39 |
| XR_298716.1 | 9 | + | 166685555 | 166697232 | 1001 | 84 | 39 |
| XR_298717.1 | 9 | + | 166685555 | 166697232 | 923 | 88 | 44 |
| XR_298718.1 | 16 | + | 166685555 | 166697232 | 871 | 83 | 38 |
| XR_298719.1 | 7 | - | 167216695 | 167234267 | 884 | 2 | 11 |
| XR_298724.1 | 9 | - | 167364387 | 167366042 | 4469 | 2082 | 1527 |
| XR_298725.1 | 16 | - | 167364387 | 167366042 | 4075 | 2080 | 1515 |
| XR_298726.1 | 16 | - | 167364387 | 167366042 | 4720 | 2104 | 1554 |
| XR_131319.2 | 6 | - | 167523651 | 167541217 | 977 | 8 | 25 |
| XR_300059.1 | 6 | + | 167554287 | 167556989 | 2621 | 568 | 354 |
| XR_304203.1 | 6 | - | 167890677 | 167909445 | 717 | 164 | 156 |
| XR_298729.1 | 7 | + | 169185637 | 169209871 | 825 | 0 | 4 |
| XR_298730.1 | 8 | + | 169185637 | 169209871 | 774 | 0 | 4 |
| XR_298731.1 | 8 | - | 169216236 | 169221373 | 1281 | 46 | 40 |
| XR_299750.1 | 3 | - | 169665110 | 169676127 | 309 | 95 | 96 |
| XR_299751.1 | 3 | - | 169665110 | 169676127 | 494 | 95 | 98 |
| XR_301173.1 | 3 | + | 169915800 | 170621845 | 517 | 17 | 16 |
| XR_300015.1 | 3 | - | 170615238 | 170639287 | 510 | 129 | 94 |
| XR_307223.1 | 3 | + | 176490769 | 176652865 | 492 | 4 | 0 |
| XR_307224.1 | 6 | + | 176490769 | 176652865 | 427 | 4 | 0 |
| XR_300380.1 | 13 | - | 179016401 | 179054096 | 321 | 1 | 0 |
| XR_307225.1 | 3 | + | 179321384 | 179515835 | 754 | 55 | 34 |
| XR_307226.1 | 3 | + | 179321384 | 179515835 | 700 | 55 | 33 |
| XR_307227.1 | 3 | + | 179321384 | 179515835 | 514 | 55 | 29 |
| XR_307228.1 | 9 | + | 179321384 | 179515835 | 851 | 61 | 38 |
| XR_307229.1 | 9 | + | 179321384 | 179515835 | 811 | 55 | 35 |
| XR_307230.1 | 9 | + | 179321384 | 179515835 | 741 | 51 | 30 |
| XR_307231.1 | 15 | + | 179321384 | 179515835 | 649 | 47 | 28 |
| XR_301182.1 | 2 | - | 179780666 | 179800299 | 612 | 12 | 12 |
| XR_307233.1 | 2 | + | 180026597 | 180035340 | 706 | 4 | 5 |
| XR_301185.1 | 2 | + | 180442794 | 180448761 | 406 | 5 | 2 |
| XR_301186.1 | 16 | + | 181097567 | 181315629 | 934 | 0 | 8 |
| XR_301187.1 | 16 | + | 181097567 | 181315629 | 901 | 0 | 8 |
| XR_301188.1 | 11 | + | 181097567 | 181315629 | 1177 | 0 | 9 |
| XR_301189.1 | 12 | + | 181097567 | 181315629 | 842 | 0 | 7 |
| XR_301190.1 | 6 | + | 181097567 | 181315629 | 1216 | 0 | 10 |
| XR_301191.1 | 7 | + | 181097567 | 181315629 | 826 | 2 | 6 |
| XR_301198.1 | 14 | - | 183578380 | 183792458 | 711 | 40 | 33 |
| XR_301199.1 | 9 | - | 183578380 | 183792458 | 682 | 7 | 4 |
| XR_301200.1 | 18 | - | 183578380 | 183792458 | 719 | 14 | 14 |
| XR_297749.1 | 7 | - | 184022135 | 184157161 | 272 | 2 | 1 |
| XR_297750.1 | 7 | - | 184022135 | 184157161 | 362 | 2 | 1 |
| XR_297751.1 | 8 | - | 184022135 | 184157161 | 252 | 2 | 1 |
| XR_301211.1 | 8 | + | 185050335 | 185284136 | 305 | 4 | 4 |
| XR_301214.1 | 8 | + | 186167794 | 186204258 | 1771 | 2 | 15 |
| XR_301215.1 | 13 | + | 186167794 | 186204258 | 1625 | 2 | 16 |
| XR_301216.1 | 14 | + | 186167794 | 186204258 | 1883 | 3 | 15 |
| XR_301217.1 | 14 | + | 186167794 | 186204258 | 1807 | 2 | 15 |
| XR_301218.1 | 11 | + | 186167794 | 186204258 | 1717 | 2 | 14 |
| XR_301219.1 | 4 | + | 186167794 | 186204258 | 1727 | 2 | 16 |
| XR_301220.1 | 3 | + | 186167794 | 186204258 | 1630 | 2 | 15 |
| XR_301221.1 | 17 | + | 186167794 | 186204258 | 1770 | 2 | 15 |
| XR_301222.1 | 17 | + | 186167794 | 186204258 | 1569 | 2 | 16 |
| XR_301223.1 | 17 | + | 186167794 | 186204258 | 1622 | 1 | 15 |
| XR_301235.1 | 17 | - | 187370095 | 187385016 | 578 | 304 | 203 |
| XR_301246.1 | 17 | + | 188486805 | 188576894 | 222 | 6 | 16 |
| XR_301248.1 | 17 | + | 189170582 | 189230278 | 788 | 7 | 5 |
| XR_301249.1 | 17 | + | 189170582 | 189230278 | 768 | 7 | 6 |
| XR_301250.1 | 3 | + | 189170582 | 189230278 | 690 | 6 | 5 |
| XR_300397.1 | 5 | + | 189984928 | 190052000 | 619 | 4 | 3 |
| XR_299358.1 | 5 | + | 191458517 | 191475084 | 601 | 0 | 5 |
| XR_299359.1 | 16 | + | 191458517 | 191475084 | 608 | 1 | 5 |
| XR_298010.1 | 11 | - | 196206992 | 196229454 | 1319 | 309 | 182 |
| XR_299365.1 | 11 | + | 197527249 | 197534016 | 717 | 64 | 70 |
| XR_299366.1 | 6 | + | 197527249 | 197534016 | 746 | 67 | 64 |
| XR_299370.1 | 8 | + | 203254357 | 203273556 | 489 | 25 | 5 |
| XR_307292.1 | 11 | + | 204834894 | 204840384 | 350 | 10 | 12 |
| XR_301295.1 | 11 | - | 206665867 | 206720290 | 1023 | 0 | 25 |
| XR_299371.1 | 10 | - | 207029519 | 207042862 | 722 | 340 | 191 |
| XR_299372.1 | Un | + | 207204848 | 207209292 | 1602 | 1 | 1 |
| XR_301296.1 | 2 | - | 207259375 | 207335278 | 817 | 3 | 4 |
| XR_115703.3 | 2 | - | 208140151 | 208181529 | 1577 | 25 | 41 |
| XR_301302.1 | 2 | + | 209494785 | 209673939 | 649 | 4 | 10 |
| XR_301303.1 | 16 | + | 209494785 | 209673939 | 598 | 4 | 10 |
| XR_301304.1 | 3 | + | 210145558 | 210470044 | 705 | 6 | 6 |
| XR_298013.1 | 3 | + | 211169052 | 211610959 | 363 | 0 | 2 |
| XR_135112.2 | 3 | + | 219295309 | 219385665 | 968 | 39 | 59 |
| XR_301333.1 | 6 | + | 227343818 | 227357264 | 876 | 3 | 8 |
| XR_301335.1 | 7 | - | 231067176 | 231081102 | 391 | 2 | 12 |
| XR_300194.1 | 12 | + | 232539674 | 232818576 | 478 | 2 | 1 |
| XR_300195.1 | 12 | + | 232539674 | 232818576 | 341 | 1 | 1 |
| XR_300196.1 | 12 | + | 232539674 | 232818576 | 474 | 2 | 1 |
| XR_300197.1 | 7 | + | 232539674 | 232818576 | 482 | 2 | 1 |
| XR_301344.1 | 7 | - | 232836151 | 232920769 | 506 | 2 | 9 |
| XR_301348.1 | 10 | + | 232963846 | 233553482 | 663 | 9 | 4 |
| XR_301351.1 | 9 | + | 236673817 | 236694003 | 1948 | 753 | 949 |
| XR_301353.1 | 9 | + | 238333185 | 238338697 | 238 | 4 | 3 |
| XR_301354.1 | 4 | + | 238333185 | 238338697 | 353 | 4 | 3 |
| XR_301356.1 | 1 | - | 238559072 | 238567550 | 680 | 11 | 22 |
| XR_301384.1 | 16 | - | 243216062 | 243225670 | 713 | 0 | 2 |
| XR_301414.1 | 17 | + | 249847810 | 249854068 | 1340 | 6 | 14 |
| XR_301419.1 | 17 | + | 250078525 | 250095163 | 1006 | 79 | 83 |
| XR_301420.1 | 18 | + | 250078525 | 250095163 | 990 | 78 | 83 |
| XR_301421.1 | 18 | + | 250078525 | 250095163 | 876 | 75 | 74 |
| XR_301429.1 | 14 | - | 252035915 | 252127956 | 561 | 1 | 4 |
| XR_301430.1 | 14 | - | 252035915 | 252127956 | 568 | 2 | 4 |
| XR_301462.1 | 7 | - | 255736585 | 255904317 | 657 | 8 | 7 |
| XR_301466.1 | 6 | + | 257844499 | 257884406 | 360 | 1 | 5 |
| XR_301480.1 | 8 | + | 264242212 | 264277538 | 284 | 2 | 1 |
| XR_301495.1 | 8 | - | 268650232 | 268656873 | 520 | 7 | 3 |
| XR_301499.1 | 8 | - | 269050673 | 269056255 | 1627 | 137 | 110 |
| XR_301502.1 | 8 | + | 269575035 | 269607453 | 2369 | 83 | 62 |
| XR_299706.1 | 6 | - | 270190985 | 270219535 | 1301 | 509 | 378 |
| XR_301503.1 | 5 | - | 270636013 | 270652519 | 1048 | 74 | 103 |
| XR_301504.1 | 5 | - | 270636013 | 270652519 | 1776 | 218 | 226 |
| XR_301518.1 | 6 | - | 271260237 | 271271368 | 567 | 3 | 12 |
| XR_298048.1 | 6 | + | 273498257 | 273502695 | 1358 | 809 | 537 |
| XR_298049.1 | 6 | + | 273498257 | 273502695 | 1363 | 809 | 537 |
| XR_298050.1 | 6 | - | 273558443 | 273560170 | 1105 | 33 | 21 |
| XM_005654619.1 | 1 | - | 273618145 | 273634463 | 1099 | 15 | 20 |
| NR_045192.1 | 1 | + | 313848947 | 313854497 | 1064 | 4708 | 5349 |
| NR_045193.1 | 6 | + | 313848947 | 313854497 | 1019 | 4418 | 5036 |

**Table S2: DELs in HF vs EAF**

| LncRNA ID | log2FC | P value | Regulation |
| --- | --- | --- | --- |
| LOC102162053 | 6.26720953 | 3.37E-05 | up |
| LOC102159780 | 3.7912032 | 0.00387502 | up |
| LOC102166802 | 3.70736745 | 0.0007971 | up |
| LOC102158034 | 3.58746555 | 0.02620735 | up |
| LOC102167762 | 3.52628969 | 0.02229751 | up |
| LOC102158292 | 3.45362601 | 0.01254759 | up |
| LOC102165967 | 3.31088684 | 0.03689496 | up |
| LOC102162289 | 3.30809027 | 0.03492543 | up |
| LOC102167506 | 3.20893374 | 0.00316083 | up |
| LOC102163623 | 3.0766483 | 0.04408501 | up |
| LOC102164197 | 3.01907344 | 0.01789587 | up |
| LOC102165594 | 2.98406732 | 0.02921999 | up |
| LOC102161687 | 2.93694551 | 0.0074278 | up |
| LOC102162742 | 2.85506694 | 0.00276197 | up |
| LOC100738196 | 2.84224812 | 0.00359667 | up |
| LOC102159417 | 2.83763797 | 0.02112329 | up |
| LOC102163023 | 2.8267436 | 0.01110264 | up |
| LOC102158351 | 2.78694797 | 0.00194607 | up |
| LOC100520629 | 2.75178334 | 0.00379221 | up |
| LOC102164258 | 2.74623758 | 0.03991284 | up |
| LOC102157842 | 2.73129859 | 0.03514725 | up |
| LOC102161253 | 2.62551246 | 0.04904324 | up |
| LOC102160181 | 2.59206573 | 0.03847667 | up |
| LOC102158650 | 2.57795195 | 0.00353357 | up |
| LOC102157709 | 2.57318854 | 0.0000217 | up |
| LOC102162556 | 2.46336502 | 0.02834863 | up |
| LOC102159120 | 2.45064578 | 0.00043349 | up |
| LOC102160749 | 2.42616495 | 0.03737973 | up |
| LOC102167731 | 2.37802815 | 0.00291944 | up |
| LOC102164613 | 2.28593093 | 0.04916362 | up |
| LOC102166259 | 2.22446579 | 0.00088104 | up |
| LOC102163947 | 2.21795236 | 0.02597088 | up |
| LOC102160834 | 2.20203401 | 0.00676051 | up |
| LOC100620402 | 2.1392284 | 0.02135179 | up |
| LOC102165419 | 2.11199826 | 0.03704005 | up |
| LOC102162931 | 2.10986331 | 0.0408739 | up |
| LOC102162531 | 2.07652793 | 0.04107362 | up |
| LOC100512907 | 2.05594768 | 0.0000216 | up |
| LOC102167986 | 2.03068789 | 0.04775996 | up |
| LOC102160963 | 2.02561678 | 0.01776971 | up |
| LOC102159681 | 1.98351283 | 0.04447436 | up |
| LOC102157477 | 1.93747616 | 0.00064567 | up |
| LOC102159375 | 1.89822979 | 0.04251385 | up |
| LOC102162079 | 1.86253149 | 0.00673743 | up |
| LOC102157546 | 1.84204268 | 0.00590978 | up |
| LOC102167901 | 1.83937796 | 0.00018642 | up |
| LOC102167754 | 1.82120652 | 0.03216522 | up |
| LOC102168017 | 1.78612136 | 0.0145689 | up |
| LOC102159645 | 1.59738249 | 0.00177528 | up |
| LOC102164596 | 1.59606269 | 0.00756901 | up |
| LOC102166296 | 1.53374211 | 0.01349581 | up |
| LOC102160224 | 1.51554767 | 0.03588366 | up |
| LOC102158914 | 1.48516262 | 0.02446616 | up |
| LOC102158991 | 1.48498539 | 0.03779146 | up |
| LOC102163370 | 1.44609783 | 0.01978338 | up |
| LOC102157946 | 1.43871569 | 0.01023442 | up |
| LOC102157642 | 1.38259603 | 0.04634556 | up |
| LOC102160522 | 1.35823036 | 0.02074355 | up |
| LOC102164325 | 1.34341925 | 0.00135266 | up |
| LOC102162338 | 1.29623188 | 0.00536349 | up |
| LOC100622791 | 1.19579362 | 0.00235155 | up |
| LOC102162548 | 1.17877795 | 0.04670894 | up |
| LOC100624137 | 1.09764307 | 0.04806624 | up |
| LOC102165705 | 1.07485581 | 0.04913499 | up |
| LOC102160528 | 1.06431363 | 0.02614064 | up |
| LOC102158455 | 1.04816087 | 0.01950713 | up |
| LOC102163816 | 1.02354211 | 0.02488709 | up |
| LOC100626841 | -1.01286994 | 0.00010342 | down |
| LOC102165622 | -1.07017721 | 0.01434585 | down |
| LOC102162300 | -1.12584172 | 0.00016498 | down |
| LOC102160389 | -1.28857931 | 0.000083 | down |
| LOC102162488 | -1.4822477 | 1.24E-07 | down |
| LOC100524923 | -1.51045242 | 0.00151269 | down |
| LOC102159607 | -1.59326119 | 0.00019935 | down |
| LOC102166913 | -2.02704612 | 0.02122509 | down |
| LOC102167708 | -2.2215703 | 0.04172461 | down |
| LOC102165936 | -2.7496947 | 0.04648052 | down |

**Table S3: cis-target mRNAs of DELs**

| **DELs** | | | | **cis-target mRNAs** | | | |
| --- | --- | --- | --- | --- | --- | --- | --- |
| **lncRNA ID** | **Chr.** | **Start** | **End** | **Gene ID** | **Chr.** | **Start** | **End** |
|  |  |  |  |  |  |  |  |
| LOC102158351 | X | 112510919 | 112947593 | LOC102158723 | X | 112845408 | 112848002 |
| LOC102158351 | X | 112510919 | 112947593 | ZIC3 | X | 112592951 | 112605087 |
| LOC102165967 | X | 19592655 | 19999197 | APOO | X | 19953147 | 20017784 |
| LOC102165967 | X | 19592655 | 19999197 | SAT1 | X | 19908037 | 19910553 |
| LOC102165967 | X | 19592655 | 19999197 | ACOT9 | X | 19835674 | 19864570 |
| LOC102165967 | X | 19592655 | 19999197 | PRDX4 | X | 19800153 | 19819575 |
| LOC102168017 | 17 | 52482410 | 52923182 | NFATC2 | 17 | 52743570 | 52907373 |
| LOC102158455 | 16 | 52581826 | 52991008 | RANBP17 | 16 | 52836291 | 53160156 |
| LOC102158455 | 16 | 52581826 | 52991008 | TLX3 | 16 | 52823839 | 52826717 |
| LOC102158455 | 16 | 52581826 | 52991008 | NPM1 | 16 | 52767263 | 52781862 |
| LOC102158455 | 16 | 52581826 | 52991008 | FGF18 | 16 | 52724867 | 52760246 |
| LOC102159645 | 16 | 46460481 | 47138008 | PIK3R1 | 16 | 46434757 | 46523626 |
| LOC102167708 | 16 | 5970442 | 6414121 | BASP1 | 16 | 6384322 | 6440507 |
| LOC102167708 | 16 | 5970442 | 6414121 | MYO10 | 16 | 5907111 | 6145485 |
| LOC102158914 | 15 | 81604907 | 82006589 | HOXD1 | 15 | 81987618 | 81990026 |
| LOC102158914 | 15 | 81604907 | 82006589 | HOXD3 | 15 | 81969482 | 81974893 |
| LOC102158914 | 15 | 81604907 | 82006589 | LOC110257186 | 15 | 81935837 | 81941383 |
| LOC102158914 | 15 | 81604907 | 82006589 | HOXD8 | 15 | 81930923 | 81935528 |
| LOC102158914 | 15 | 81604907 | 82006589 | HOXD9 | 15 | 81910415 | 81926276 |
| LOC102158914 | 15 | 81604907 | 82006589 | HOXD10 | 15 | 81911372 | 81921164 |
| LOC102158914 | 15 | 81604907 | 82006589 | HOXD11 | 15 | 81906654 | 81910765 |
| LOC102158914 | 15 | 81604907 | 82006589 | HOXD12 | 15 | 81900962 | 81902327 |
| LOC102158914 | 15 | 81604907 | 82006589 | HOXD13 | 15 | 81893997 | 81897965 |
| LOC102158914 | 15 | 81604907 | 82006589 | EVX2 | 15 | 81881362 | 81884994 |
| LOC102158914 | 15 | 81604907 | 82006589 | LNPK | 15 | 81721637 | 81805706 |
| LOC102160389 | 15 | 56082808 | 56566021 | HERC2 | 15 | 56438117 | 56649805 |
| LOC102160389 | 15 | 56082808 | 56566021 | MFHAS1 | 15 | 56200455 | 56296151 |
| LOC102160389 | 15 | 56082808 | 56566021 | ERI1 | 15 | 56091790 | 56123520 |
| LOC102164258 | 15 | 25118181 | 25528679 | TEX51 | 15 | 25469048 | 25471636 |
| LOC102164258 | 15 | 25118181 | 25528679 | BIN1 | 15 | 25328679 | 25379675 |
| LOC102164258 | 15 | 25118181 | 25528679 | LOC100517560 | 15 | 25244585 | 25259545 |
| LOC102164258 | 15 | 25118181 | 25528679 | ERCC3 | 15 | 25189644 | 25224381 |
| LOC102164258 | 15 | 25118181 | 25528679 | MAP3K2 | 15 | 25087479 | 25184106 |
| LOC102160834 | 15 | -199922 | 211743 | STAM2 | 15 | 115297 | 168659 |
| LOC102160834 | 15 | -199922 | 211743 | LOC102167213 | 15 | 100297 | 104346 |
| LOC102162338 | 14 | 54338200 | 54741569 | ACTN2 | 14 | 54670727 | 54742717 |
| LOC102162338 | 14 | 54338200 | 54741569 | MTR | 14 | 54546978 | 54652780 |
| LOC102162338 | 14 | 54338200 | 54741569 | RYR2 | 14 | 53652140 | 54406691 |
| LOC102162531 | 14 | 23474347 | 23878986 | SFSWAP | 14 | 23516253 | 23594252 |
| LOC102162531 | 14 | 23474347 | 23878986 | MMP17 | 14 | 23481220 | 23506936 |
| LOC102166913 | 13 | 203054357 | 203473556 | PCP4 | 13 | 203412479 | 203479216 |
| LOC102166913 | 13 | 203054357 | 203473556 | IGSF5 | 13 | 203325385 | 203372361 |
| LOC102166913 | 13 | 203054357 | 203473556 | B3GALT5 | 13 | 203208353 | 203250085 |
| LOC102166913 | 13 | 203054357 | 203473556 | SH3BGR | 13 | 203046575 | 203108638 |
| LOC102158991 | 13 | 124490130 | 124900999 | ST6GAL1 | 13 | 124699622 | 124837847 |
| LOC102158991 | 13 | 124490130 | 124900999 | ADIPOQ | 13 | 124633906 | 124646237 |
| LOC102158991 | 13 | 124490130 | 124900999 | RFC4 | 13 | 124589292 | 124604421 |
| LOC102158991 | 13 | 124490130 | 124900999 | EIF4A2 | 13 | 124582822 | 124589296 |
| LOC102158991 | 13 | 124490130 | 124900999 | KNG1 | 13 | 124521276 | 124557384 |
| LOC102158991 | 13 | 124490130 | 124900999 | HRG | 13 | 124505305 | 124516387 |
| LOC102158991 | 13 | 124490130 | 124900999 | FETUB | 13 | 124480456 | 124494197 |
| LOC102159375 | 13 | 107507197 | 107932495 | MECOM | 13 | 107753207 | 108336136 |
| LOC102159375 | 13 | 107507197 | 107932495 | LOC100153543 | 13 | 107016526 | 107597398 |
| LOC102157642 | 13 | 75249018 | 75650819 | CEP63 | 13 | 75605622 | 75707087 |
| LOC102157642 | 13 | 75249018 | 75650819 | ANAPC13 | 13 | 75599797 | 75605667 |
| LOC102157642 | 13 | 75249018 | 75650819 | AMOTL2 | 13 | 75490936 | 75507360 |
| LOC102157642 | 13 | 75249018 | 75650819 | RYK | 13 | 75285482 | 75390314 |
| LOC102163370 | 12 | 56128970 | 56530563 | MAP2K4 | 12 | 56402029 | 56516217 |
| LOC102163370 | 12 | 56128970 | 56530563 | ZNF18 | 12 | 56359779 | 56385048 |
| LOC102163370 | 12 | 56128970 | 56530563 | DNAH9 | 12 | 56045079 | 56366327 |
| LOC102166259 | 12 | 24638726 | 25058994 | CALCOCO2 | 12 | 25050008 | 25080230 |
| LOC102166259 | 12 | 24638726 | 25058994 | TTLL6 | 12 | 24980421 | 25033509 |
| LOC102166259 | 12 | 24638726 | 25058994 | HOXB13 | 12 | 24948307 | 24950661 |
| LOC102166259 | 12 | 24638726 | 25058994 | LOC100523635 | 12 | 24889251 | 24901815 |
| LOC102166259 | 12 | 24638726 | 25058994 | HOXB8 | 12 | 24865985 | 24879706 |
| LOC102166259 | 12 | 24638726 | 25058994 | HOXB7 | 12 | 24861165 | 24864675 |
| LOC102166259 | 12 | 24638726 | 25058994 | HOXB6 | 12 | 24849741 | 24853494 |
| LOC102166259 | 12 | 24638726 | 25058994 | HOXB5 | 12 | 24845224 | 24849639 |
| LOC102166259 | 12 | 24638726 | 25058994 | HOXB3 | 12 | 24803119 | 24827557 |
| LOC102166259 | 12 | 24638726 | 25058994 | HOXB2 | 12 | 24796786 | 24800499 |
| LOC102166259 | 12 | 24638726 | 25058994 | HOXB1 | 12 | 24784953 | 24786377 |
| LOC102166259 | 12 | 24638726 | 25058994 | SKAP1 | 12 | 24371699 | 24680280 |
| LOC102162548 | 12 | 23972933 | 24392804 | SKAP1 | 12 | 24371699 | 24680280 |
| LOC102162548 | 12 | 23972933 | 24392804 | SNX11 | 12 | 24351280 | 24360921 |
| LOC102162548 | 12 | 23972933 | 24392804 | CBX1 | 12 | 24319104 | 24351057 |
| LOC102162548 | 12 | 23972933 | 24392804 | NFE2L1 | 12 | 24303381 | 24316765 |
| LOC102162548 | 12 | 23972933 | 24392804 | COPZ2 | 12 | 24280662 | 24292828 |
| LOC102162548 | 12 | 23972933 | 24392804 | CDK5RAP3 | 12 | 24224262 | 24234468 |
| LOC102162548 | 12 | 23972933 | 24392804 | PRR15L | 12 | 24203811 | 24210105 |
| LOC102162548 | 12 | 23972933 | 24392804 | PNPO | 12 | 24192848 | 24201419 |
| LOC102162548 | 12 | 23972933 | 24392804 | SP2 | 12 | 24154087 | 24185898 |
| LOC102162548 | 12 | 23972933 | 24392804 | SP6 | 12 | 24100769 | 24106828 |
| LOC102162548 | 12 | 23972933 | 24392804 | SCRN2 | 12 | 24090685 | 24095163 |
| LOC102162548 | 12 | 23972933 | 24392804 | LRRC46 | 12 | 24083182 | 24089663 |
| LOC102162548 | 12 | 23972933 | 24392804 | MRPL10 | 12 | 24076484 | 24083086 |
| LOC102162548 | 12 | 23972933 | 24392804 | OSBPL7 | 12 | 24059128 | 24075102 |
| LOC102162548 | 12 | 23972933 | 24392804 | TBX21 | 12 | 23996415 | 24009353 |
| LOC102160528 | 12 | 17921672 | 18342760 | DCAKD | 12 | 18337654 | 18363717 |
| LOC102160528 | 12 | 17921672 | 18342760 | NMT1 | 12 | 18303371 | 18337440 |
| LOC102160528 | 12 | 17921672 | 18342760 | PLCD3 | 12 | 18281507 | 18305516 |
| LOC102160528 | 12 | 17921672 | 18342760 | ACBD4 | 12 | 18271835 | 18281639 |
| LOC102160528 | 12 | 17921672 | 18342760 | HEXIM1 | 12 | 18267023 | 18270089 |
| LOC102160528 | 12 | 17921672 | 18342760 | HEXIM2 | 12 | 18251424 | 18263032 |
| LOC102160528 | 12 | 17921672 | 18342760 | FMNL1 | 12 | 18198764 | 18225621 |
| LOC102160528 | 12 | 17921672 | 18342760 | LOC100524336 | 12 | 18194468 | 18198812 |
| LOC102160528 | 12 | 17921672 | 18342760 | SPATA32 | 12 | 18189577 | 18192956 |
| LOC102160528 | 12 | 17921672 | 18342760 | MAP3K14 | 12 | 18142899 | 18182931 |
| LOC102160528 | 12 | 17921672 | 18342760 | ARHGAP27 | 12 | 18037007 | 18069989 |
| LOC102160528 | 12 | 17921672 | 18342760 | PLEKHM1 | 12 | 17984169 | 18035377 |
| LOC102160528 | 12 | 17921672 | 18342760 | LOC100626147 | 12 | 17922278 | 17963486 |
| LOC102167506 | 11 | 71332459 | 71899936 | SLC10A2 | 11 | 71328801 | 71348779 |
| LOC102159417 | 11 | 66877598 | 67333247 | FARP1 | 11 | 67144622 | 67457547 |
| LOC102159417 | 11 | 66877598 | 67333247 | IPO5 | 11 | 67019299 | 67059375 |
| LOC102159417 | 11 | 66877598 | 67333247 | LOC100520401 | 11 | 66967238 | 66967696 |
| LOC102164197 | 11 | 44607715 | 45016252 | MZT1 | 11 | 45000735 | 45019009 |
| LOC102163623 | 11 | 15753097 | 16171119 | WDFY2 | 11 | 16086872 | 16272513 |
| LOC102163623 | 11 | 15753097 | 16171119 | LOC102163801 | 11 | 15977966 | 16088172 |
| LOC102163623 | 11 | 15753097 | 16171119 | LOC106505279 | 11 | 16021127 | 16034650 |
| LOC102163623 | 11 | 15753097 | 16171119 | CCDC70 | 11 | 15976814 | 15978050 |
| LOC102163623 | 11 | 15753097 | 16171119 | ATP7B | 11 | 15892549 | 15942070 |
| LOC102163623 | 11 | 15753097 | 16171119 | ALG11 | 11 | 15851352 | 15876452 |
| LOC102163623 | 11 | 15753097 | 16171119 | NEK5 | 11 | 15799068 | 15862236 |
| LOC102163623 | 11 | 15753097 | 16171119 | NEK3 | 11 | 15772578 | 15798424 |
| LOC102163623 | 11 | 15753097 | 16171119 | CKAP2 | 11 | 15751717 | 15767068 |
| LOC102162556 | 11 | 4494543 | 4931691 | MTIF3 | 11 | 4866017 | 4880000 |
| LOC102162556 | 11 | 4494543 | 4931691 | GTF3A | 11 | 4853305 | 4869297 |
| LOC102162556 | 11 | 4494543 | 4931691 | RASL11A | 11 | 4746408 | 4748895 |
| LOC102162556 | 11 | 4494543 | 4931691 | RPL21 | 11 | 4731747 | 4736177 |
| LOC102162556 | 11 | 4494543 | 4931691 | USP12 | 11 | 4612274 | 4689791 |
| LOC100624137 | 10 | 66233251 | 66637168 | KLF6 | 10 | 66488445 | 66497830 |
| LOC100520629 | 10 | 52226076 | 52679834 | COMMD3 | 10 | 52597391 | 52601376 |
| LOC100520629 | 10 | 52226076 | 52679834 | BMI1 | 10 | 52586089 | 52596154 |
| LOC100520629 | 10 | 52226076 | 52679834 | SPAG6 | 10 | 52496457 | 52571977 |
| LOC100520629 | 10 | 52226076 | 52679834 | PIP4K2A | 10 | 52224367 | 52408928 |
| LOC102164596 | 9 | 92675662 | 93111135 | ABCB1 | 9 | 93049955 | 93146469 |
| LOC102164596 | 9 | 92675662 | 93111135 | LOC100623190 | 9 | 92952551 | 92990833 |
| LOC102164596 | 9 | 92675662 | 93111135 | LOC100522455 | 9 | 92837728 | 92926474 |
| LOC102164596 | 9 | 92675662 | 93111135 | RUNDC3B | 9 | 92525589 | 92823574 |
| LOC102164596 | 9 | 92675662 | 93111135 | LOC100522267 | 9 | 92716340 | 92800942 |
| LOC102164325 | 9 | 39804670 | 40285792 | PLET1 | 9 | 39936355 | 39949709 |
| LOC102164325 | 9 | 39804670 | 40285792 | PTS | 9 | 39920157 | 39927804 |
| LOC102164325 | 9 | 39804670 | 40285792 | BCO2 | 9 | 39864136 | 39916491 |
| LOC102164325 | 9 | 39804670 | 40285792 | TEX12 | 9 | 39864093 | 39869572 |
| LOC102164325 | 9 | 39804670 | 40285792 | IL18 | 9 | 39839959 | 39861258 |
| LOC100738196 | 8 | 113339193 | 114025271 | LOC100518620 | 8 | 114016915 | 114035905 |
| LOC100738196 | 8 | 113339193 | 114025271 | HADH | 8 | 113952561 | 114005510 |
| LOC100738196 | 8 | 113339193 | 114025271 | LEF1 | 8 | 113824511 | 113944078 |
| LOC100738196 | 8 | 113339193 | 114025271 | RPL34 | 8 | 113467779 | 113472058 |
| LOC100738196 | 8 | 113339193 | 114025271 | OSTC | 8 | 113435983 | 113447196 |
| LOC100738196 | 8 | 113339193 | 114025271 | ETNPPL | 8 | 113363047 | 113384783 |
| LOC102159780 | 8 | 19275679 | 19678992 | SEL1L3 | 8 | 19534451 | 19649784 |
| LOC102159780 | 8 | 19275679 | 19678992 | SLC34A2 | 8 | 19481127 | 19507903 |
| LOC102159780 | 8 | 19275679 | 19678992 | LOC100622121 | 8 | 19449340 | 19452545 |
| LOC102159780 | 8 | 19275679 | 19678992 | ANAPC4 | 8 | 19301253 | 19341463 |
| LOC102159780 | 8 | 19275679 | 19678992 | ZCCHC4 | 8 | 19252079 | 19305309 |
| LOC102167901 | 7 | 99932090 | 100339431 | TMEM63C | 7 | 100262732 | 100334300 |
| LOC102167901 | 7 | 99932090 | 100339431 | ZDHHC22 | 7 | 100212946 | 100225540 |
| LOC102167901 | 7 | 99932090 | 100339431 | CIPC | 7 | 100180389 | 100199529 |
| LOC102167901 | 7 | 99932090 | 100339431 | IRF2BPL | 7 | 100119282 | 100123820 |
| LOC102167901 | 7 | 99932090 | 100339431 | LRRC74A | 7 | 99974552 | 100002655 |
| LOC102167901 | 7 | 99932090 | 100339431 | ANGEL1 | 7 | 99929401 | 99959441 |
| LOC102167901 | 7 | 99932090 | 100339431 | VASH1 | 7 | 99910644 | 99933633 |
| LOC102162300 | 7 | 59021849 | 59435214 | PML | 7 | 59405044 | 59450155 |
| LOC102162300 | 7 | 59021849 | 59435214 | ISLR2 | 7 | 59385021 | 59393185 |
| LOC102162300 | 7 | 59021849 | 59435214 | ISLR | 7 | 59349674 | 59352936 |
| LOC102162300 | 7 | 59021849 | 59435214 | STRA6 | 7 | 59317930 | 59347274 |
| LOC102162300 | 7 | 59021849 | 59435214 | LOC100152111 | 7 | 59241409 | 59313171 |
| LOC102162300 | 7 | 59021849 | 59435214 | CCDC33 | 7 | 59188624 | 59224071 |
| LOC102162300 | 7 | 59021849 | 59435214 | CYP11A1 | 7 | 59172829 | 59188479 |
| LOC102162300 | 7 | 59021849 | 59435214 | SEMA7A | 7 | 59094919 | 59119534 |
| LOC102162300 | 7 | 59021849 | 59435214 | UBL7 | 7 | 59070666 | 59084119 |
| LOC102162300 | 7 | 59021849 | 59435214 | LOC100525054 | 7 | 59006838 | 59070598 |
| LOC102167731 | 7 | 57689396 | 58101860 | PTPN9 | 7 | 58078176 | 58164411 |
| LOC102167731 | 7 | 57689396 | 58101860 | SNUPN | 7 | 58038917 | 58065972 |
| LOC102167731 | 7 | 57689396 | 58101860 | IMP3 | 7 | 58023368 | 58024496 |
| LOC102167731 | 7 | 57689396 | 58101860 | SNX33 | 7 | 57999447 | 58015598 |
| LOC102167731 | 7 | 57689396 | 58101860 | CSPG4 | 7 | 57949182 | 57988602 |
| LOC102167731 | 7 | 57689396 | 58101860 | ODF3L1 | 7 | 57935284 | 57939020 |
| LOC102167731 | 7 | 57689396 | 58101860 | LINGO1 | 7 | 57684591 | 57890121 |
| LOC102157546 | 7 | 48899838 | 49350374 | ARNT2 | 7 | 49259118 | 49450471 |
| LOC102157546 | 7 | 48899838 | 49350374 | FAH | 7 | 49047833 | 49087790 |
| LOC102157546 | 7 | 48899838 | 49350374 | ZFAND6 | 7 | 48967402 | 49034711 |
| LOC100622791 | 7 | 24403748 | 24804969 | BTNL2 | 7 | 24789988 | 24809920 |
| LOC100622791 | 7 | 24403748 | 24804969 | LOC100513291 | 7 | 24778319 | 24790468 |
| LOC100622791 | 7 | 24403748 | 24804969 | LOC100513868 | 7 | 24764445 | 24773528 |
| LOC100622791 | 7 | 24403748 | 24804969 | LOC102158214 | 7 | 24725373 | 24759750 |
| LOC100622791 | 7 | 24403748 | 24804969 | LOC106504368 | 7 | 24680986 | 24712081 |
| LOC100622791 | 7 | 24403748 | 24804969 | LOC106504370 | 7 | 24622847 | 24674002 |
| LOC100622791 | 7 | 24403748 | 24804969 | LOC106507398 | 7 | 24517590 | 24520673 |
| LOC100622791 | 7 | 24403748 | 24804969 | TSBP1 | 7 | 24406241 | 24409937 |
| LOC100512907 | 7 | 16746458 | 17554912 | PRL | 7 | 17451367 | 17463771 |
| LOC100512907 | 7 | 16746458 | 17554912 | SOX4 | 7 | 16886834 | 16895488 |
| LOC102165936 | 6 | 165634621 | 166043961 | HPDL | 6 | 166012590 | 166015051 |
| LOC102165936 | 6 | 165634621 | 166043961 | MUTYH | 6 | 166002853 | 166012191 |
| LOC102165936 | 6 | 165634621 | 166043961 | TOE1 | 6 | 165999920 | 166003570 |
| LOC102165936 | 6 | 165634621 | 166043961 | TESK2 | 6 | 165874176 | 166000002 |
| LOC102165936 | 6 | 165634621 | 166043961 | LOC110261410 | 6 | 165865866 | 165870767 |
| LOC102165936 | 6 | 165634621 | 166043961 | MMACHC | 6 | 165860888 | 165865674 |
| LOC102165936 | 6 | 165634621 | 166043961 | PRDX1 | 6 | 165847390 | 165859918 |
| LOC102165936 | 6 | 165634621 | 166043961 | AKR1A1 | 6 | 165811245 | 165825168 |
| LOC102165936 | 6 | 165634621 | 166043961 | NASP | 6 | 165773964 | 165804978 |
| LOC102165936 | 6 | 165634621 | 166043961 | CCDC17 | 6 | 165768569 | 165772763 |
| LOC102165936 | 6 | 165634621 | 166043961 | GPBP1L1 | 6 | 165708964 | 165762392 |
| LOC102165936 | 6 | 165634621 | 166043961 | TMEM69 | 6 | 165704599 | 165708983 |
| LOC102165936 | 6 | 165634621 | 166043961 | IPP | 6 | 165671053 | 165702078 |
| LOC102162079 | 6 | 145885121 | 146333278 | PDE4B | 6 | 146167262 | 146720105 |
| LOC102162079 | 6 | 145885121 | 146333278 | SGIP1 | 6 | 145815658 | 146051621 |
| LOC102164613 | 6 | 100945447 | 101411391 | TMEM200C | 6 | 101211852 | 101217574 |
| LOC102167762 | 6 | 55834838 | 56268799 | MYADM | 6 | 56236306 | 56278577 |
| LOC102167762 | 6 | 55834838 | 56268799 | PRKCG | 6 | 56213479 | 56235683 |
| LOC102167762 | 6 | 55834838 | 56268799 | CACNG7 | 6 | 56188362 | 56211655 |
| LOC102167762 | 6 | 55834838 | 56268799 | CACNG6 | 6 | 56123598 | 56151145 |
| LOC102167762 | 6 | 55834838 | 56268799 | VSTM1 | 6 | 56076296 | 56096581 |
| LOC102167762 | 6 | 55834838 | 56268799 | TARM1 | 6 | 56038524 | 56051487 |
| LOC102167762 | 6 | 55834838 | 56268799 | OSCAR | 6 | 56021434 | 56029398 |
| LOC102167762 | 6 | 55834838 | 56268799 | NDUFA3 | 6 | 56018221 | 56021318 |
| LOC102167762 | 6 | 55834838 | 56268799 | TFPT | 6 | 56011496 | 56018166 |
| LOC102167762 | 6 | 55834838 | 56268799 | PRPF31 | 6 | 55995087 | 56011507 |
| LOC102167762 | 6 | 55834838 | 56268799 | CNOT3 | 6 | 55975628 | 55993140 |
| LOC102167762 | 6 | 55834838 | 56268799 | LENG1 | 6 | 55970585 | 55976740 |
| LOC102167762 | 6 | 55834838 | 56268799 | TMC4 | 6 | 55955955 | 55970265 |
| LOC102167762 | 6 | 55834838 | 56268799 | MBOAT7 | 6 | 55939456 | 55955746 |
| LOC102167762 | 6 | 55834838 | 56268799 | TSEN34 | 6 | 55930726 | 55939487 |
| LOC102167762 | 6 | 55834838 | 56268799 | RPS9 | 6 | 55920930 | 55927280 |
| LOC102167762 | 6 | 55834838 | 56268799 | LOC100622710 | 6 | 55914077 | 55920846 |
| LOC102167762 | 6 | 55834838 | 56268799 | LOC100512905 | 6 | 55828478 | 55846261 |
| LOC100620402 | 6 | 46507828 | 46909227 | WDR87 | 6 | 46890026 | 46901921 |
| LOC100620402 | 6 | 46507828 | 46909227 | LOC102165762 | 6 | 46823849 | 46843085 |
| LOC100620402 | 6 | 46507828 | 46909227 | LOC106510524 | 6 | 46785073 | 46788065 |
| LOC100620402 | 6 | 46507828 | 46909227 | ZFP30 | 6 | 46761838 | 46786290 |
| LOC100620402 | 6 | 46507828 | 46909227 | LOC106510534 | 6 | 46642471 | 46643944 |
| LOC100620402 | 6 | 46507828 | 46909227 | LOC106510532 | 6 | 46585026 | 46613379 |
| LOC100620402 | 6 | 46507828 | 46909227 | LOC102164415 | 6 | 46541335 | 46613318 |
| LOC100620402 | 6 | 46507828 | 46909227 | LOC102164547 | 6 | 46479566 | 46530528 |
| LOC102162742 | 6 | 26266891 | 27119430 | CDH5 | 6 | 27108560 | 27142145 |
| LOC102157946 | 6 | -173749 | 226808 | SPIRE2 | 6 | 211480 | 246109 |
| LOC102157946 | 6 | -173749 | 226808 | TCF25 | 6 | 186068 | 208841 |
| LOC102157946 | 6 | -173749 | 226808 | MC1R | 6 | 181225 | 182187 |
| LOC102157946 | 6 | -173749 | 226808 | DEF8 | 6 | 140854 | 160936 |
| LOC102157946 | 6 | -173749 | 226808 | CENPBD1 | 6 | 139644 | 146306 |
| LOC102157946 | 6 | -173749 | 226808 | LOC100738955 | 6 | 119787 | 139901 |
| LOC102157946 | 6 | -173749 | 226808 | DBNDD1 | 6 | 111053 | 119112 |
| LOC102157946 | 6 | -173749 | 226808 | GAS8 | 6 | 86817 | 105987 |
| LOC102157946 | 6 | -173749 | 226808 | PRDM7 | 6 | 64464 | 78212 |
| LOC102159120 | 5 | 90719800 | 91314826 | BTG1 | 5 | 90815194 | 90817936 |
| LOC100626841 | 5 | 77436229 | 77969907 | RPAP3 | 5 | 77968171 | 78066252 |
| LOC100626841 | 5 | 77436229 | 77969907 | AMIGO2 | 5 | 77642144 | 77645748 |
| LOC100626841 | 5 | 77436229 | 77969907 | SLC38A4 | 5 | 77411610 | 77489542 |
| LOC100626841 | 5 | 77436229 | 77969907 | LOC106510389 | 5 | 77484544 | 77488858 |
| LOC100524923 | 5 | 65257800 | 65672176 | KCNA6 | 5 | 65625581 | 65659557 |
| LOC100524923 | 5 | 65257800 | 65672176 | KCNA1 | 5 | 65556837 | 65565880 |
| LOC100524923 | 5 | 65257800 | 65672176 | KCNA5 | 5 | 65439336 | 65442207 |
| LOC102166296 | 5 | 5862121 | 6269785 | SERHL2 | 5 | 6216323 | 6239811 |
| LOC102166296 | 5 | 5862121 | 6269785 | RRP7A | 5 | 6208495 | 6217381 |
| LOC102166296 | 5 | 5862121 | 6269785 | POLDIP3 | 5 | 6174442 | 6201941 |
| LOC102166296 | 5 | 5862121 | 6269785 | LOC100524254 | 5 | 6146230 | 6170209 |
| LOC102166296 | 5 | 5862121 | 6269785 | A4GALT | 5 | 6083793 | 6105921 |
| LOC102166296 | 5 | 5862121 | 6269785 | ARFGAP3 | 5 | 5994137 | 6050543 |
| LOC102166296 | 5 | 5862121 | 6269785 | PACSIN2 | 5 | 5853377 | 5985174 |
| LOC102160181 | 5 | 3727264 | 4136743 | UPK3A | 5 | 4109985 | 4122272 |
| LOC102160181 | 5 | 3727264 | 4136743 | FAM118A | 5 | 4065393 | 4102584 |
| LOC102160181 | 5 | 3727264 | 4136743 | SMC1B | 5 | 3976098 | 4067148 |
| LOC102160181 | 5 | 3727264 | 4136743 | RIBC2 | 5 | 3966339 | 3976024 |
| LOC102160181 | 5 | 3727264 | 4136743 | FBLN1 | 5 | 3839498 | 3927178 |
| LOC102160181 | 5 | 3727264 | 4136743 | AFUA_5G15080 | 5 | 3906826 | 3907128 |
| LOC102160181 | 5 | 3727264 | 4136743 | ATXN10 | 5 | 3637794 | 3798204 |
| LOC102165594 | 4 | 110026328 | 110468209 | LOC780435 | 4 | 110451442 | 110457254 |
| LOC102165594 | 4 | 110026328 | 110468209 | LOC110260351 | 4 | 110440700 | 110446165 |
| LOC102165594 | 4 | 110026328 | 110468209 | LOC110260348 | 4 | 110427855 | 110433796 |
| LOC102165594 | 4 | 110026328 | 110468209 | LOC110260350 | 4 | 110415235 | 110424911 |
| LOC102165594 | 4 | 110026328 | 110468209 | LOC106510200 | 4 | 110378016 | 110413483 |
| LOC102165594 | 4 | 110026328 | 110468209 | GSTM3 | 4 | 110330027 | 110332957 |
| LOC102165594 | 4 | 110026328 | 110468209 | EPS8L3 | 4 | 110311533 | 110326999 |
| LOC102165594 | 4 | 110026328 | 110468209 | CSF1 | 4 | 110178466 | 110198232 |
| LOC102165594 | 4 | 110026328 | 110468209 | AHCYL1 | 4 | 110092976 | 110132826 |
| LOC102165594 | 4 | 110026328 | 110468209 | STRIP1 | 4 | 110061596 | 110081017 |
| LOC102165594 | 4 | 110026328 | 110468209 | ALX3 | 4 | 110047254 | 110058205 |
| LOC102160224 | 4 | 100883811 | 101290824 | LOC102159652 | 4 | 101285123 | 101301055 |
| LOC102160224 | 4 | 100883811 | 101290824 | LOC102159389 | 4 | 101249917 | 101280702 |
| LOC102160224 | 4 | 100883811 | 101290824 | LOC102164472 | 4 | 101240206 | 101249911 |
| LOC102160224 | 4 | 100883811 | 101290824 | LOC106505207 | 4 | 101195806 | 101219254 |
| LOC102160224 | 4 | 100883811 | 101290824 | NOTCH2 | 4 | 100951522 | 101152348 |
| LOC102160224 | 4 | 100883811 | 101290824 | LOC100157002 | 4 | 100880843 | 100900942 |
| LOC102162289 | 4 | 81887618 | 82311961 | LOC106510102 | 4 | 82291313 | 82387381 |
| LOC102162289 | 4 | 81887618 | 82311961 | DPT | 4 | 82287657 | 82319380 |
| LOC102162289 | 4 | 81887618 | 82311961 | ATP1B1 | 4 | 81912543 | 81937015 |
| LOC102162289 | 4 | 81887618 | 82311961 | NME7 | 4 | 81639907 | 81912276 |
| LOC102167986 | 4 | 10520719 | 10988751 | LOC100151870 | 4 | 10724754 | 10744226 |
| LOC102167986 | 4 | 10520719 | 10988751 | LOC110260220 | 4 | 10714906 | 10717293 |
| LOC102167986 | 4 | 10520719 | 10988751 | LOC110260219 | 4 | 10684969 | 10695247 |
| LOC102167986 | 4 | 10520719 | 10988751 | LOC102165484 | 4 | 10648286 | 10665334 |
| LOC102167986 | 4 | 10520719 | 10988751 | CYRIB | 4 | 10476137 | 10643395 |
| LOC102157709 | 3 | 110767442 | 111168685 | BABAM2 | 3 | 110897250 | 111328414 |
| LOC102157709 | 3 | 110767442 | 111168685 | FOSL2 | 3 | 110820403 | 110842903 |
| LOC102160749 | 3 | 93470769 | 93881093 | SOCS5 | 3 | 93777402 | 93925859 |
| LOC102160749 | 3 | 93470769 | 93881093 | MCFD2 | 3 | 93620921 | 93633018 |
| LOC102160749 | 3 | 93470769 | 93881093 | TTC7A | 3 | 93457623 | 93591982 |
| LOC102167754 | 3 | 80872355 | 81297857 | BCL11A | 3 | 81195217 | 81296165 |
| LOC102167754 | 3 | 80872355 | 81297857 | PAPOLG | 3 | 80986395 | 81025369 |
| LOC102167754 | 3 | 80872355 | 81297857 | REL | 3 | 80861862 | 80912233 |
| LOC102160963 | 3 | 76967358 | 77392315 | AFTPH | 3 | 77351872 | 77422854 |
| LOC102160963 | 3 | 76967358 | 77392315 | SERTAD2 | 3 | 77192570 | 77317327 |
| LOC102160963 | 3 | 76967358 | 77392315 | SLC1A4 | 3 | 76938102 | 76970519 |
| LOC102165419 | 3 | 10889321 | 11391336 | RFC2 | 3 | 11373489 | 11392690 |
| LOC102165419 | 3 | 10889321 | 11391336 | LAT2 | 3 | 11353213 | 11372100 |
| LOC102165419 | 3 | 10889321 | 11391336 | EIF4H | 3 | 11321486 | 11348473 |
| LOC102165419 | 3 | 10889321 | 11391336 | LIMK1 | 3 | 11252820 | 11279286 |
| LOC102165419 | 3 | 10889321 | 11391336 | ELN | 3 | 11214194 | 11245891 |
| LOC102163947 | 3 | 10735613 | 11148922 | TMEM270 | 3 | 11059177 | 11079858 |
| LOC102165419 | 3 | 10889321 | 11391336 | TMEM270 | 3 | 11059177 | 11079858 |
| LOC102163947 | 3 | 10735613 | 11148922 | METTL27 | 3 | 11054822 | 11057734 |
| LOC102165419 | 3 | 10889321 | 11391336 | METTL27 | 3 | 11054822 | 11057734 |
| LOC102163947 | 3 | 10735613 | 11148922 | CLDN4 | 3 | 11039711 | 11053930 |
| LOC102165419 | 3 | 10889321 | 11391336 | CLDN4 | 3 | 11039711 | 11053930 |
| LOC102163947 | 3 | 10735613 | 11148922 | CLDN3 | 3 | 11019073 | 11020102 |
| LOC102165419 | 3 | 10889321 | 11391336 | CLDN3 | 3 | 11019073 | 11020102 |
| LOC102163947 | 3 | 10735613 | 11148922 | LOC106509660 | 3 | 10991944 | 11004714 |
| LOC102165419 | 3 | 10889321 | 11391336 | LOC106509660 | 3 | 10991944 | 11004714 |
| LOC102163947 | 3 | 10735613 | 11148922 | ABHD11 | 3 | 11000991 | 11003789 |
| LOC102165419 | 3 | 10889321 | 11391336 | ABHD11 | 3 | 11000991 | 11003789 |
| LOC102163947 | 3 | 10735613 | 11148922 | STX1A | 3 | 10974127 | 10991846 |
| LOC102165419 | 3 | 10889321 | 11391336 | STX1A | 3 | 10974127 | 10991846 |
| LOC102163947 | 3 | 10735613 | 11148922 | BUD23 | 3 | 10960037 | 10973084 |
| LOC102165419 | 3 | 10889321 | 11391336 | BUD23 | 3 | 10960037 | 10973084 |
| LOC102163947 | 3 | 10735613 | 11148922 | DNAJC30 | 3 | 10951173 | 10959935 |
| LOC102165419 | 3 | 10889321 | 11391336 | DNAJC30 | 3 | 10951173 | 10959935 |
| LOC102163947 | 3 | 10735613 | 11148922 | VPS37D | 3 | 10947403 | 10951271 |
| LOC102165419 | 3 | 10889321 | 11391336 | VPS37D | 3 | 10947403 | 10951271 |
| LOC102163947 | 3 | 10735613 | 11148922 | MLXIPL | 3 | 10898675 | 10934257 |
| LOC102165419 | 3 | 10889321 | 11391336 | MLXIPL | 3 | 10898675 | 10934257 |
| LOC102163947 | 3 | 10735613 | 11148922 | TBL2 | 3 | 10850347 | 10892441 |
| LOC102165419 | 3 | 10889321 | 11391336 | TBL2 | 3 | 10850347 | 10892441 |
| LOC102163947 | 3 | 10735613 | 11148922 | BCL7B | 3 | 10850347 | 10887485 |
| LOC102163947 | 3 | 10735613 | 11148922 | BAZ1B | 3 | 10761461 | 10843918 |
| LOC102163947 | 3 | 10735613 | 11148922 | FZD9 | 3 | 10753294 | 10762812 |
| LOC102158292 | 3 | 3182570 | 3678292 | RADIL | 3 | 3642294 | 3716139 |
| LOC102158292 | 3 | 3182570 | 3678292 | AP5Z1 | 3 | 3622684 | 3642370 |
| LOC102158292 | 3 | 3182570 | 3678292 | FOXK1 | 3 | 3556139 | 3621150 |
| LOC102158292 | 3 | 3182570 | 3678292 | SDK1 | 3 | 2814418 | 3327838 |
| LOC102165705 | 2 | 91702465 | 92322360 | EDIL3 | 2 | 92122495 | 92554351 |
| LOC102165705 | 2 | 91702465 | 92322360 | HAPLN1 | 2 | 91857495 | 91935485 |
| LOC102165705 | 2 | 91702465 | 92322360 | VCAN | 2 | 91682177 | 91811674 |
| LOC102163816 | 2 | 6549941 | 6994116 | SLC22A20P | 2 | 6969878 | 6996239 |
| LOC102163816 | 2 | 6549941 | 6994116 | POLA2 | 2 | 6922384 | 6950289 |
| LOC102163816 | 2 | 6549941 | 6994116 | CDC42EP2 | 2 | 6897497 | 6904040 |
| LOC102163816 | 2 | 6549941 | 6994116 | DPF2 | 2 | 6870331 | 6886548 |
| LOC102163816 | 2 | 6549941 | 6994116 | TIGD3 | 2 | 6865008 | 6867940 |
| LOC102163816 | 2 | 6549941 | 6994116 | SLC25A45 | 2 | 6851101 | 6857886 |
| LOC102163816 | 2 | 6549941 | 6994116 | FRMD8 | 2 | 6822964 | 6843941 |
| LOC102163816 | 2 | 6549941 | 6994116 | SCYL1 | 2 | 6720047 | 6733477 |
| LOC102163816 | 2 | 6549941 | 6994116 | LTBP3 | 2 | 6701455 | 6720191 |
| LOC102163816 | 2 | 6549941 | 6994116 | ZNRD2 | 2 | 6680832 | 6684564 |
| LOC102163816 | 2 | 6549941 | 6994116 | FAM89B | 2 | 6680832 | 6682506 |
| LOC102163816 | 2 | 6549941 | 6994116 | EHBP1L1 | 2 | 6662460 | 6678721 |
| LOC102163816 | 2 | 6549941 | 6994116 | KCNK7 | 2 | 6657841 | 6662445 |
| LOC102163816 | 2 | 6549941 | 6994116 | MAP3K11 | 2 | 6640061 | 6657717 |
| LOC102163816 | 2 | 6549941 | 6994116 | PCNX3 | 2 | 6619213 | 6639933 |
| LOC102163816 | 2 | 6549941 | 6994116 | SIPA1 | 2 | 6606613 | 6618556 |
| LOC102163816 | 2 | 6549941 | 6994116 | RELA | 2 | 6594869 | 6602684 |
| LOC102163816 | 2 | 6549941 | 6994116 | KAT5 | 2 | 6560544 | 6572461 |
| LOC102163816 | 2 | 6549941 | 6994116 | RNASEH2C | 2 | 6563093 | 6564281 |
| LOC102163816 | 2 | 6549941 | 6994116 | AP5B1 | 2 | 6538784 | 6551337 |
| LOC102165622 | 2 | 5955874 | 6366353 | SART1 | 2 | 6355690 | 6373342 |
| LOC102165622 | 2 | 5955874 | 6366353 | EIF1AD | 2 | 6334882 | 6340125 |
| LOC102165622 | 2 | 5955874 | 6366353 | BANF1 | 2 | 6333451 | 6335326 |
| LOC102165622 | 2 | 5955874 | 6366353 | CST6 | 2 | 6323737 | 6325519 |
| LOC102165622 | 2 | 5955874 | 6366353 | CATSPER1 | 2 | 6312906 | 6321690 |
| LOC102165622 | 2 | 5955874 | 6366353 | GAL3ST3 | 2 | 6297797 | 6306200 |
| LOC102165622 | 2 | 5955874 | 6366353 | SF3B2 | 2 | 6279039 | 6295550 |
| LOC102165622 | 2 | 5955874 | 6366353 | PACS1 | 2 | 6138345 | 6277309 |
| LOC102165622 | 2 | 5955874 | 6366353 | KLC2 | 2 | 6117236 | 6127048 |
| LOC102165622 | 2 | 5955874 | 6366353 | RAB1B | 2 | 6110438 | 6116443 |
| LOC102165622 | 2 | 5955874 | 6366353 | CNIH2 | 2 | 6102347 | 6109424 |
| LOC102165622 | 2 | 5955874 | 6366353 | YIF1A | 2 | 6097749 | 6101965 |
| LOC102165622 | 2 | 5955874 | 6366353 | TMEM151A | 2 | 6090535 | 6095499 |
| LOC102165622 | 2 | 5955874 | 6366353 | CD248 | 2 | 6070885 | 6073518 |
| LOC102165622 | 2 | 5955874 | 6366353 | RIN1 | 2 | 6053875 | 6061051 |
| LOC102165622 | 2 | 5955874 | 6366353 | BRMS1 | 2 | 6046077 | 6053672 |
| LOC102165622 | 2 | 5955874 | 6366353 | B4GAT1 | 2 | 6043594 | 6045220 |
| LOC102165622 | 2 | 5955874 | 6366353 | SLC29A2 | 2 | 6027287 | 6036280 |
| LOC102165622 | 2 | 5955874 | 6366353 | NPAS4 | 2 | 5990936 | 5995940 |
| LOC102165622 | 2 | 5955874 | 6366353 | MRPL11 | 2 | 5978470 | 5981449 |
| LOC102165622 | 2 | 5955874 | 6366353 | PELI3 | 2 | 5950552 | 5959786 |
| LOC102161253 | 1 | 230867176 | 231281102 | LOC106509192 | 1 | 231195851 | 231271453 |
| LOC102161253 | 1 | 230867176 | 231281102 | PSAT1 | 1 | 231142673 | 231172364 |
| LOC102161253 | 1 | 230867176 | 231281102 | CEP78 | 1 | 231081157 | 231113065 |
| LOC102161253 | 1 | 230867176 | 231281102 | GNAQ | 1 | 230607469 | 230906988 |
| LOC102161687 | 1 | 185967794 | 186404258 | EXOC5 | 1 | 186376134 | 186430206 |
| LOC102161687 | 1 | 185967794 | 186404258 | OTX2 | 1 | 186028575 | 186039026 |
| LOC102162931 | 1 | 131990398 | 132469017 | LOC110259514 | 1 | 132021037 | 132022658 |
| LOC102163023 | 1 | 108741524 | 109152743 | TPM1 | 1 | 108986981 | 109016090 |
| LOC102163023 | 1 | 108741524 | 109152743 | LACTB | 1 | 108909690 | 108924509 |
| LOC102163023 | 1 | 108741524 | 109152743 | RPS27L | 1 | 108881330 | 108886334 |
| LOC102163023 | 1 | 108741524 | 109152743 | RAB8B | 1 | 108772851 | 108840222 |
| LOC102163023 | 1 | 108741524 | 109152743 | APH1B | 1 | 108549297 | 108763279 |
| LOC102166802 | 1 | 58503080 | 58974661 | MAP3K7 | 1 | 58482046 | 58548087 |
| LOC102159681 | 1 | 16619952 | 17043000 | UST | 1 | 16840422 | 17138088 |

**Table S4: GO terms for DELs**

| Category | ID | Count | Rate | Genes | PValue |
| --- | --- | --- | --- | --- | --- |
| BP | embryonic skeletal system morphogenesis | 11 | 3.28358209 | HOXB3, HOXB2, HOXB1, HOXD3, HOXB8, BMI1, HOXD9, HOXB7, HOXD10, HOXB6, HOXB5 | 3.50E-11 |
| BP | anterior/posterior pattern specification | 13 | 3.88059701 | HOXD13, HOXD10, ZIC3, HOXB3, HOXB2, HOXB1, HOXD3, HOXB8, HOXD9, HOXB7, HOXB6, HOXD8, HOXB5 | 1.89E-10 |
| MF | sequence-specific DNA binding | 16 | 4.7761194 | HOXD1, BCL11A, LEF1, HOXD12, EVX2, HOXB13, BMI1, HOXD10, FOSL2, TLX3, HOXB2, HOXB1, HOXD3, HOXB8, HOXB7, HOXB6 | 9.24E-05 |
| CC | nucleoplasm | 29 | 8.65671642 | NOTCH2, SF3B2, AP5Z1, TESK2, HOXB13, MRPL10, IPO5, IRF2BPL, TFPT, PAPOLG, DPF2, PNPO, PACSIN2, HADH, SOX4, HEXIM1, MAP3K2, HEXIM2, NPM1, BCL11A, NFATC2, ARHGAP27, EVX2, AFTPH, POLA2, NASP, HOXD3, STAM2, HOXB7 | 0.0054083 |
| MF | cysteine-type endopeptidase inhibitor activity | 4 | 1.19402985 | FETUB, HRG, CST6, KNG1 | 0.00641989 |
| BP | skeletal system development | 5 | 1.49253731 | VCAN, HOXD13, HOXD12, SOX4, HAPLN1 | 0.0066041 |
| MF | transcription factor activity, sequence-specific DNA binding | 15 | 4.47761194 | NFATC2, HOXD10, RELA, FOSL2, MLXIPL, MECOM, TBX21, HOXB3, HOXB2, HOXD3, HOXB8, HOXB7, SOX4, NFE2L1, HOXB6 | 0.01232205 |
| BP | positive regulation of transcription from RNA polymerase II promoter | 16 | 4.7761194 | CKAP2, HOXD10, RELA, IRF2BPL, NPAS4, MLXIPL, MC1R, ERCC3, KAT5, REL, HOXB1, HOXD9, SKAP1, SOX4, NFE2L1, CDK5RAP3 | 0.01470139 |
| MF | protein kinase activity | 7 | 2.08955224 | MAP3K2, RYK, NEK5, TESK2, NEK3, MAP3K14, SCYL1 | 0.01637301 |
| CC | nucleus | 52 | 15.5223881 | PRDM7, BTG1, BMI1, BASP1, ZIC3, PAPOLG, PIP4K2A, NEK3, CDK5RAP3, SOX4, PRKCG, ZNF18, ANGEL1, GTF3A, NPAS4, HOXB3, HOXB2, HOXB1, HOXB8, SKAP1, HOXB6, HOXB5, HOXD1, RPS27L, HOXD13, FOXK1, HOXD12, HOXD10, RELA, EIF1AD, PRDX4, SERTAD2, PRDX1, TBX21, ALX3, OTX2, MUTYH, HEXIM1, HEXIM2, RYK, SPAG6, BCL11A, EVX2, SNUPN, MLXIPL, TLX3, LOC102167213, CNOT3, SP6, REL, HOXD9, HOXD8 | 0.01797517 |
| MF | RNA polymerase II core promoter proximal region sequence-specific DNA binding | 9 | 2.68656716 | NPAS4, MLXIPL, ZIC3, BCL11A, LEF1, DPF2, NFATC2, HOXD13, OTX2 | 0.02177794 |
| BP | angiogenesis | 6 | 1.79104478 | FGF18, IL18, HOXB3, VASH1, HOXB13, PLCD3 | 0.02944843 |
| BP | negative regulation of transcription from RNA polymerase II promoter | 12 | 3.58208955 | HEXIM1, HEXIM2, BRMS1, KAT5, BCL11A, LEF1, REL, HOXB3, BMI1, RELA, HOXD8, IRF2BPL | 0.03081342 |
| BP | rhombomere 4 development | 2 | 0.59701493 | HOXB2, HOXB1 | 0.03643346 |
| BP | positive regulation of signal transduction by p53 class mediator | 2 | 0.59701493 | HEXIM1, CDK5RAP3 | 0.03643346 |
| MF | transcriptional activator activity | 7 | 2.08955224 | NPAS4, ZIC3, LEF1, NFATC2, HOXD13, OTX2, SOX4 | 0.03684364 |

**Table S5: KEGG pathway analysis for DELs**

| Term | Count | Rate | Genes | PValue |
| --- | --- | --- | --- | --- |
| MAPK signaling pathway | 10 | 2.98507463 | PRKCG, MAP3K2, CACNG6, CACNG7, MAP2K4, MECOM, FGF18, MAP3K7, MAP3K14, RELA | 0.0113604 |
| Osteoclast differentiation | 7 | 2.08955224 | CSF1, NFATC2, MAP3K7, MAP3K14, RELA, OSCAR, FOSL2 | 0.01522422 |
| Cardiac muscle contraction | 5 | 1.49253731 | CACNG6, CACNG7, RYR2, TPM1, ATP1B1 | 0.02433747 |
| Insulin secretion | 5 | 1.49253731 | PRKCG, RYR2, GNAQ, ATP1B1, STX1A | 0.0395089 |
| Adrenergic signaling in cardiomyocytes | 6 | 1.79104478 | CACNG6, CACNG7, RYR2, GNAQ, TPM1, ATP1B1 | 0.04683733 |
| Melanogenesis | 5 | 1.49253731 | PRKCG, MC1R, GNAQ, LEF1, FZD9 | 0.05205057 |
| Ribosome | 6 | 1.79104478 | RPS9, RPL21, RPL34, RPS27L, MRPL10, MRPL11 | 0.06376991 |
| Arrhythmogenic right ventricular cardiomyopathy (ARVC) | 4 | 1.19402985 | CACNG6, CACNG7, RYR2, LEF1 | 0.06716028 |
| Oxytocin signaling pathway | 6 | 1.79104478 | PRKCG, CACNG6, CACNG7, RYR2, GNAQ, NFATC2 | 0.07674827 |
| TNF signaling pathway | 5 | 1.49253731 | MAP2K4, CSF1, MAP3K7, MAP3K14, RELA | 0.0807366 |
| African trypanosomiasis | 3 | 0.89552239 | PRKCG, GNAQ, IL18 | 0.09319096 |
| DNA replication | 3 | 0.89552239 | RNASEH2C, POLA2, RFC2 | 0.09789708 |

**Table S6: Primers used in this study**

| **Usage** | **Genes/Name** | **Forward primer** | **Reverse primer** |
| --- | --- | --- | --- |
| **Real-time PCR** | NORHA | TCATCCTGCAGCCCTCATTATAC | CCTTCTCAACCCAACTCGCTATT |
|  | FoxO1 | GCAAATCGAGTTACGGAGGC | AATGTCATTATGGGGAGGAGAGT |
|  | caspase 3 | ATGCTGCAAATCTCAGGGAGACCT | CACCATGGCTTAGAAGCACGCAAA |
|  | miR-96 | TTTGGCACTAGCACATTTTTGCT |  |
|  | miR-182 | TTTGGCAATGGTAGAACTCACACT |  |
|  | miR-183 | TATGGCACTGGTAGAATTCACTG |  |
|  | LOC102162053 | TGAAAACATCTGCCATTGTGACTA | AGCACCCAATAAGTGGTACCTTAC |
|  | LOC100512907 | CGGAGTTTGTTGAAAGCGTGAT | ATCCAGGAATGACTCGATGTTGTAT |
|  | LOC102157709 | CAAAGGAGTTGTGCTGTCCAGTC | GGTTGTTCCCCACGTCTTCAG |
|  | LOC102159120 | CTGGAACTTTCAACAGTGTGTGAGT | CACAAAGTCTGCTATTTGAGGAATG |
|  | LOC102162488 | TCCCCAGATTGGTCGTTGTTC | CACAGGCAATAGGAAGCGAGTC |
|  | LOC102160389 | CCCTTCTGCTGATTTGAATACAC | AGGTTCTTCATTCCTCCACACA |
|  | U6 | GGCAAGGATGACACGCAAAT |  |
|  | GAPDH | GGACTCATGACCACGGTCCAT | TCAGATCCACAACCGACACGT |
| **Vector**  **construction** | pcDNA3.1-NORHA | CAGCCCACAGCATTTCTCTCTC | CCTTCTCAACCCAACTCGCTAT |
|  | prim-GLO-FoxO1 WT | GCAGGTTATGTGCTGCTGTAGA | CTTTCCTTGGACCAAATGGATA |
|  | prim-GLO-NORHA WT | GGAAAGGTAAATGTGAAACGTGTC | GCACTTTTAGGGGTAAGACCACTA |
|  | prim-GLO-FoxO1 Mut | TGTGCCATTGGGAATTTCATTACAATGAAGTGAACAACTCACTA | ATACACGACGACATCTATTCCTGACACGGTAACCCTTAAAGTAA |
|  | prim-GLO-NORHA Mut | ATGAAATATGCTTTGGGCTTGCAGGAGCCCAGCAGCTGCAAGAG | AGCCCAAAGCATATTTCATGGTGGTGATTCCTTTCCCAGG |
| **RACE** | NORHA 5’ RACE SP | CAGCCTCCCCCCACCCCAACTTCAG |  |
|  | NORHA 3’ RACE SP | GCTGCCTGGATTTTCTGGGGAAGGT |  |

**Table S7: Oligonucleotides used in this study**

| **Name** | **Sense（5'-3'）** | **Antisense（5'-3'）** |
| --- | --- | --- |
| NORHA-siRNA | GGAAAUAGCGAGUUGGGUUTT | AACCCAACUCGCUAUUUCCTT |
| FoxO1-siRNA | GCGGCAAAGAUGGCUUCUATT | UAGAAGCCAUCUUUGCCGCTT |
| miR-96 mimics | UUUGGCACUAGCACAUUUUUGCU |  |
| miR-182 mimics | UUUGGCAAUGGUAGAACUCACACU |  |
| miR-183 mimics | UAUGGCACUGGUAGAAUUCACUG |  |
| miR-96 inhibitor | AGCAAAAAUGUGCUAGUGCCAAA |  |
| miR-182 inhibitor | AGUGUGAGUUCUACCAUUGCCAAA |  |
| miR-183 inhibitor | CAGUGAAUUCUACCAGUGCCAUA |  |
