# Supplementary material for: NORHA, a novel follicular atresia-related lncRNA, promotes porcine granulosa cell apoptosis via the miR-183-96-182 cluster and FoxO1 axis
Source: J Anim Sci Biotechnol. 2021 Oct 7;12:103. doi: 10.1186/s40104-021-00626-7 (PMC8495971; doi:10.1186/s40104-021-00626-7)
Supplement: Supplementary file 2 — Additional file 2: Figure S1. Overview of experiment design. Flow diagram showing the experimental design and methods. The intermittent blue arrow represents the indirect regulation between NORHA and FoxO1. Figure S2. Gene ontology (GO) analysis. GO functional enrichment of the cis-target mRNAs for all DELs was performed by an online tool DAVID (https://david-d.ncifcrf.gov/). The number of the enriched genes in each GO term was depicted over bars. * P < 0.05. ** P < 0.01. Figure S3. Kyoto encyclopedia of genes and genome (KEGG) pathway analysis. The significant pathways were shown in the bubble chart generated by R software using the cis-target mRNAs of DELs. The size of each bubble indicates the number of genes in each pathway. The color of each bubble represents enrichment P value. Figure S4. The full-length of LOC102167901 was obtained by rapid amplification of cDNA ends (RACE). a Nested PCR amplified product obtained from 5′-RACE and 3′ RACE assay was found to have 1021 bp (lane 2) and 1000 bp (lane 1), respectively. DNA marker DL2000 is shown in lane M. b The full-length sequence of the novel transcript. Figure S5. The expression prolife pattern of the porcine NORHA. The expression of NORHA in the heart, liver, spleen, lung, kidney, stomach, intestine, muscle and ovary was detected. GAPDH acts as an internal control. M, DNA marker DL2000. Figure S6. Alignments of mature sequences of the miR-183-96-182 cluster in vertebrates. The seed region (nucleotides 2–8) was shown in the red box. Ssc, S. scrofa; hsa, H. sapiens; mmu, M. musculus; bta, B. taurus; xtr, X. tropicalis; bfl, branchiostoma floridae; ggo, G. gorilla; ptr, P. troglodytes; gga, G. gallus. Figure S6. Alignments of mature sequences of the miR-183-96-182 cluster in vertebrates. The seed region (nucleotides 2–8) was shown in the red box. Ssc, S. scrofa; hsa, H. sapiens; mmu, M. musculus; bta, B. taurus; xtr, X. tropicalis; bfl, branchiostoma floridae; ggo, G. gorilla; ptr, P. troglodytes; gga, G. gallus [file 40104_2021_626_MOESM2_ESM.doc]

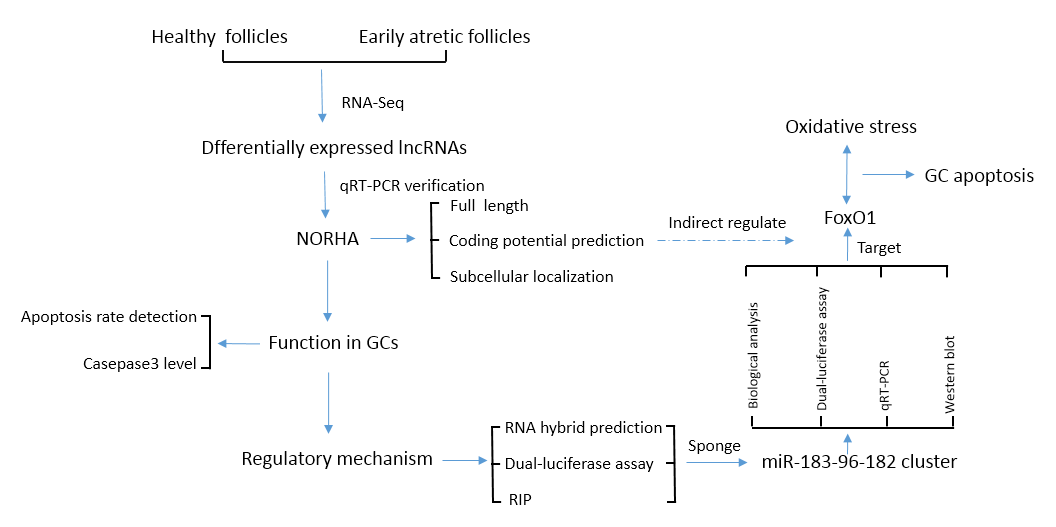


**Figure S1. Overview of experiment design.**

Flow diagram showing the experimental design and methods. The intermittent blue arrow represents the indirect regulation between NORHA and FoxO1.


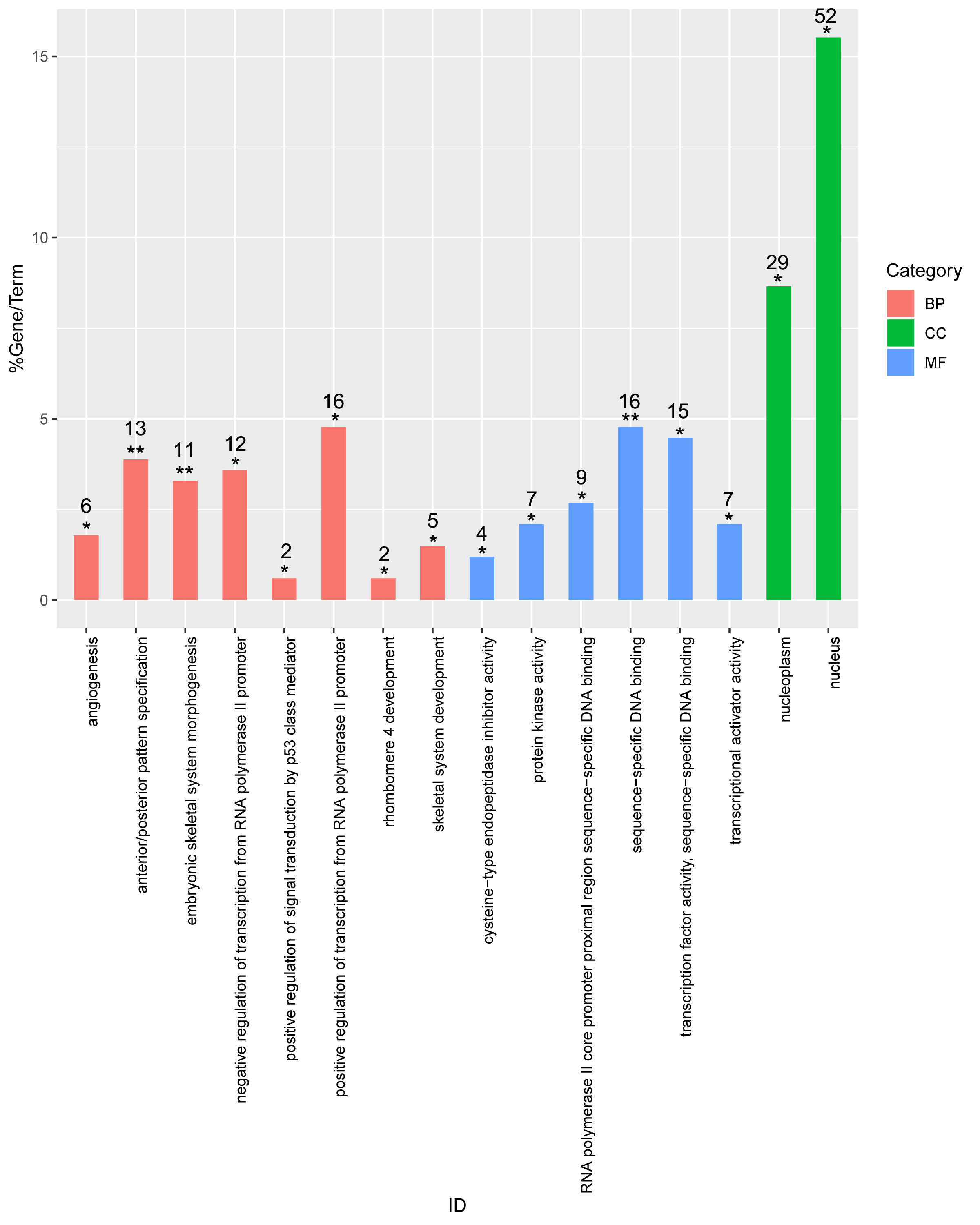


**Figure S2. Gene ontology (GO) analysis.**

GO functional enrichment of the *cis*-target mRNAs for all DELs was performed by an online tool DAVID (https://david- d.ncifcrf.gov/). The number of the enriched genes in each GO term was depicted over bars. * *P*<0.05. ** *P*<0.01.


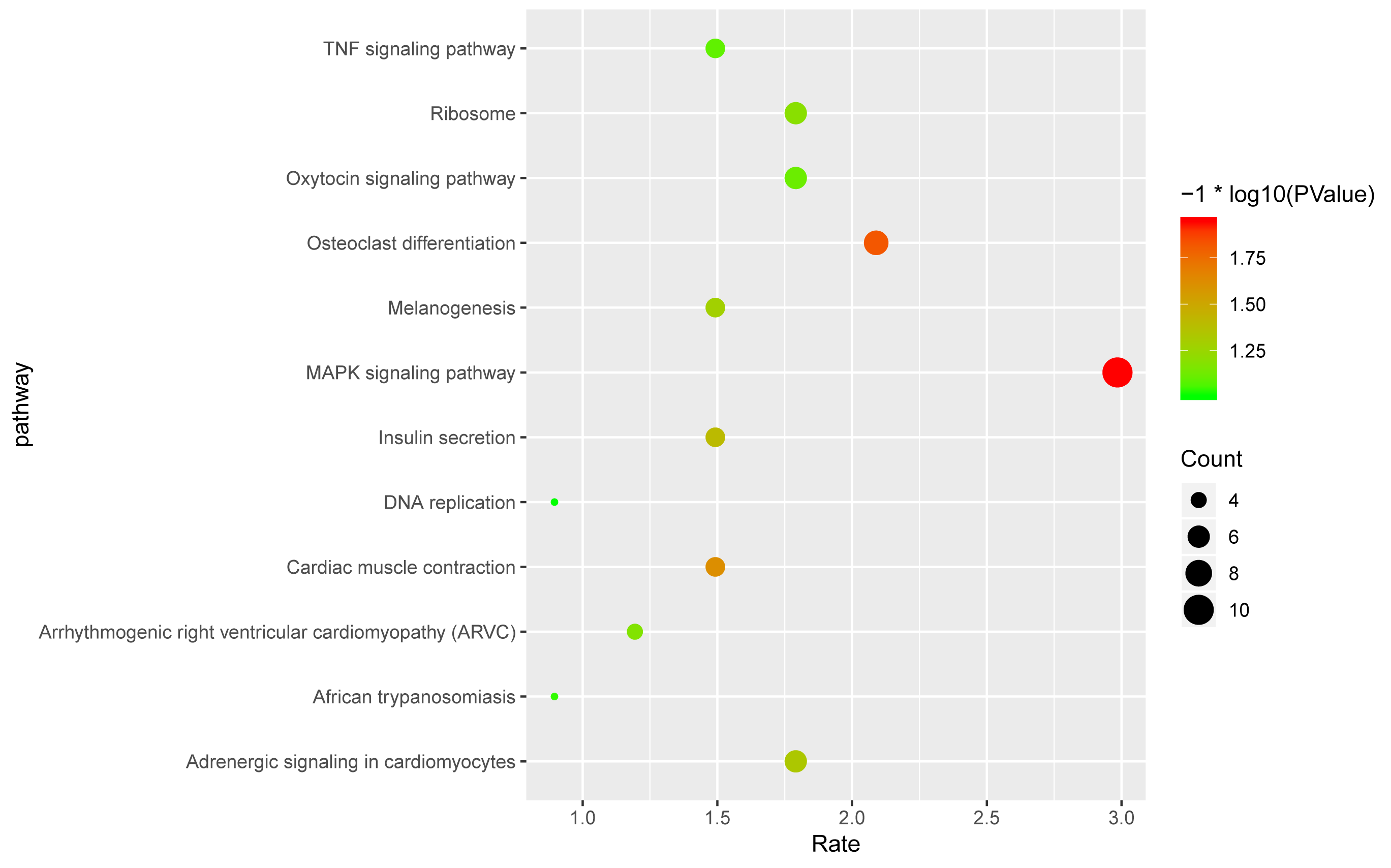


**Figure S3.** **Kyoto encyclopedia of genes and genome (KEGG) pathway analysis.**

The significant pathways were shown in the bubble chart generated by R software using the *cis*-target mRNAs of DELs. The size of each bubble indicates the number of genes in each pathway. The color of each bubble represents enrichment *P* value.


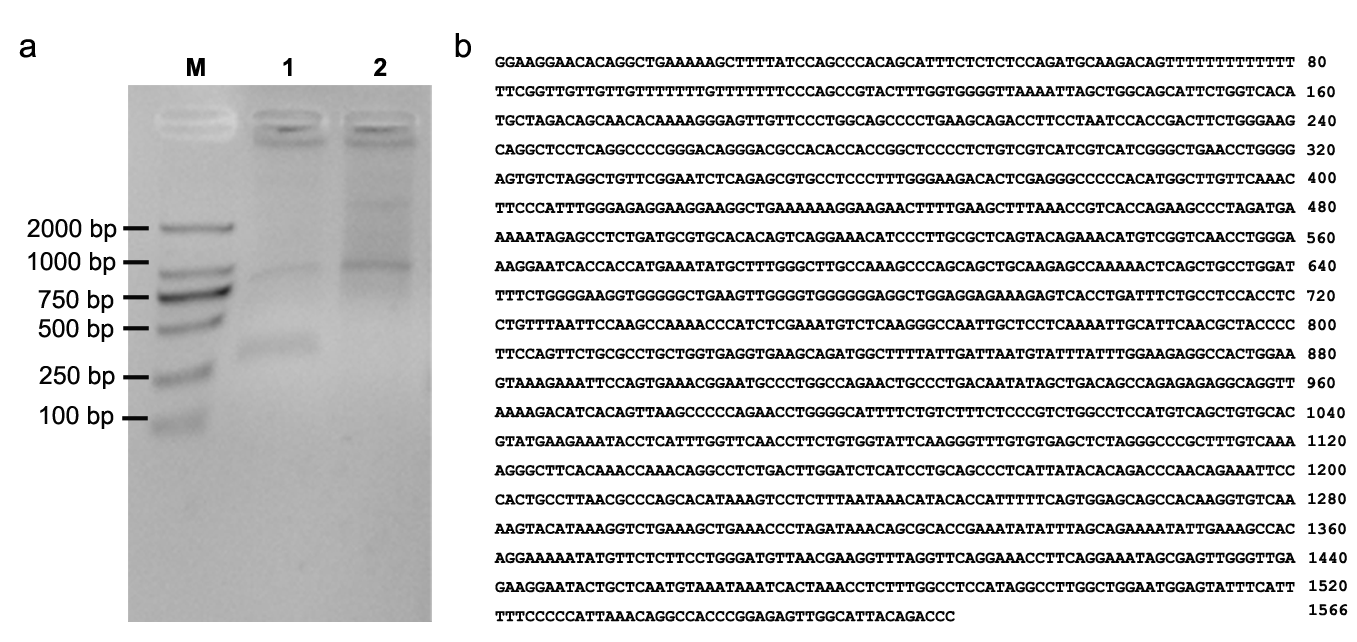


**Figure S4.** **The full-length of LOC102167901 was obtained by rapid amplification of cDNA ends (RACE).**

**a** Nested PCR amplified product obtained from 5'-RACE and 3' RACE assay was found to have 1021 bp (lane 2) and 1000 bp (lane 1), respectively. DNA marker DL2000 is shown in lane M**. b** The full-length sequence of the novel transcript.


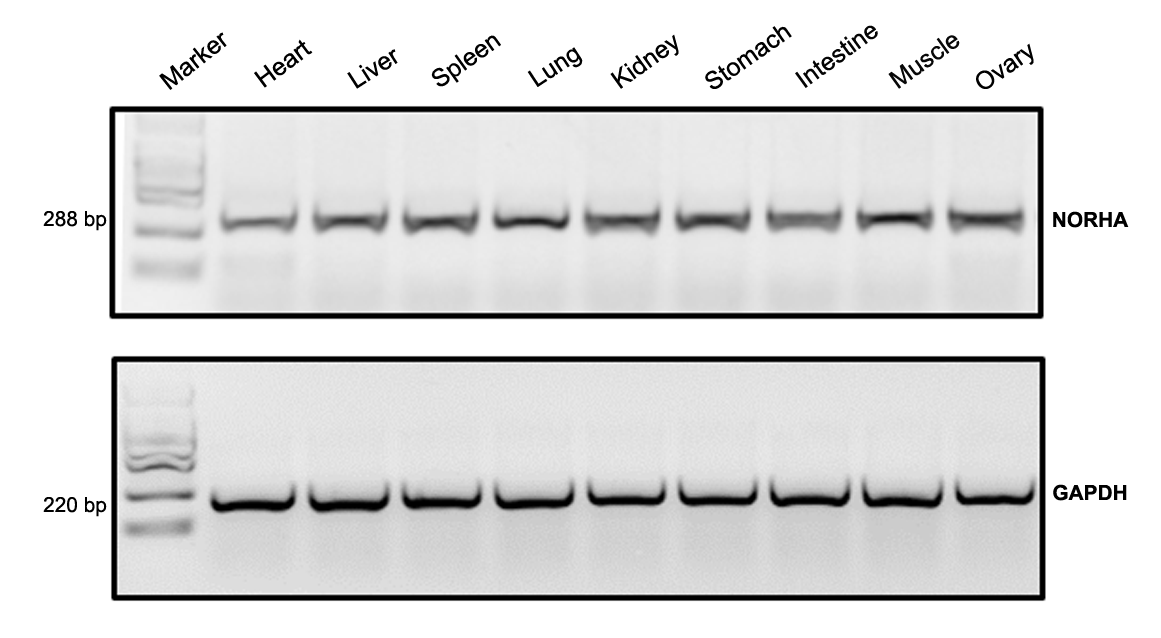


**Figure S5.** **The expression prolife pattern of the porcine NORHA.**

The expression of NORHA in the heart, liver, spleen, lung, kidney, stomach, intestine, muscle and ovary was detected. GAPDH acts as an internal control. M, DNA marker DL2000.


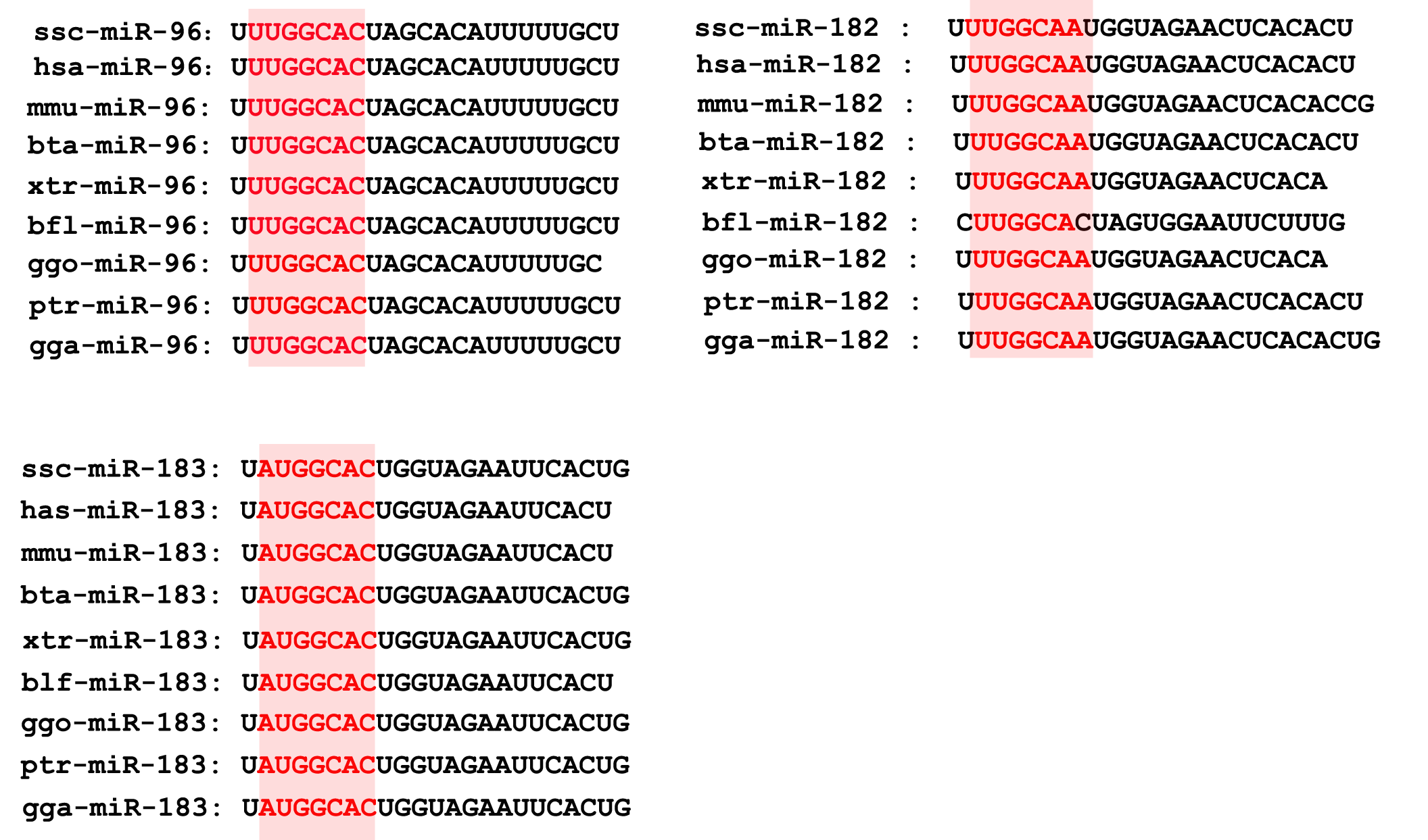


**Figure S6. Alignments of mature sequences of the miR-183-96-182 cluster in vertebrates.**

The seed region (nucleotides 2–8) was shown in the red box. ssc, *Sus scrofa*; hsa, *Homo sapiens*; mmu, *Mus musculus*; bta, *Bos taurus*; xtr, *Xenopus tropicalis*; bfl, *branchiostoma floridae*; ggo, *Gorilla gorilla*; ptr, *Pan troglodytes*; gga, *Gallus gallus*.


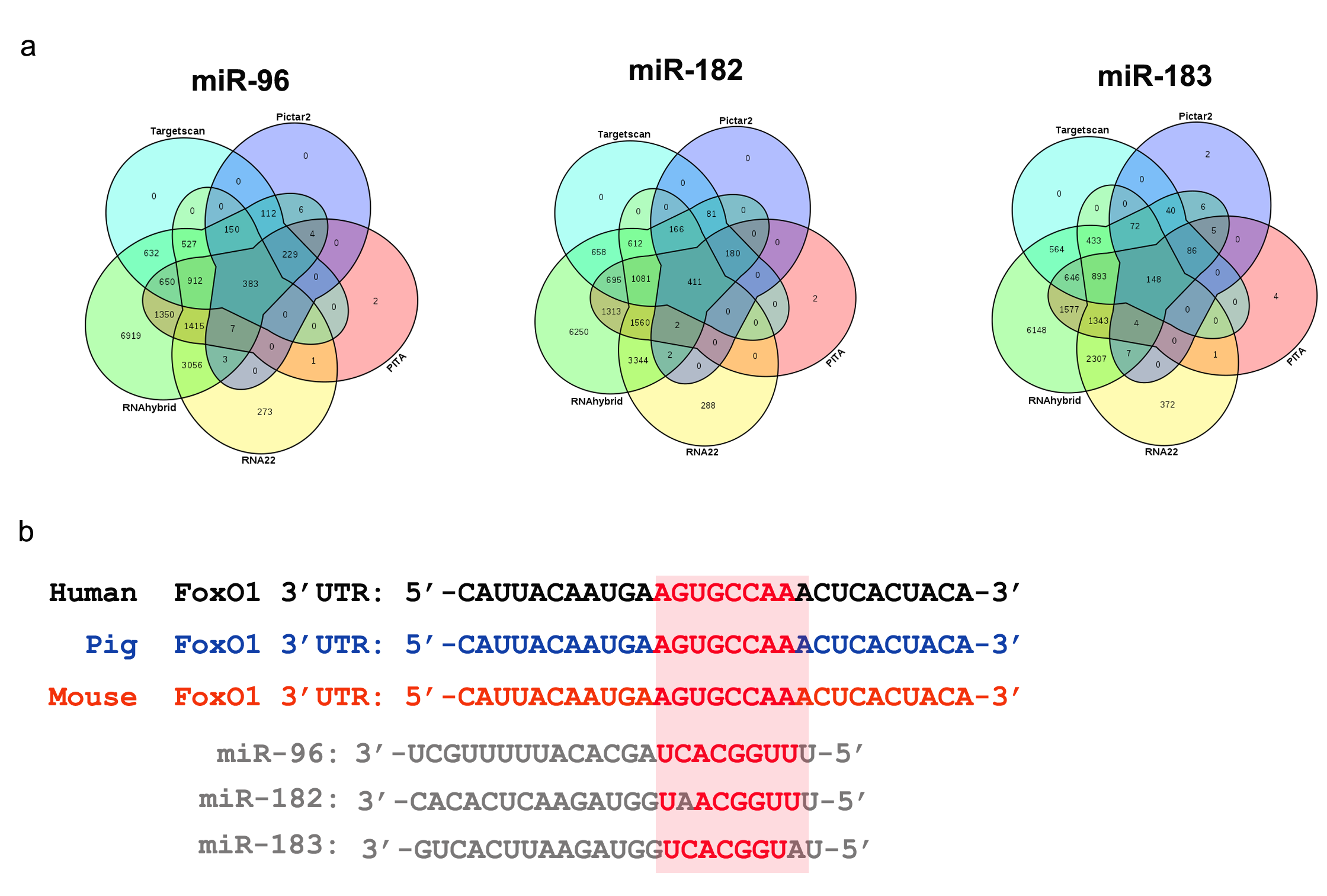


**Figure S7.** **FoxO1 is a common candidate target of the miR-183-96-182 cluster.**

**a** The Venn diagram showing the potential targets of miR-183, miR-96, and miR-182. The potential targets of miR-183, miR-96, and miR-182 were predicted by using five online tools Targetscan, Pictar2, PITA, RNA22, and RNAhybrid, respectively. **b** Sequence alignment of binding-site of the miR-183-96-182 cluster within the 3’-UTR of FoxO1 gene from humans, pigs, and mice. The red box region indicates the seed region.


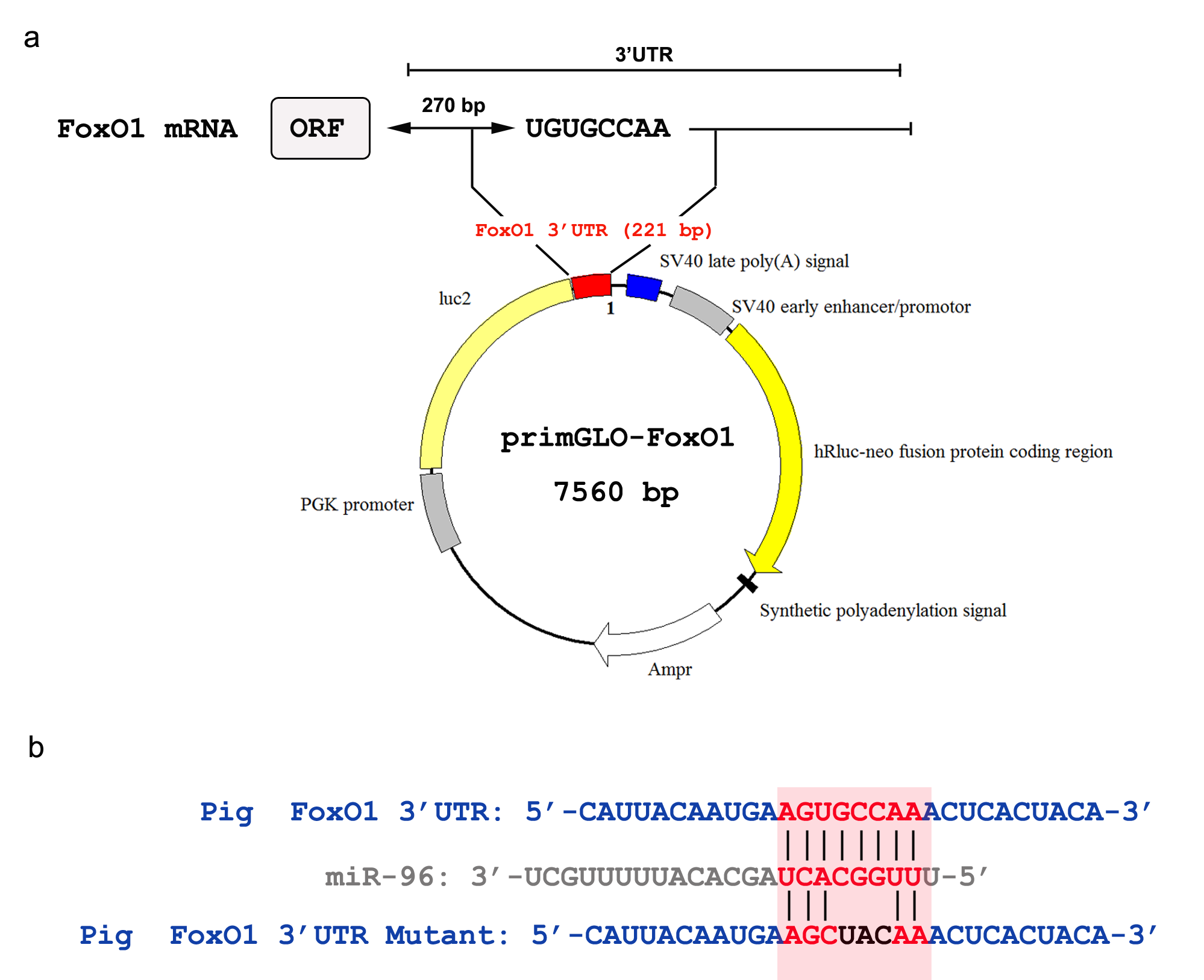


**Figure S8.** **Construction of FoxO1 3’-UTR reporter vector.**

**a** Schematic illustration shows that the primGLO reporter vector of FoxO1 3’-UTR containing the binding site of the miR-183-96-182 cluster. **b** Reporter vector of FoxO1 3’-UTR containing wild type and mutant type binding site of the miR-183-96-182 cluster.

**
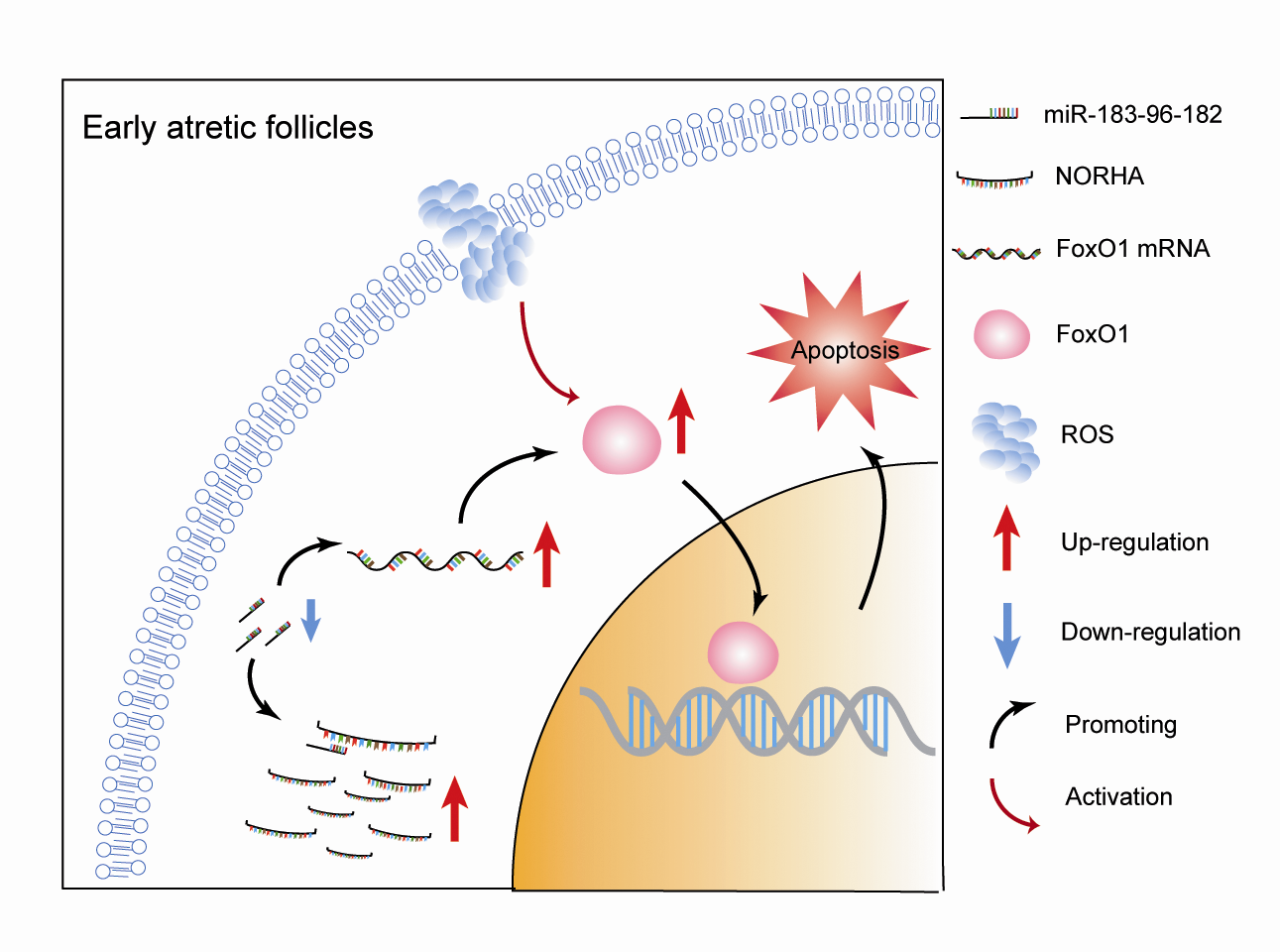
**

**Figure S9. The regulatory model of NORHA, miR-183-96-182 cluster and FoxO1 in healthy follicles and early atretic follicles.**

In early atretic follicles, levels of NORHA and oxidative stress are increased; up-regulated NORHA induces FoxO1 (an effector of oxidative stress) by acting as a sponge of the miR-183-96-182 cluster (the cluster inhibits FoxO1), and then NORHA, synergistically with FoxO1, induces apoptosis of granulosa cells.
